# Biomimetic Chiral Nanomaterials with Selective Catalysis Activity

**DOI:** 10.1002/advs.202306979

**Published:** 2024-04-01

**Authors:** Honghui Cao, En Yang, Yoonseob Kim, Yuan Zhao, Wei Ma

**Affiliations:** ^1^ School of Perfume and Aroma Technology Shanghai Institute of Technology No. 100 Haiquan Road Shanghai 201418 China; ^2^ School of Food Science and Technology, State Key Laboratory of Food Science and Resources Jiangnan University Wuxi Jiangsu 214122 China; ^3^ Key Laboratory of Synthetic and Biological Colloids Ministry of Education, School of Chemical and Material Engineering Jiangnan University Wuxi Jiangsu 214122 China; ^4^ Department of Chemical and Biological Engineering The Hong Kong University of Science and Technology Clear Water Bay Hong Kong SAR 999077 China

**Keywords:** biomimetic nanomaterials, chiroptical effects, selective catalysis activity

## Abstract

Chiral nanomaterials with unique chiral configurations and biocompatible ligands have been booming over the past decade for their interesting chiroptical effect, unique catalytical activity, and related bioapplications. The catalytic activity and selectivity of chiral nanomaterials have emerged as important topics, that can be potentially controlled and optimized by the rational biochemical design of nanomaterials. In this review, chiral nanomaterials synthesis, composition, and catalytic performances of different biohybrid chiral nanomaterials are discussed. The construction of chiral nanomaterials with multiscale chiral geometries along with the underlying principles for enhancing chiroptical responses are highlighted. Various biochemical approaches to regulate the selectivity and catalytic activity of chiral nanomaterials for biocatalysis are also summarized. Furthermore, attention is paid to specific chiral ligands, materials compositions, structure characteristics, and so on for introducing selective catalytic activities of representative chiral nanomaterials, with emphasis on substrates including small molecules, biological macromolecule, and in‐site catalysis in living systems. Promising progress has also been emphasized in chiral nanomaterials featuring structural versatility and improved chiral responses that gave rise to unprecedented chances to utilize light for biocatalytic applications. In summary, the challenges, future trends, and prospects associated with chiral nanomaterials for catalysis are comprehensively proposed.

## Graphical abstract

In this work, chiral nanomaterials synthesis, composition, and catalytic performances of different biochemical hybrid chiral nanomaterials are discussed. The construction of chiral nanomaterials with multiscale chiral geometries along with the underlying principles for enhancing chiroptical responses are highlighted. Various biochemical approaches to regulate the selectivity and catalytic activity of chiral nanomaterials for catalysis are summarized. Special attention is paid to specific chiral ligands, materials compositions, and structure characteristics for introducing selective catalytic activities of representative chiral nanomaterials.

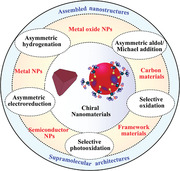

## 1 Introduction

Chirality is the basic property in nature, and many biomolecules are chiral, including amino acids, peptides, proteins, DNA, phospholipids sugars, etc. Mimicking chiral molecules, artificial chiral nanomaterials with large surface‐to‐volume ratios have been widely studied and showed tunable chemical and physical functions, displaying potential bioapplications in nonlinear optics, polariton chemistry, and quantum technology.^[^ ^1^, ^2^ ^]^ Chiral biomolecules with a size smaller than the light wavelength generally showed weak chiral signal, while artificial chiral nanomaterials concentrated the light within a particular area could strengthen light‐matter interaction and display enhanced chiral responses.^[^ ^3^, ^4^ ^]^ Chiral nanomaterials were expanded from single chiral NPs to artificial chiral assembled superstructures^[^ ^5^ ^]^ with chirality evolution from the chirality transfer from chiral molecules modified on NPs to the chiral geometry of nanomaterials.^[^ ^6^, ^7^, ^8^, ^9^ ^]^ In particular, metal nanoparticles (NPs), metal oxide NPs, semiconductors, and other chiral nanomaterials constituted the majority of chiral nanomaterials and demonstrated high catalysis activity and selectivity.^[^ ^10^, ^11^ ^]^ Chiral nanomaterials are robust and efficient and have enormous potential for biocatalytic applications compared to traditional chiral catalysts.

The natural enzymes usually possess structural complexity, and the precise specific active sites are still vague regarding the functional components in some enzymes.^[^ ^12^, ^13^ ^]^ For expanding catalysis applications in broader fields, designing and synthesizing artificial to submicron scale is an exciting challenge. In comparison with natural enzymes, artificial nanozymes, like chiral nanomaterials, are stable and can be applied in a large range of pH and temperatures. Artificial nanozymes also have the advantages of high yield with low cost and a long storage time without being readily degraded.^[^ ^14^, ^15^ ^]^ Therefore, it is crucial for future demand to develop chiral encoded artificial nanozymes with enhanced selectivity. Chiral nanomaterials modified by chiral ligands possess distinct recognition abilities toward chiral enantiomers and show potential application for enantioselective catalysis.^[^ ^16^ ^]^ For biomimetic nanomaterials, the structural effect including tunable size, suitable morphology, and preference crystal facet could be involved in regulating selective catalytic interaction.^[^ ^17^ ^]^ The anchoring ligand on the sites of geometrical nanomaterials might synergistically limit its aggregation. The synergistic effect of nanoparticles and chiral ligands in chiral nanomaterials plays a key role in achieving superior catalytic performance.^[^ ^18^, ^19^ ^]^ Overall, the dimensional matching, preferred modification, and catalysis active sites helped to promote catalysis activity and selectivity to corresponding substrates.

Heterogenous compositions of nanoparticles helped to enhance optical, electrical, and magnetic responses that could benefit for enhancing catalysis.^[^ ^20^, ^21^, ^22^ ^]^ For better performance of separation from substrate, chiral nanostructures can be extended to submicron and micron scale, when for instance thin nanoscale assembled solid film has a micron scale.^[^ ^23^, ^24^, ^25^ ^]^ The manifestation of rotatory optical activity benefited from highly efficient catalysis.

The chiral NP cores in most cases play an accelerating role in catalytic activity, and enantioselectivity is associated with the chiral molecule‐NP interface layers. Generally, the inorganic core provided hot electrons and holes for promoting the catalytic chemical reactions.^[^ ^26^ ^]^ Chiral nanomaterials served as artificial nanoenzymes and demonstrated multiple mimic enzyme activities by adjusting the compositions, structures, and chiral ligands. In comparison to natural enzymes, artificial chiral nanoenzymes not only showed tunable catalytic activity but also could have well‐catalytic behaviors in harsh acidic tumor microenvironments.^[^ ^27^ ^]^ In order to enhance the biocatalytic efficiency, various chiral nanomaterials were fabricated, and the chiroptical responses were attempted to be improved by adjusting the chiral ligands and controlling the morphology of chiral nanomaterials.^[^ ^28^ ^]^ With enhanced chiral responses, chiral nanomaterials can utilize the circularly polarized light (CPL), such as left or right CPL (donated as LCP, RCP) to produce more hot electrons and reactive oxygen species (ROS),^[^ ^29^, ^30^ ^]^ which is used for biological macromolecule catalysis and in‐site biocatalysis in living systems.

This review covers the design, fabrication, chiroptical effects, and selective catalysis activity of chiral nanomaterials including chiral metal NPs,^[^ ^7^, ^31^, ^32^ ^]^ chiral metal oxide NPs,^[^ ^33^, ^34^, ^35^ ^]^ chiral semiconductors,^[^ ^36^, ^37^ ^]^ chiral carbon materials,^[^ ^38^, ^39^ ^]^ chiral framework materials,^[^ ^40^, ^41^ ^]^ as well as chiral assembled nanostructures.^[^ ^8^, ^42^, ^43^ ^]^ The synthetic methodology regarding chemical precursors, chiral ligands, growth conditions, and controlling of material compositions and geometries was comprehensively discussed. In the current review, we assess different types of selective catalysis activities including asymmetric hydrogenation,^[^ ^44^, ^45^ ^]^ asymmetric Aldol reaction,^[^ ^46^, ^47^ ^]^ selective oxidation,^[^ ^48^, ^49^ ^]^ asymmetric electroreduction,^[^ ^38^ ^]^ and so on. The applications of chiral nanomaterials in enantioselective biocatalysis in‐site in living systems were also explored. Additionally, we prospected the future advances and potential challenges of chiral nanomaterials in the emerging catalysis fields.

## 2 Construction of Chiral Nanomaterials and Chiroptical Activities

### 2.1 Chiral metal NPs

Plasmonic metal NPs possess unique localized surface plasmon resonance (LSPR) properties.^[^ ^50^, ^51^ ^]^ They can absorb and scatter light and usually show bisignate circular dichroism (CD) in the visible regions.^[^ ^52^, ^53^ ^]^ Recent advances in the construction of chiral metal NPs by using chiral molecules such as amino acids offered a completely novel pathway to achieving chirality in a single NP.^[^ ^54^, ^55^, ^56^ ^]^ The functionalization of chiral ligands on metal NPs induces surface molecular integrated chirality at the absorption site as enantiomorphic domain for catalysis applications.^[^ ^3^, ^57^ ^]^ The chiral metal NPs bearing chiral molecules or owned chiral geometries can be described as a coupled system with induced electric and magnetic dipoles that interact differently with LCP and RCP light. The molecule types (small molecules or macromolecules)^[^ ^58^, ^59^, ^60^, ^61^ ^]^ and geometries (defects, helicoid or twisted shape)^[^ ^32^, ^62^, ^63^, ^64^, ^65^, ^66^ ^]^ contribute if the plasmon mode can be excited and coupled more efficiently by one circular polarization light than the other.^[^ ^67^, ^68^ ^]^ The symmetry‐breaking is important to trigger large chiroptical responses and catalysis activity.^[^ ^69^, ^70^ ^]^ The magnitude of the induced dipoles in plasmonic NPs is strong, resulting in an intensive chiroptical effect.

There are several general methods for the preparation of metal NPs with surface ligands that differ by the nature of chemical reactions:^[^ ^3^, ^71^ ^]^ a) chemical reduction of salts, b) displacement from organometallic complexes, c) electrochemical reduction, d) thermal, photochemical, or sonochemical decomposition of organometallic precursors. The aqueous metal precursors usually commonly use tetrachloroauric (III) acid, silver nitrate, and so on, while the most typical reduction reagents are sodium borohydride (NaBH_4_) and sodium citrate.^[^ ^54^, ^64^ ^]^ The use of chiral biomolecules including amino acids, peptides, protein or DNA, etc., was invaluable for inducing and manipulating chiroptical activity in metal nanomaterials, in which the chiral interface was not well understood for the complexity of molecule−NPs interaction.^[^ ^3^, ^71^, ^72^, ^73^, ^74^ ^]^ Generally, the heterogenous metal structures with strong Plasmonic resonance facilitate to inducement of strong chiroptical signals.^[^ ^61^, ^75^, ^76^ ^]^ It is reported that complex interactions between D‐peptides and Au NPs lead to a chiral restructuring of peptides and proteolytic cleavage of D‐peptides via gold‐mediated inversion of peptide chirality.^[^ ^59^ ^]^ The DNA as an environment‐responsive chiral ligand was also used to fabricate chiral Au NPs for engineering selective catalytic behaviors.^[^ ^60^ ^]^ The chiral ligand is devoted to both selective recognition of substrate and catalytic activity.

Heterogenous metal composition helped to enhance the chiroptical effect with specific structures, such as the strong eciton− plasmon coupling in Au@Ag NPs complexes.^[^ ^77^ ^]^ In order to obtain intense chiral response, DNA was embedded in the junction of plasmonic Au core and Ag shell with the aid of Ag^+^.^[^ ^61^ ^]^ By utilizing the high association between DNA and Ag^+^, Ag^+^ was further reduced to an Ag shell. The DNA‐engineered Au@Ag core‐shell NPs exhibited giant chiroplasmonic signals in the regions of 500 nm to 550 nm, owing to the core‐shell enhanced electric field for strong chiral responses. To further enhance the chiroplasmonic responses and obtain mirror CD signals, gapped core‐shell NPs were designed by embedding amino acids in the gaps. After modifying cysteine (Cys) enantiomers in the gaps, the Au‐gap‐Ag nanostructures displayed intense CD responses from 350 nm to 500 nm.^[^ ^76^ ^]^ The cys‐plasmon dipolar interaction and plasmonic hot‐spot‐driven chiral amplification promoted the amplification of chiral signals. Alternatively, chiral amino acids were widely utilized as chiral selectors for the fabrication of chiral nanomaterials. In particular, thanks to the stable and strong covalent bond of metal‐SH, Cys enantiomers, and Cys‐contained peptides were generally applied for the modification on metal NPs surfaces. Cys enantiomers functionalized chiral Au NPs@Ag, Ag decahedral NPs, and Ag nanorods were all fabricated and showed enhanced chiral behaviors in the visible regions.^[^ ^78^, ^79^ ^]^ Dipeptide ligands (cysteine‐phenylalanine, Cys‐Phe) were used for the preparation of chiral AuCuAu nanorods (NRs). Chiral AuCuAu NRs showed intense CD intensity in the regions of 400 nm‐1000 nm. The introduction of low‐cost and earth‐abundant Cu could primarily improve the plasmonic catalytic function and control the LSPR signals.^[^ ^80^ ^]^ The strong chiral properties enabled chiral AuCuAu NRs to utilize the circularly polarized light, which generated more ROS than Au NRs for biocatalytic applications. Chiral metal NPs with intense electromagnetic fields could produce energetic charge carriers and utilize light irradiation for chemical transformation. The increasing concentration of modified chiral amino acids significantly boosted the intensity of CD responses of plasmonic NP nanostructures, and the high density of chiral amino acids endowed chiral metal NPs with high selectivity.

**Figure 1:**
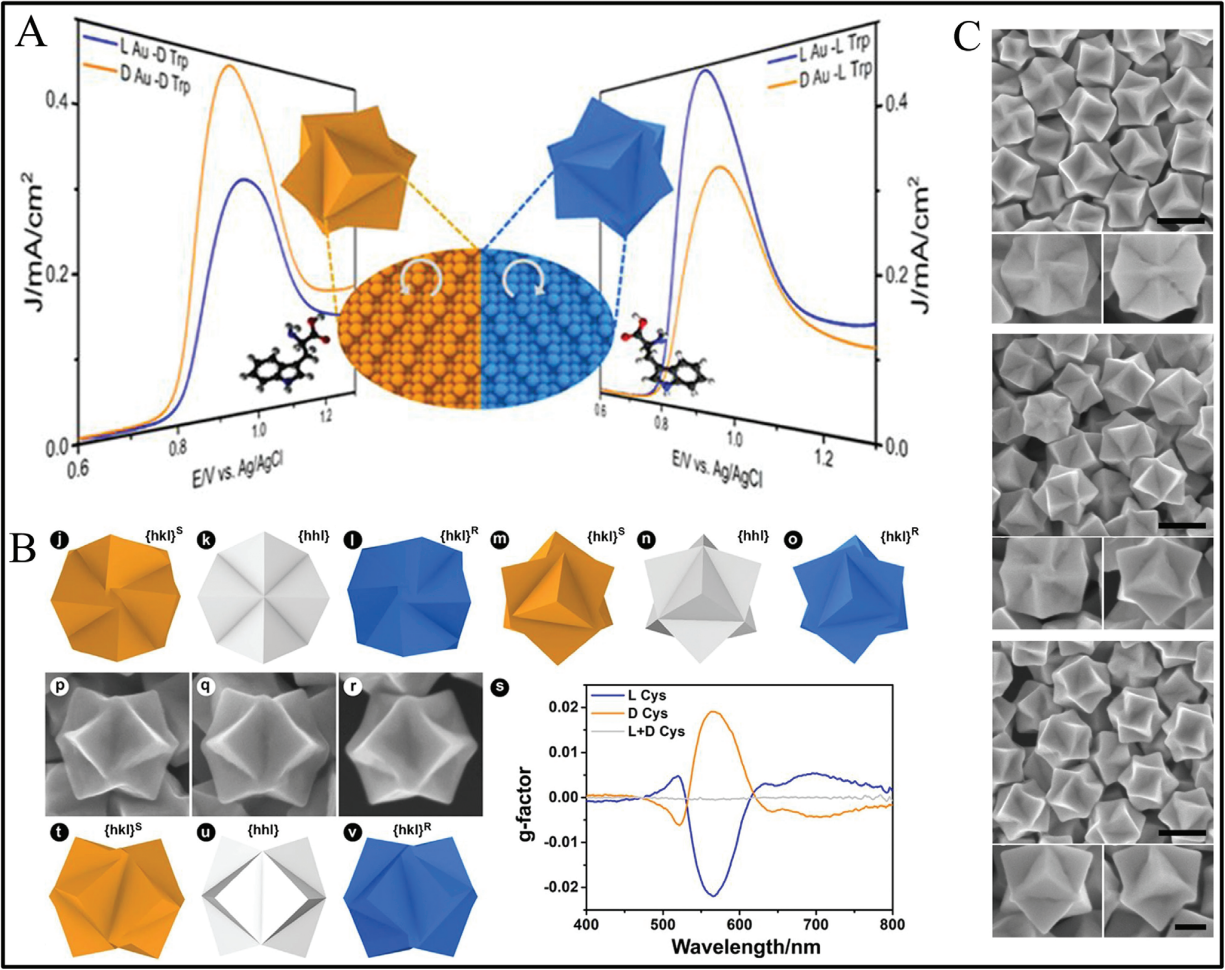
A) CD spectra of chiral Au NPs with precise homochiral facets for chiral discrimination of L‐/D‐tryptophan. B) SEM images and corresponding geometric models of facets from D‐Cys, racemic Cys, and L‐Cys. g‐Factor spectra of chiral and achiral Au nanocrystals. C) SEM images of chiral trisoctahedral homochiral facets from D‐Cys, trisoctahedral from racemic Cys, and chiral trisoctahedral homochiral facets from L‐Cys, respectively. Upper scale bars: 200 nm, Downer scale 100 nm.^[^ ^64^ ^]^

High amounts of chiral ligands modified on the metal NP surface may also cover and block the catalytic active sites. The extra modification of chiral ligands on metal NPs may not be stable for sustainable and durable catalytic applications. Alternatively, chiral nanomaterials featured with chiral geometries could produce amplified chiral responses by utilizing the molecular interaction of chiral molecules (amino acids or peptides) and demonstrating good catalytic behaviors. The existence of chiral ligands caused the synthesis of twisted chiral geometry in the synthesis process of chiral metal NPs, which further endowed chiral metal NPs with remarkable CD signals.^[^ ^3^, ^62^, ^63^ ^]^ The configuration of introduced chiral ligands determined the handedness of chiral geometry. In particular, Cys enantiomers induced the formation of cube‐like Au NPs with side sizes of 150 nm and the appearance of split edges.^[^ ^54^ ^]^ The helicoid surface of chiral Au NPs induced the generation of giant chiral signals. Whereas, glutathione (GSH) enantiomers promoted distinct pinwheel‐like chiral morphology. The different molecular sizes induced different absorption for GSH and Cys toward NPs. Cys with small molecular sizes combined with one kink, but GSH would combined with more kinks due to the large molecular sizes. This induced the formation of different chiral geometry. Under the irradiation of circularly polarized light, chiral‐shaped nanostructures were controllably prepared through the adjustment of chiral ligands. The rhombic dodecahedral‐shaped chiral Au NPs were obtained when Cys enantiomers were added during the synthesized process. Cys enantiomers promoted the transformation from low‐miller‐index NPs to high‐miller‐index NPs.^[^ ^81^ ^]^ During the growth process, L‐ or D‐Cys, as a chiral surface inducer, breaks the mirrorsymmetry of Au spherical seeds and leads to Au nanocrystals with opposite chiral morphologies. In contrast, achiral Au nanocrystals are obtained with racemic Cys (50% L‐Cys +50% D‐Cys).^[^ ^64^ ^]^ Chiral Au NPs with diameters of ≈120 nm were synthesized in the presence of Cys‐Phe dipeptides upon the irradiation of 594 nm left or right circularly polarized light (**Figure** **1**A–C).^[^ ^82^ ^]^ Light irradiation induced the transformation from low‐index crystal planes to high‐index crystal planes and promoted the formation of anticlockwise rotation of L‐Au NPs and clockwise rotation D‐Au NPs. The light irradiation also promoted the formation of chiral helical Au nanoarrows by applying L‐selenocystine as a chiral inducer.^[^ ^83^ ^]^ The cleavage of diselenide induced the appearance of selenyl radicals under light illumination, resulting in the formation of helical Au nanoarrows. Similarly, the Cys and penicillamine (Pen) were also exploited on the structures of Au nanoarrows. Only L‐selenocystine under light irradiation and Cys enantiomers with a proper concentration could promote the generation of helical Au nanoarrows. Chiral nanomaterials featured with chiral morphology demonstrated stronger chiroptical responses for sensitive biocatalytic applications. Therefore, the construction of complex metal NPs would show cooperative catalytic activity, such as core‐shell and yolk‐shell structures.^[^ ^84^, ^85^, ^86^, ^87^ ^]^ The rational design of complex chiral nanomaterials will be a hot topic through adjusting the heterogeneous metal components and exploiting the morphology with more catalytic sites and high active centers.

### 2.2 Chiral Metal Oxide NPs

In order to enhance the biocatalytic activity, the integration of components with high catalytic active sites was significant for the design of chiral metal oxide NPs.^[^ ^88^ ^]^ Alternatively, metal oxide NPs were cheaper than novel metal NPs and generally displayed excellent catalytic ability. Chiral ligands were functionalized on the surface of Fe_3_O_4_ NPs, Cr_2_O_3_ NPs, TiO_2_ NPs, and so on.^[^ ^89^, ^90^ ^]^ Chiral metal oxide NPs have received tremendous attention in bio‐applications owing to their intriguing optical, physical, and surface chemical and catalytic properties, especially for chiroptical properties.^[^ ^91^, ^92^, ^93^ ^]^

The benefits of chiral magnetic NPs are numerous and have been reported by different synthesis routes.^[^ ^94^, ^95^ ^]^ For instance, functionalization is facilitated by reduced size and greater specific surface area; the magnetic properties are simple to recycle, can be used again, and have outstanding performance in the heterogeneous catalytic system, low toxicity, and affordable.^[^ ^95^, ^96^ ^]^ An ideal functional material having chirality and magnetic characteristics can be created by combining chiral components. The following techniques can be used to create magnetic NPs: coprecipitation, thermal decomposition, microemulsion, hydrothermal, and biological approaches.^[^ ^97^, ^98^ ^]^ Aggregation and oxidation are common in magnetic NPs. It is possible to modify magnetic NPs by coating them with various chiral molecules that offer both protection and binding sites. Chiral small molecules and chiral macromolecules are two categories of chiral magnetic NPs.^[^ ^33^, ^99^ ^]^ A typical chiral small molecule catalyst is amino acids, in which L‐proline (L‐Pro) has the capacity to catalyze reactions with great stereoselectivity. It is readily retrieved from the reaction mixture when mixed with a magnetic material, preserving its catalytic activity and selectivity even after repeated applications.^[^ ^100^ ^]^ The chiral magnetic NPs that were produced were both highly chiral catalytically active and recyclable. Multiple recycling without noticeably losing activity. Proline derivatives (L‐4‐hydroxyproline) were then changed on the carrier after magnetic NPs were first coated with silica to create a carrier. Chiral macromolecules contain more intricate structures, which makes it easier to alter the composition and physical characteristics of created materials.^[^ ^101^ ^]^ Chiral biopolymers and chiral synthetic polymers are two categories of chiral macromolecules. Chiral compounds can form covalent or non‐covalent bonds with carriers on the surface of magnetic NPs. The technique of non‐covalent bonding is straightforward but unstable. The quantity of chiral ligands through linking by number of carrier active sites usually has less amount compared to non‐covalent adsorption, but the interaction between chiral compounds connected by covalent bonds is significant, which may be improved by expanding the number of active sites. For example, based on amino acid‐appended chiral polymer, Qu's group fabricated stereoselective nanocatalyst (Fe_3_O_4_@Poly(amino acid)) using ferromagnetic Fe_3_O_4_ core as the catalytic active sites and amino acid encoded chiral polymer shell as the chiral binding coupled selector.^[^ ^102^ ^]^ This magnetic nanocatalyst was fabricated through Fe_3_O_4_ core NPs synthesis, silica shell coating Fe_3_O_4_ (Fe_3_O_4_@SiO_2_), then chiral polymer shell modification (Fe_3_O_4_@SiO_2_@Poly(L‐/D‐Trp)), and remove of silica shell by etching using concentrated NaOH to obtain yolk–shell Fe_3_O_4_@Poly(L‐/D‐Trp). It was found that the polymer shell displayed a rough surface in which the polyacrylate(amino acid) displayed as selective access to substrate and enabled enantioselective catalysis.

Chiral magnetic NPs also was reported synthesized in the organic phase, such as chiral FePd NPs with Fe_x_O_y_‐rich core and Pd‐rich shell was fabricated through iron decomposition of carbonyl iron, subsequent reducing palladium acetylacetonate (Pd(acac)_2_) in oleic acid and oleylamine.^[^ ^103^ ^]^ By ligand exchange reaction with chiral 2, 20‐bis (diphenylphosphino)−1, 10‐binaphthene (BINAP), the resulting NPs showed optical activity at 300–550 nm, with a typical Cotton effect. Interestingly, the external magnetic field induces polarization of paramagnetic NPs, in which the spin and orbital magnetic moments are preferentially oriented in the direction of the field. This effect would be small for high symmetry crystal lattices (e.g., cubic), due to dielectric permittivity and magnetic susceptibility tensors being less anisotropic.^[^ ^104^, ^105^ ^]^ Alternatively, when magnetic Fe_3_O_4_ NPs integrated with semiconductor‐metal ZnxCd_1−x_S‐Ag_2_S/Au NRs, the hybrid NRs exhibited optical activity at 250–600 nm owing to the angle deflection between electric and magnetic transition dipole moment.^[^ ^106^ ^]^ However, under an external magnetic field, the magnetic CD was independent of the applied magnetic field intensity due to the magnetization of the hybrids.

Current synthesis methods for other metal oxides are mostly based on the use of chiral biomolecules such as amino acids or peptides. Based on phenylalanine (Phe) as the structure‐directing molecules, the uniform chiral Cu_x_O NPs were fabricated with an average size of 65 nm and displayed optical activity at the spectra region of 200–350 nm.^[^ ^107^ ^]^ Chiral WO_3‐x_·H_2_O NPs were fabricated by utilizing Asp and Pro as ligands through the formation of C‐O‐W linkages. 1.6 nm chiral WO_3‐x_·H_2_O NPs were added in ethanol and demonstrated the CD bands at 400–700 nm for chiral Pro‐WO_3‐x_·H_2_O NPs and 500–1100 nm for chiral Asp‐WO_3‐x_·H_2_O NPs, respectively.^[^ ^108^ ^]^ This was because the electronic transitions were changed due to the chiral distortions of atomic packing on WO_3‐x_·H_2_O NPs, but the additional C‐O‐W linkage for Asp led to higher distortion of the inorganic crystal lattice and stronger CD intensity. Chiral metal oxide Cr_2_O_3_ NPs multilayer made by using block copolymer inverse micelle and R/S‐mandelic acid via selective incorporation of precursors within micellar cores followed by the oxidation process had been reported with intense CD signals at spectra region of 300–800 nm, with a g‐factor up to 7.0*10^−3^.^[^ ^35^ ^]^ Chiral mesostructured TiO_2_ films were fabricated by hydrothermal method using L/D‐mannitol and titanium foil as the substrate and inorganic precursor.^[^ ^92^ ^]^ Chiral mesostructured TiO_2_ films displayed different levels of hierarchical chirality with intensive CD spectra region of 200–400 nm peaked at 350 nm, which could be attributed to the electronic transitions in a dissymmetric electric field. Chiral mesoporous silicates immobilizing titanium dioxide have been reported for catalytic asymmetric epoxidation of alkenes,^[^ ^91^ ^]^ in which the catalyst was prepared by doping of titanium salt and chiral additive in a sol‐gel process and further modified by chiral sulfonyl chloride. The titanium‐containing materials displayed good porosities, ordered pore size distributions, ordered morphologies with internal chiral configurations, and chiral ligands for catalytic asymmetric epoxidation of alkenes. the titanium silicates showed good conversion of alkenes (100%), satisfactory yields (98%), and ee values (100%) of epoxides. The titanium dioxide catalysts displayed satisfactory recycling behaviors and stabilities. The nanocrystalline metal oxides, MgO, CaO, Al_2_O_3,_ and ZnO are porous inorganic NPs in the 4–7 nm particle size range with unique physical and chemical properties, which owed numerous surface sites such as crystal corners, edges, or ion vacancies and displayed enhanced surface reactivity. New bifunctional catalysts composed of PdCl_4_^2−^, OsO_4_^2−^, WO_4_^2−^ and nanocrystalline MgO were prepared as bifunctional catalysts including NAP‐Mg‐PdOs and NAP‐Mg‐OsW.^[^ ^109^ ^]^ Based on the chiral ligand 1,4‐bis(9‐o‐dihydro‐ quinidinyl)phthalazine, the bifunctional catalysts exhibited tandem Heck asymmetric dihydroxylation and asymmetric dihydroxylation‐N‐oxidation reactions. During chiral NAP‐Mg‐OsW catalysis in the asymmetric dihydroxylation‐N‐oxidation, the1,4‐bis (9‐o‐dihydro‐ quinidinyl) phthalazine in a single O_2_ is used as a terminal oxidant to provide N‐methylmorpholine N‐oxide through oxidation of N‐methylmorpholine. Alternatively, based on sol‐gel and coprecipitation methods, individual SiO_2_, TiO_2_, ZrO_2_, and double oxides TiO_2_–SiO_2_, TiO_2_–ZrO_2_, ZrO_2_–SiO_2_ had been fabricated as heterogeneous catalysts for the asymmetric Biginelli reaction.^[^ ^34^ ^]^ It was shown that including molecular imprints of aromatic carboxylic acids in composites during sol‐gel synthesis provided an efficient approach for asymmetric Biginelli reaction.

### 2.3 Chiral Semiconductors and Supraparticles

Semiconductors usually possessed smaller dielectric constants than metal NPs, and the dipolar interaction was relatively weaker for the generation of chiroptical activity.^[^ ^110^, ^111^ ^]^ Chiral semiconductors were proposed and showed chirality in the visible ranges, attributed to the chiral distortion of surface atoms and the Coulomb dipole‐dipole interactions between chiral molecules and NPs.^[^ ^36^, ^112^, ^113^ ^]^ The optical and electronic properties of semiconductors could be adjusted by the composition and crystal structure,^[^ ^114^ ^]^ which further affected the chiroptical properties. Various chiral semiconductors (Cu_x_Co_y_S NPs, Fe_x_Cu_y_Se NPs, Cu_2‐x_S NPs, ZnS NPs, CdTe NPs, etc) were designed by using biomolecules as chiral ligands for catalysis.^[^ ^37^, ^115^, ^116^ ^]^ In particular, amino acids (such as Cys and Pen) and peptides were commonly utilized as chiral molecules for the preparation of chiral semiconductors.^[^ ^113^ ^]^ For chiral semiconductor NPs, circularly polarized luminescence can sometimes be accompanied by its chiroptical effects, in which such luminescence‐related electronic transition in a chiral excited polarization state due to nonzero dot product between the electric dipole transition moment and the imaginary magnetic dipole transition moment.^[^ ^117^, ^118^ ^]^

Chiral semiconductors with sizes down to the nanometer scale not only possessed broad and tunable light absorption but also could control the electronic transport for efficient catalysis applications.^[^ ^67^, ^119^ ^]^ Generally, the metal salt precursor, chiral ligand (amino acid), reductant such as thioacetamide, Na_2_SeO_3_, Na_2_S, NaHTe with pH> 10.0 was used for synthesis of chiral semiconductor NPs, which had been used for chiral CdS,^[^ ^120^ ^]^ HgS,^[^ ^121^ ^]^ CdTe,^[^ ^122^ ^]^ FexCuySe,^[^ ^123^ ^]^ and so on. Alternatively, another typical method was based on post‐synthetic covalent modification of achiral semiconductor NPs by ligand exchange to induce chirality. The original achiral semiconductor NPs either synthesized in an aqueous solution^[^ ^115^ ^]^ or through organic phase synthesis.^[^ ^124^ ^]^ The ligand exchange was through the interaction between thiol groups and metal ions. The achiral trioctylphosphine oxide,^[^ ^125^ ^]^ oleic acid^[^ ^126^ ^]^ capped semiconductor NPs that were synthesized through the hot‐injection technique.

Pen as chiral ligands mainly was applied for the fabrications of chiral Cu_x_Co_y_S NPs, chiral Fe_x_Cu_y_Se NPs, chiral Cu_2‐x_S NPs, chiral ZnS NPs, and so on.^[^ ^110^, ^123^, ^127^ ^]^ The chiral Cu_x_Co_y_S NPs with size of 2.0 ± 0.5 nm were constructed by utilizing Pen as chiral molecules and showed mirror chiral signals in the regions from 400 nm to 700 nm.^[^ ^127^ ^]^ Meanwhile, the magnetization hysteresis loop also indicated the magnetic property of Cu_x_Co_y_S NPs. The near‐infrared (NIR) light absorption ability and magnetization endowed Cu_x_Co_y_S NPs with coordinate effects for reducing senescent cells in vitro and in vivo. At the same time, Pen as ligands mediated the fabrication of 75.0 ± 8.0 nm Cu_x_Co_y_S NPs. Cu_x_Co_y_S NPs showed broad CD bands from 400 nm to 900 nm, which could induce accelerated generation of ROS by irradiation of 808 nm light.^[^ ^128^ ^]^ Chiral Fe_x_Cu_y_Se NPs were also designed in the presence of Pen enantiomers,^[^ ^123^ ^]^ and showed CD peaks with opposite in sign from 190 nm and 1000 nm, in which the characteristic peaks occurred at 262, 312, 350, 440, 510, and 750 nm. Furthermore, Pen enantiomers as chiral ligands were used for the construction of chiral Cu_2‐x_S NPs. Chiral Cu_2‐x_S NPs showed CD responses at 201 nm, 270 nm, 608 nm, and 863 nm owing to the interaction between Pen enantiomers. The chiroptical activity of Cu_2‐x_S NPs originated from the centrosymmetric shapes of NPs and the dissymmetric field induced by the Coulomb interactions of electrons and holes. The chirality could be enhanced by adjusting the concentration of Phe enantiomers, which showed positive relationships with g‐factor reached up to 1x10^−2^. The enhanced chiroptical activity enabled chiral Cu_2‐x_S NPs to serve as photocatalysts for the application of protein catalysis and profiling under circularly polarized light irradiation. The chiral molecule enantiomers were widely applied as chiral selectors and were functionalized on the surface of these metal sulfide NPs.

The supraparticles demonstrated collective or synergistic properties that provide great potential for catalysis application.^[^ ^129^ ^]^ Pen enantiomers driven chiral ZnS NPs and the corresponding supraparticles were fabricated. In particular, 3.0 ± 0.7 nm chiral Pen‐ZnS NPs were obtained and displayed a new chiroptical signal, attributing to the electronic hybridization between the molecular orbitals of NPs and those of Pen, as well as the local distortions of Pen‐modified ZnS surface. Pen‐ZnS NPs were aggregated into supraparticles with the sizes of 100.0 ± 3.0 nm, and displayed new chiroptical peaks from 280 nm to 340 nm, benefiting from the energies of the electronic state from the amino acid residues modified NP surface. In comparison to Pen‐ZnS NPs, Pen‐ZnS supraparticles showed better enantioselective recognition and enhanced photocatalytic activity for tyrosine (Tyr) enantiomers.^[^ ^130^ ^]^ The introduction of Au into Pen‐ZnS‐Au supraparticles promoted the generation of hot electrons and holes and also decreased the band gap of ZnS, which further enhanced the photocatalytic conversion efficiency of Tyr. Based on Cys enantiomers functionalized chiral ZnS, chiral ZnS supraparticles were assembled by achiral citrate‐stabilized ZnS nanoshells in the presence of Cys enantiomers.^[^ ^49^ ^]^ The replacement of citrate by Cys enantiomers induced the assembly of ZnS nanoshells into ZnS supraparticles. Chiral ZnS supraparticles were also obtained by direct assembling of Cys enantiomers modified ZnS NPs. The Cys functionalized chiral ZnS supraparticles exhibited intense CD peaks in the regions of 265 nm‐400 nm, originating from the quantum mechanical coupling between chiral surface electronic states and lattice distortions. Chiral ZnS supraparticles displayed potential photocatalytic ability for the enantioselective oxidization of Tyr under light illumination.

**Figure 2:**
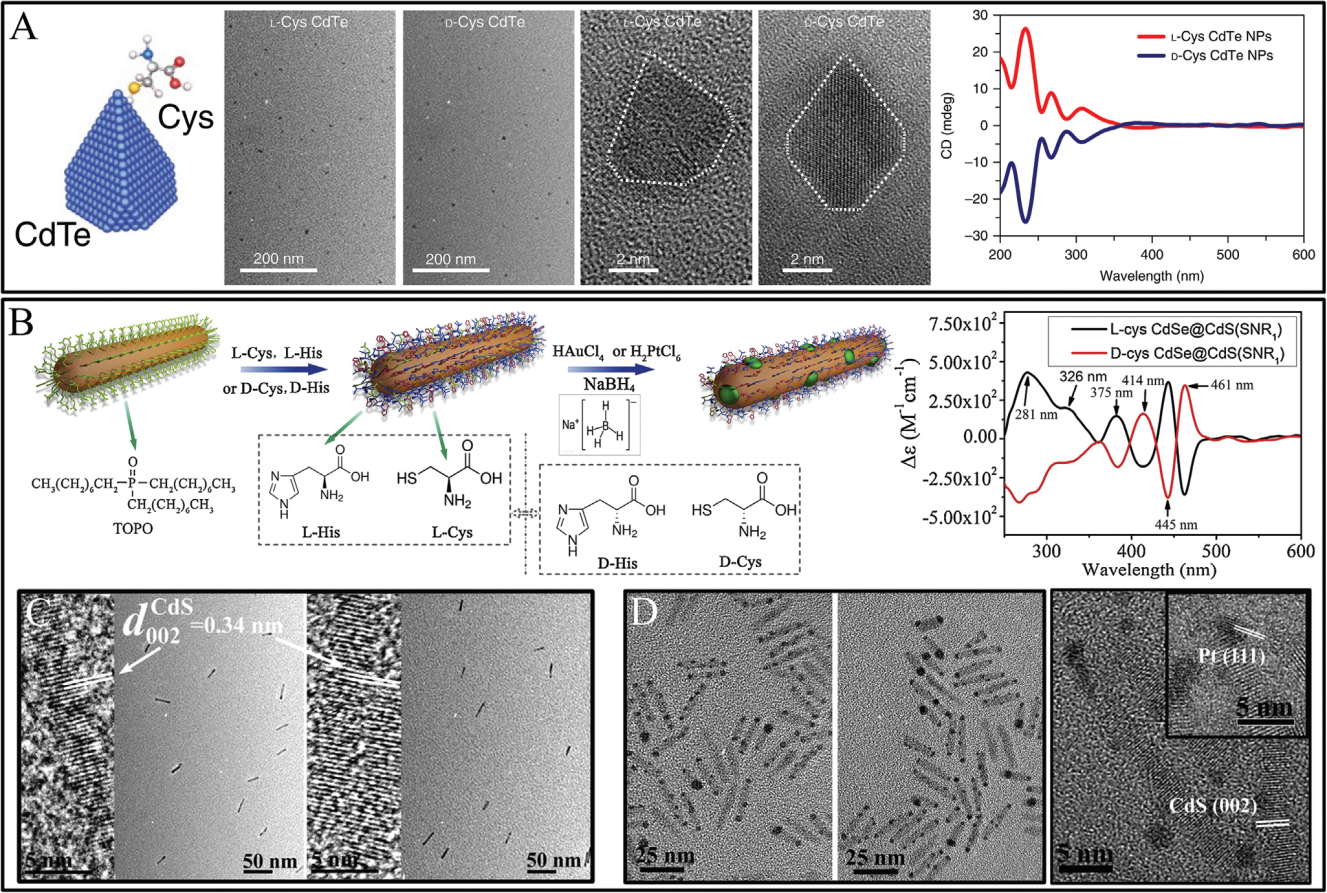
A) Schematic illustration of chiral CdTe nanoparticle, TEM, and high‐resolution TEM images of truncated tetrahedron‐shaped L‐Cys and D‐Cys CdTe nanoparticles, and their corresponding CD spectra.^[^ ^116^ ^]^ B) Schematic illustration of chiral semiconductor‐metal hetero‐nanorod and chiroptical properties.^[^ ^124^ ^]^ C,D) TEM images of D‐cys‐, or L‐cys‐CdSe@CdS nanorods C), and TEM of the D‐cys, D‐his CdSe@CdS‐Pt hetero‐nanorods (left) and L‐cys, L‐cys CdSe@CdS‐Pt hetero‐nanorods and the high‐resolution images D).

In order to obtain strong chiral semiconductors, different ligands were applied for the construction of chiral semiconductors. Chiral CdTe NPs were attempted in the presence of N‐acetyl‐l‐Cys, N‐isobutyryl‐l‐Cys, dipeptide (Cys‐Phe), Cys, achiral 2‐mer‐capto acetic acid, GSH, and l‐Cys methyl ester hydrochloride (**Figure** **2**A).^[^ ^116^ ^]^ It was found that prominent enhanced CD peaks occurred for chiral CdTe NPs functionalized Cys enantiomers. Interestingly, the truncated tetrahedral shapes of CdTe NPs induced the specific recognition of the structures of GAT′ATC of DNA chains, achieving the site‐selective DNA cleavage under the illumination of circularly polarized light. Alternatively, various amino acids were attempted to stabilize chiral Cu_2_S NPs. The Cu_2_S NPs with 4.0 ± 0.5 nm were obtained by using peptide penicillamine‐phenylalanine‐tryptophan (Pen‐Phe‐Trp) as ligands and showed characteristic CD signals ta 530 nm with the intensity of 46 mdeg.^[^ ^131^ ^]^ Based on sonication treatment with NaOH in N‐methyl‐2‐pyrrolidone solution, a series of 2D transition metal dichalcogenides, such as MoS_2_ and WS_2_ quantum dots (QDs), were synthesized and exhibited CD signals after modification of Cys and Pen,^[^ ^48^ ^]^ owing to the dipolar interaction between the excitations of QDs and of ligand molecules. Benefiting from the binding of chiral QDs toward Tyr, chiral QDs exhibited well peroxidation‐like behaviors for the enantioselective catalytic oxidation of Tyr. Based on the phase transfer mediated by Cys and histidine (His) and the post‐growth of a metal (Au, Pt), the optical activity of SNR heterostructures was strongly enhanced (g‐factor: 2.2*10^−3^) (Figure 2B–D).^[^ ^124^ ^]^ The functionalization of chiral ligands not only enabled semiconductors to exhibit chiral behaviors in the ultraviolet (UV)‐visible regions but also endowed semiconductors with unique enantioselectivity during photocatalysis. The application of QDs as photocatalysis is an emerging field for catalysis. QDs with narrow bandgap could efficiently utilize visible light for photocatalytic applications. The relationship between the characteristics of bandgap, hot carrier separation, and chiral responses as well as chiral catalytic behaviors should be exploited in detail. The large‐scale production of chiral semiconductors with tailored chiral geometry remains a central challenge.

### 2.4 Chiral Carbon Materials

Even though many more studies have been reported for the fabrication of chiral carbon nanomaterials, such as chiral carbon dots (CDs), carbon nanotubes (CNTs), and graphene oxide (GO) that also possess good functions for biocatalytic applications.^[^ ^132^, ^133^, ^134^ ^]^ The carbon materials displayed excellent properties such as biocompatibility, low toxicity, and facile synthesis and modification that make them strong competitors to traditional materials.^[^ ^135^, ^136^, ^137^ ^]^ The emergency need for system evaluation of carbon nanostructures for understanding different manifestations of chirality would be essential for many fields of science.^[^ ^138^, ^139^ ^]^

**Figure 3:**
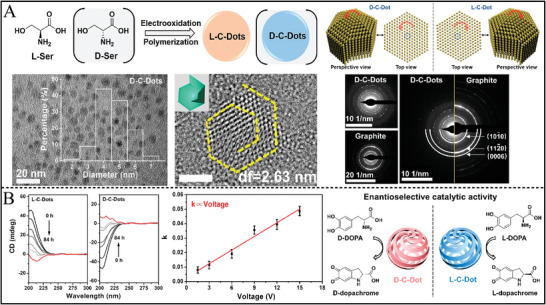
A) Schematic for the synthesis of chiral carbon dots and the TEM, high‐resolution TEM, and selected area electron diffraction pattern results. B) CD spectra and voltage‐dependent growth kinetics, and enantioselective catalytic activity of chiral carbon dots for DOPA enantiomers oxidation.^[^ ^141^ ^]^

The synthesis of CDs can be from different carbon sources, in which chiral structure control remains challenging and unclear.^[^ ^137^ ^]^ For example, chiral CDs are fabricated by thermal polymerization of chiral amino acids and citric acid with the handedness of chirality controlled by chiral source and reaction temperature.^[^ ^140^ ^]^ With aliphatic amino acids as a chiral source, the CDs obtained at temperatures (90−200 °C) have the same handedness, while with aromatic amino acids as a chiral source, CDs displayed inversed handedness. Below a temperature of 120 °C, CDs were modified with chiral amino acid by esterification and transferred the ligand chirality; at high temperatures (above 150 °C), the formation of the rigid structure generated by the π conjugation between the aromatic nucleus of chiral source and the carbon core of CDs caused the inversing of the chiral signal. Alternatively, based on citric acid and D‐Pro as precursors, chiral CDs were fabricated by one‐step hydrothermal reaction at 180 °C for 2 h, which shows high asymmetric catalytic activity for enantioselective direct aldol condensation.^[^ ^46^ ^]^ The hydrothermal synthesis of chiral CDs provides a simple method for heterogeneous chiral catalysts. Additionally, for chiral CDs synthesis, a clear structure−property was important for enantioselective catalysis. The CDs fabricated by electrooxidation polymerization from serine (Ser) enantiomers at room temperature displayed clear structure characteristics (**Figure** **3**A), in which the L‐Ser or D‐Ser was polymerized at a voltage of 3.00 V for 72 h.^[^ ^141^ ^]^ The electrooxidation of the hydroxyl of L‐Ser or D‐Ser was a rate‐determining step during L‐CDs or D‐CDs fabrication and made the CDs display inverse chiral handedness (Figure 3B). The chiral CDs had uniform size of 2−7 nm and exhibited a well‐defined primary structure of polycyclic dipeptide and spatial structure with a c‐axis of hexagonal symmetry and two cyclic dipeptides. These chiral CDs endowed enantioselective oxidation on 3,4‐dihydroxy‐phenylalanine (DOPA) enantiomers. Alternatively, the chiral CDs can be synthesized from one‐step base‐catalyzed aldol condensation from glucose at ambient temperature, which includes the isomerization and aldol condensation.^[^ ^39^ ^]^ These chiral CDs also displayed a selective capacity for electrocatalytic oxidization of Trp enantiomers. The chiral nanoporous carbons were recently synthesized based on chiral amino acids ionic liquids as precursors and displayed high enantioselectivity.^[^ ^142^ ^]^ The CD bands for CDs mostly in the wavelength range of 200–300 nm, majority of the bands are located at 200–250 nm.^[^ ^39^, ^140^ ^]^ The chiroptical activity originated from the surface bearing of chiral molecules such as amino acids.^[^ ^46^, ^141^ ^]^

The synthesis and separation of CNTs with chiral conformation of graphene sheets is possible and symmetry‐matched nanotubes with opposite rotatory optical activity have recently become available.^[^ ^143^, ^144^ ^]^ Among such π‐conjugated materials, single‐walled or multiwalled CNTs have attracted wide attention that act as ideal solid support for molecules,^[^ ^38^ ^]^ nanomaterials^[^ ^145^ ^]^ through physical adsorption, adhesion, and chemical modifications.^[^ ^146^, ^147^ ^]^ For example, the D‐4‐amino‐Phe was modified to CNTs surface by diazo‐reaction through refluxed at 80 °C for 15 h in water. The chiral molecule‐modified CNTs allowed a metal‐free cathode, effectively induced the asymmetric reduction of a wide range of substrates, and can thus be used instead of noble metal catalysts.^[^ ^38^ ^]^ Alternatively, the Keggin‐type polyoxometalate cluster was high efficiently grown inside CNTs by stirring precursor and CNTs for 96 h at room temperature.^[^ ^145^ ^]^ The chemical reduction synthesis and electrodeposition methods have been used for the construction of NPs decorated CNTs.^[^ ^148^, ^149^, ^150^ ^]^ Furtherly, the ultrathin (0.8−2.0 nm) W_2_C and Mo_2_C nanowires were synthesized confining in CNTs deriving from the encapsulated W/Mo polyoxometalate clusters. The Pd NPs were then grown on CNTs through a thermal reduction method that generated the selective semihydrogenation of alkynes. CD spectra of CNTs with *M*‐(6,4) were experimentally found muti‐bands in the spectra region of 250–800 nm.^[^ ^151^ ^]^ The gown helical CNTs usually displayed typical nanoscale chiral structures morphology that induced chiroptical activity at wide spectra region (300‐800 nm).^[^ ^152^ ^]^ The constructed hierarchical superstructure helps to achieve intensive CD signals by synergistically enhanced effect.

The graphene synthesis required a temperature in the range of several hundred degrees on the Celsius scale. The graphene nanoribbons have atomic and electronic structures that obtained wide interest.^[^ ^153^ ^]^ For example, the heteroatom‐doped chiral (4,1)‐graphene nanoribbons were fabricated by surface‐assisted synthesis from precursor 6,16‐dibromo‐9,10,19,20‐tetraoxa9a,19a‐diboratetrabenzo[*a,f,j,o*]perylene with a temperature range of 80–450 °C.^[^ ^154^ ^]^ Similarly, the thermostable 4.3 nm helical graphene nanoribbon was synthesized with components of four linearly fused superbenzenes with an end‐to‐end twist of 117°.^[^ ^155^ ^]^ Post modification was also popular for introducing chirality to graphene, for example, the L‐(or D‐)Cys with amine group was modified on graphene QDs by their carboxylic group through 1‐(3‐Dimethylaminopropyl)−3‐ethylcarbodiimide/N‐Hydroxysuccinimide method,^[^ ^138^ ^]^ which is similar for chiral molecules modification of GO.^[^ ^156^ ^]^ GO and its derivatives were deemed as important carbon materials and obtained considerable attention,^[^ ^136^ ^]^ in which GO could be fabricated by oxidation of natural graphite powder. The covalent attachment of L/D‐cysteine moieties to the edge of graphene CDs resulted in helical buckling due to chiral interactions.^[^ ^138^ ^]^ Thus, despite chiral bands at 210−250, they also displayed rich bands at 250−265 nm with a typical Cotton effect.^[^ ^138^, ^157^ ^]^ When further twisted into helical graphene nanoribbon, the graphene twistacenes exhibited rich chiroptical bands at a longer wavelength range from 250–650 nm,^[^ ^155^ ^]^ which originated from intensive structure chirality.

### 2.5 Chiral Framework Materials

Typically, organic frameworks included metal‐organic frameworks (MOF), covalent‐organic frameworks (COF), hydrogen‐bonded organic frameworks, and molecular cages^[^ ^158^ ^]^ featuring periodic, unambiguous structures that were built from organic linkers with active groups, chiral molecules, and unsaturated metal ions/clusters, and showed enhanced catalytic activity.^[^ ^159^, ^160^ ^]^ The unique porous structures, tunable pore sizes, and high surface‐to‐volume ratio enabled chiral MOF and COF to absorb and enrich more subtracts in the application of heterogeneous catalysis and photocatalysis.^[^ ^159^, ^161^ ^]^ Organic frameworks have much more diversified structures with highly tunable pore sizes (usually 0–3 nm, up to 9.8 nm),^[^ ^162^ ^]^ in which many frameworks possessing semiconductor‐like behavior enabled great potential in asymmetric catalysis.

To fabricate chiral MOF, it was usually the incorporation of chiral molecules in space groups or the chiral arrangement of the components in the framework.^[^ ^158^, ^163^, ^164^ ^]^ Generally, a chiral framework could be fabricated by three methods: (1) The direct method: the chiral linkers or colinkers (auxiliaries) used to introduce chirality transferred from chiral molecules to the framework; (2) The post‐synthetic modification method: based on functional groups, the achiral framework modified with chiral molecules to implant chirality to framework; (3) The spontaneous resolution and chiral induction method: the achiral components organized in a chiral form, for example, helices to generate chiral geometrized framework.

The direct synthesis method is popularly preferred for synthesis of chiral MOF which was due to “chirality conservation” control to achieve homochirality.^[^ ^165^ ^]^ Based on photosensitive porphyrin, chiral propargylamine linkage, and amphipathic quaternary ammonium bromide, the phenylacetylene‐derived COF was synthesized via asymmetric A^3^‐coupling polymerization under ambient conditions.^[^ ^166^ ^]^ Such propargylamine‐linked and quaternary ammonium bromide decorated porphyrin‐COF displayed enhanced visible‐light‐driven enantioselective photooxidation of sulfides to sulfoxides in water. The chiroptical bands usually have spectra region of 200–350 nm with a typical Cotton effect.

For the post‐synthetic modification method, the parent framework should possess properties of chemically stable and large internal surfaces with potentially functional groups and may suffer from inadequate modification. Chiral molecules modified framework was able to have enantioselective recognition to chiral subtract or biomolecules.^[^ ^167^ ^]^ For example, the chiral β‐cyclodextrin (β‐CD) post‐modified to COF via thiol−ene click reactions enabled selective recognition of amino acids.^[^ ^168^ ^]^ Different amino‐acid (e.g., valine, serine, and threonine) had been reported to elaborate isoreticular MOF that displayed single‐chiral‐site precision for asymmetric hydrogenation.^[^ ^169^ ^]^ Alternatively, the L‐His was modified to COF backbone, which is constituted with 5,10,15,20‐tetrakis(40‐tetraphenylamino) porphyrin unit as the active center.^[^ ^170^ ^]^ The amino acid usually introduces the substrate binding site for chiral recognition. Furthermore, metals such as Au, Pd, etc., also could be functionalized to framework to introduce enhance asymmetric catalysis activity.^[^ ^171^, ^172^ ^]^ Sometimes, the chiral catalyst could be encoded within the framework by asymmetric polymerization with the amine‐ or hydrazine‐monomers, and chirality was induced at the same time.^[^ ^173^, ^174^ ^]^ Additionally, the interface growth of a chiral framework was also possible, for example, the chiral Salen(Mn(III))@Zeolitic Imidazolate Framework‐8 nanostructures were grown on polystyrene nanospheres with encoded (R,R)‐Salen(Mn(III)) catalyst in the cavity.^[^ ^175^ ^]^ The post‐synthetic modification fabricated MOF introduced mirror‐symmetric CD bands in the spectra range of 200–250 nm with large similarity of chiral amino acids.^[^ ^169^ ^]^ The metal atoms, such as Au‐incorporated COF materials can help to extend chiroptical activity to visible wavelength regions (200–800 nm).^[^ ^173^ ^]^

The spontaneous resolution and chiral induction methods were newly developed methods which usually the synthesis of chiral framework through a symmetry‐breaking process, in which a large quantity of chiral framework was fabricated by this method.^[^ ^176^, ^177^, ^178^ ^]^ For example, the gamma‐cyclodextrin (γ‐CD)‐based metal−organic framework contained body‐centered frameworks containing (γ‐CD)_6_ cubes integrated by cations, cylindrical (γ‐CD)_2_ tunnels, and triangular channels between 1.7 nm spherical pores that induced the porous helical structure of MOF.^[^ ^176^ ^]^ The CD bands appeared in a spectra range of 400–600 nm with a wide peak. The helical framework can also synthesis on substrate, for example, the helical Cu‐porphyrinic MOF Cu meso‐tetra(4‐carboxyphenyl)porphyrin was controlled growth on Cu(OH)_2_ nanoarrays using a sacrificial template method, and showed enhanced catalysis activity for CO_2_ electroreduction.^[^ ^178^ ^]^ The fabrication of chiral porous MOF films could be a candidate strategy for enhanced chirality.^[^ ^179^ ^]^ By including lanthanide complexes in the framework, the chiral MOF films displayed CD and circularly polarized luminescence at spectra region of 350–750 nm. The COF in the form of a membrane could also achieve high catalytical performance with easy recovery.^[^ ^180^ ^]^ The major challenge for the spontaneous resolution and chiral induction method was that both enantiomers form with equal probability, and may obtain racemic conglomerates during the symmetry‐breaking process.

### 2.6 Chiral Assembled Nanostructures

In order to obtain intensive chiroptical responses, biomolecules (DNA, amino acids, peptides) engineered NPs assemblies (involving dimers, trimers, pyramids, chains, helices, and so on) were also widely prepared and displayed enhanced chiral responses, owing to the formed unique chiral configuration.^[^ ^181^ ^]^ One of the advantages of NP assemblies was the generation of controllable and amplified chiral signals when the single building blocks did not possess chiroptical activity.

The most straightforward scissor‐like geometry of NP dimers also showed chiral responses benefiting from the formed dihedral angle among two NPs. Chiral dimers, such as Au NP dimers, Au NR dimers, and Au NP‐Ag NP dimers, were widely assembled by using DNA, amino acids, peptides, antibody–antigen, etc.^[^ ^182^, ^183^ ^]^ A dihedral angle within 90° was formed between NPs. Au NP‐Ag NP heterodimers displayed an angle of ≈9° by using antibody‐antigen as bridges.^[^ ^182^ ^]^ Au NR dimers had a 7–9° twist between the long axes of NRs.^[^ ^183^ ^]^ The chiral peak position and the intensity of chiral signals were determined by the NP components and morphology.^[^ ^184^ ^]^ In order to further adjust the position and intensity of CD peaks, the deposition of one or two layers of metal shell achieved the blue and red shifts of chiral signals, as well as a significant amplification of CD signals. For example, Au NP dimers displayed a CD peak at 525 nm, which can be blue‐shifted to 418 nm after the reduction of Ag coating and red‐shifted to 586 nm after the growth of Au coating.^[^ ^185^ ^]^ The coating of the metal shell achieved the adjustment of chiroptical responses for the sensitive bioapplications.

Chiral assembled structures possessed pronounced chiral configuration and pre‐designed components for catalysis. For example, DNA‐driven shell–satellite gold assemblies as chiral photosensitizers displayed high ROS generating efficiency under light illumination, resulting in a 1O^2^ quantum yield of 1.09. Meanwhile and exhibited a remarkable photodynamic therapy effect.^[^ ^186^ ^]^ Most studies were limited to fabricating chiral nanomaterials in the liquid solution. To overcome this intrinsic limitation, solid‐like chiral films were crucial for the fabrication of optical nanomaterials in practical applications.^[^ ^187^ ^]^ For example, chiral Au NP films were designed by applying Phe enantiomers‐modified Au NPs in the hexane/water interface.^[^ ^188^ ^]^ Chiral Au NPs film showed intense chiral intensity at 520 nm and 732 nm, in which mirror chiral peaks were observed between D‐Phe and L‐Phe modified Au NP films, in which the CD intensity enhanced with the increasing amounts of Phe enantiomers. The monolayer chiral Au NP films featured with chiral enantiomers exhibited mimic enzyme properties for the selective oxidation of glucose enantiomers. However, there is still a challenge to fabricate chiral nanomaterials for the actual applications in a matter form.

### 2.7 Chiral Assembled Supramolecular Architectures

The three‐dimensional chiral assembly of supramolecular nanoarchitectures could also provide for enantioselective recognition and enhanced biocatalysis.^[^ ^189^ ^]^ Chiral‐assembled supramolecules are composed of multiple building blocks, which may all be chiral or only one of them.^[^ ^190^, ^191^, ^192^ ^]^ Biomolecules and their derivatives could be well assembled into chiral superstructures that can mimic complex biosystems for better comprehending the origin of natural chirality.^[^ ^193^, ^194^ ^]^ Amino acids can be self‐assembled into helical structures by modifying fused aryl groups at the N‐terminal. Based on duplex H‐bonds and charge‐transfer interactions orthogonally, melamine induces the self‐assembly of amino acids into giant tubular structures at the microscale via helical scrolling with a g‐factor of 1.4 × 10^−2^.^[^ ^195^ ^]^ A chiral bolaamphiphile terminated with L‐glutamic acid was used to self‐assemble single‐walled nanotube chiral supramolecules. Transition metal ions were coordinated with the nanotube surface carboxylic acid groups to fabricate various single‐walled chiral supramolecules.^[^ ^196^ ^]^ Amino acid methyl esters were conjugated to anthracene segments, leading to helical structures in solid states with different modes for different residues.^[^ ^197^ ^]^ The ternary coassembly produces super‐helical structures conjugated with chiral amino acids and fused rings via multiple hydrogen bonding and charge transfer interactions, exhibiting topological evolution from ribbon to helix, spring, and closed toroid.^[^ ^198^ ^]^ For example. The achiral polymer and two gelators bearing D‐ or L‐ Phe as chiral centers successfully constructed 3D chiral supramolecular and integrated lipase for enhancing the catalysis activity.^[^ ^199^ ^]^ The chirality of programmed supramolecular showed intensive CD signals at 200–450 nm wavelength range which was transferred from molecular chirality of D‐ or L‐ Phe chiral center. It was reported that amphiphilic L‐Pro−L‐ Glu and L‐Pro−D‐Glu dipeptides were assembled into synergistic supramolecular nanofiber for asymmetric catalysis by two stereogenic centers.^[^ ^200^ ^]^ Chiral self‐assembly of biomolecules with related polymer nanostructures has drawn tremendous interest in recent years and will continuously be hot in the future.^[^ ^201^ ^]^

The facile design of molecular structures can help to induce twisted supramolecular structures. Such supramolecular chiral polycyclic aromatic hydrocarbon architectures with π‐subplanes were achieved through a slip‐stacked chiral arrangement by kinetic self‐assembly in low‐polarity solvents. Phenyl substituents provided stable axial chirality that showed a sharp optical signature of J‐type excitonic coupling in both absorption (897 nm) and emission (912 nm), with an absorption g‐factor of up to 1.1 × 10^−2^.^[^ ^192^ ^]^ Similarly, incorporating a helical polymer with a strong tendency toward self‐assembly into a π‐conjugated polymer results in a precise and asymmetric self‐assembly of π‐conjugated polymers. The chirality transfer from the helical polymer block to the π‐conjugated polymer creates chiral supramolecular architectures with unique chiroptical properties, induced white light emission of a broad optical spectrum and CPL.^[^ ^191^ ^]^

Alternatively, chiral supramolecules can be constructed by hybridization of chiral and achiral substances. The Bis‐hydrazone pyridinium conjugates were modified with chiral alkyl chains, resulting in a self‐assembly that preferentially occurred in a particular direction directed by hydrazone building blocks,^[^ ^202^ ^]^ which comprised water through hydrogen bonding interactions, directed by structural water molecules. The generated CPL exhibited a maximum g_lum_ of 2.6 × 10^−2^. The study on supramolecular polymerization provided insight into hydrazone derivative self‐assembly. Chiral copolymeric guest molecules self‐assemble into nanohelixes based on pillar[5]arene host with g‐factor (g_lum_ = 1.32 × 10^−2^),^[^ ^203^ ^]^ which was stronger than O_h_‐symmetric hydrogen‐bonded resorcin[4]arene capsule with C2‐symmetric cationic bis‐cyclometalated Ir assembly complexes (glum = 8 × 10^−4^)^[^ ^204^ ^]^ and amphiphilic poly(3‐hexylthiophene)‐block‐poly(phenyl isocyanide) copolymers helical assemblies (glum = 3.6 × 10^−3^).^[^ ^205^ ^]^

The chiral assembled metal‐organic complexes supramolecular material can be created through coordination‐driven force.^[^ ^206^, ^207^ ^]^ This chiral environment enables them to interact very specifically with one enantiomer over the other, resulting in exceptional enantiorecognition properties.^[^ ^207^ ^]^ Additionally, the self‐assembly of two or more building blocks with distinct functions into nanostructures has the ability to enhance the chiroptical effect.^[^ ^208^ ^]^ After incorporating a chiral Rh complex into a nonchiral supramolecular cage formed through coordination‐driven assembly of macrocyclic dipalladium and tetracarboxylate zinc porphyrins, the resulting catalyst exhibits high regio‐ and enantioselectivity during the hydroformylation of styrenes.^[^ ^209^ ^]^ Additionally, supramolecular gels can be assembled through coordinating metal ions, such as cholesterol‐azopyridine conjugate with metal ions self‐assembly organogels exhibit controllable supramolecular chirality, demonstrating metal ion‐mediated and solvent‐dependent chirality inversion.^[^ ^210^ ^]^ Efficient metal coordination‐driven self‐assembly of chiral 3,3′‐dipyridyl substituted BINOL donor constructs chiral metallic triangles.^[^ ^211^ ^]^ The catalytic centers in a confined space of the cavity tightly surrounded by chiral building blocks can help to enhance catalytic activity stereoselectivity.

Future new developments in assembly strategy may help to produce a variety of chiral supramolecular architectures. Using the evaporation‐induced self‐assembly technique, synthesize supramolecular glasses by employing Zn‐L‐Histidine complexes. The metal‐ligand interactions between the zinc(II) ion and chiral L‐Histidine induced the assembly of supramolecules and gave an interesting multicolored CPL from blue to red with a g_lum_ factor of 9.5 × 10^−3^.^[^ ^212^ ^]^ External force (rotating and gravitational force, magnetic force, etc) can induce chirality in supramolecular assemblies that are not initially chiral.^[^ ^194^, ^213^ ^]^ For example, it was found that creating laminar chiral microvortices in asymmetric microchambers can quickly lead to the initial chiral bias of achiral supramolecular systems.^[^ ^213^ ^]^ Similarly, the magnetic field correlated with gravitational force could induce tris‐(4‐sulfonatophenyl)phenylporphyrin (TPPS_3_) aggregated into helical structures, in which chirality is determined by the relative directions of rotation, effective gravity, and essential magnetic field orientation. Parallel arrangement of angular momentum and effective gravity resulted in negative CD signals while antiparallel arrangement of angular momentum and effective gravity induced positive CD signals.^[^ ^194^ ^]^ The external force‐driven chiral assembly may help to reveal the origin of homochirality in life as a natural homochirality process occurred in the existing Erath self‐rotating, gravitational force.

## 3 Selective Catalysis Activity of Chiral Nanomaterials

### 3.1 Chiral Metal NPs‐Based Catalysis

Various artificial chiral nanozymes had been fabricated with chiral ligands such as amino acids for the enantioselective catalysis applications (**Table** **1**).^[^ ^214^ ^]^ The development of chiral nanomaterials with intense chiral activity, high selectivity, and well stereocontrol was still a challenge for enantioselective catalysis. In order to increase the chiral recognition, amino acids served as chiral selectors were coated on an inorganic metal NPs surface.^[^ ^215^ ^]^ Inorganic metal NPs, such as Au NPs and Ce NPs, were prepared for the enantioselective catalysis oxidation of chiral DOPA.^[^ ^216^, ^217^ ^]^ Cys functionalized Au NPs were easily fabricated through the covalent bond between Au NPs and sulfhydryl group from Cys. In particular, D‐cys functionalized Au NPs were prone to oxidize L‐DOPA, and L‐cys functionalized Au NPs were prone to oxidize D‐DOPA.^[^ ^217^ ^]^ This was due to the high association between D‐DOPA and D‐cys, and the binding force between L‐DOPA and L‐cys through hydrogen bonding interaction, which inhibited the oxidation of DOPA. The work inspired us to explore a series of amino acid‐modified artificial enzymes with stereo‐selective enzyme mimics. Recently, eight amino acids, involving Trp, Glu, alanine, Phe, arginine, His, Tyr, and lysine, were designed to modify Ce NPs.^[^ ^216^ ^]^ The stereoselectivity of different amino acids modified Ce NPs were attempted to explore for the application of enantioselective catalysis oxidation of chiral DOPA, owing to the multienzyme mimetic activity of catalase, oxidase, and superoxide oxidase as well as the existence of oxygen vacancies of Ce NPs. It was found that L‐Phe functionalized Ce NPs were prone to oxidizing D‐DOPA, and D‐Phe functionalized Au NPs were prone to oxidizing L‐DOPA. This was attributed to the hydrogen bonding and π‐π aromatic packing interaction among Phe enantiomers and DOPA enantiomers.^[^ ^216^ ^]^ Similarly, the chiral amino alcohol ligands modified silver nanoclusters were prepared for non‐homogeneous asymmetric catalysis, which achieved higher ee (70%) than the homogeneous phase (63%).^[^ ^214^ ^]^ Inorganic metal NPs after the grafting of chiral selectors had become the candidate catalysis for the applications of heterogeneous enantioselective catalysis. This principle could be extended to the construction of other chiral nanomaterials by applying amino acids as chiral ligands.

**Table 1:** Catalysis performance of representative chiral nanomaterials.

Chiral nanomaterials	Catalyst compositions	Chiral ligands or moieties	Catalysis reactions	Substrates	Enantioselectivity	Yield (%)	Reference
Metal NPs	Pd NPs	Cinchonidine, S‐proline	Asymmetric hydrogenation	(E)‐α‐phenylcinnamic acid	34 (ee, %)	49.8	[44]
	Ir NPs	4,5 Dihydro‐3H‐dinaphthoĳ2,1‐c:1′,2′‐e] phosphepine‐4‐oxide	Asymmetric hydrogenation	Prochiral ketones	55 (ee, %)	88	[45]
	GO supported Ni NPs	D‐or L‐tartaric acid	Asymmetric hydrogenation	Methyl acetoacetate	98.6 (ee, %)	99	[219]
	Pt‐Ir alloys on nickel foams	Chiral phenylethanol	Asymmetric hydrogenation	aromatic ketones	>80% (ee, %)	/	[220]
Metal oxide NPs	Fe_3_O_4_@MWCNTs	Reductase KmAKR	Asymmetric hydrogenation	Aliphatic ketones	99.5 (ee, %)	99	[223]
	(L‐proline@Fe_3_O_4_)@[Zn(OBA)(BPDH)0.5]n	Proline	Asymmetric Henry condensation	β‐nitroalcohol, benzaldehyde	98 (ee, %)	100	[256]
	Al_2_O_3_, SiO_2_, SiO_2_–TiO_2_, Al_2_O_3_–TiO_2_	N‐[(2S,4R)−4‐hydroxyprolyl]‐(S)1‐phenylethylamine	Biginelli reaction	Urea, benzaldehyde, ethyl acetoacetate	66 (ee, %)	92	[221]
Semiconductor NPs	MoS_2_, WS_2_ QDs	D‐ or L‐Cys	Oxidation	D‐ or L‐Tyr	Enantioselectivity ratio: 6.77	/	[48]
	ZnS supraparticles	Tyrosine	Oxidation	DOPA, Tyr−Tyr	23−26 (ee,%)	/	[49]
Carbon materials	CDs	Proline	Asymmetric aldol condensation	p‐nitrobenzaldehyde, cyclohexanone	73 (ee, %)	98	[46]
	GO	Proline	Asymmetric aldol addition	2‐nitrobenzaldehyde, acetone	77 (ee, %)	85	[230]
	CNTs	Chiral 2,2′‐bipyridine ligand	Asymmetric conjugate addition	Benzaldoxime, non3‐en‐2‐one	95 (ee, %)	79	[146]
	MWCNTs	Phenylalanine	Asymmetric Electroreduction of Aromatic Ketones	2,2,2‐trifluoroacetophenone	30 (ee, %)	64	[38]
Framework materials	MIL‐101‐PP1	L‐proline	Asymmetric Aldol reaction	p‐nitrobenzaldehyde, cyclohexanone	96 (ee, %)	89	[47]
	Phenylacetylene derived CCOF,	Chiral propargylamine	Enantioselective Photooxidation	Sulfides	99 (ee, %)	94	[166]
	(S)‐DTP‐COF	Chiral propargylamine	Asymmetric Michael addition reactions	Cyclohexanone, 1‐((E)−2nitrovinyl)benzene	96 (ee, %)	97	[244]
	1,3,5‐tris(4‐aminophenyl) benzene and Dimethoxyterephthalaldehyde COF	L‐proline‐ and L‐imidazolidine	Asymmetric α‐Αminooxylation of Aldehydes	Aldehyde, nitrosobenzene/ketone/cyclopentadiene	96 (ee, %)	98	[243]
Assembled nanostructures	Polymers‐based chiral Ru‐Cu assembly	Chiral b‐CD‐g‐(PTsDPEN‐b‐PNIPAAm)copolymer	Asymmetric transfer hydrogenation	Ketone, acetophenone	98.1 (ee, %)	98.5	[85]
	L‐Pro−L‐Glu dipeptides assembly	L‐Pro−L‐Glu dipeptides	Asymmetric aldol reactions	Aldehyde, cyclohexanone	99 (ee, %)	97	[200]
	Polymeric NPs	Chiral salen Fe^III^ monomers	Asymmetric sulfa‐Michael addition	Thiols, enones or chalcone	93−99 (ee, %)	90−98	[254]
	Supramolecule	Chiral macrocyclic multifarane[3,3]	Asymmetric hydrogenation	4‐methylumbelliferone	>99 (ee, %)	65	[250]

Alternatively, using the selective adsorption of chiral Cys on the high Miller index surface of gold NPs, vein‐like gold NPs were prepared with chiral plasmonic response and good enantioselectivity for the electrocatalytic activity of Trp oxidation.^[^ ^218^ ^]^ When L‐Cys‐Au NPs were used as catalysts, the oxidation of D‐Trp produced a higher peak current (29.12 µA) and a lower peak potential (0.716 V) compared with L‐Trp (14.87 µA, 0.736 V). A series of experimental results showed that this pulsed gold oxide has good enantioselectivity for the electrocatalytic oxidation of Trp enantiomers. Chiral cinchonidine or S‐proline was used for one‐pot synthesis of dendritic or cubic Pd NPs by replacing the conventional capping agents.^[^ ^44^ ^]^ The fabricated chiral Pd catalyst exhibited asymmetric hydrogenation catalysis activity against (E)‐α‐phenylcinnamic acid with moderate ee as 34% and a yield of 49.8%. The method introduced heterogeneous enantioselective hydrogenations with facile chiral modification step of metal catalysts.

**Figure 4:**
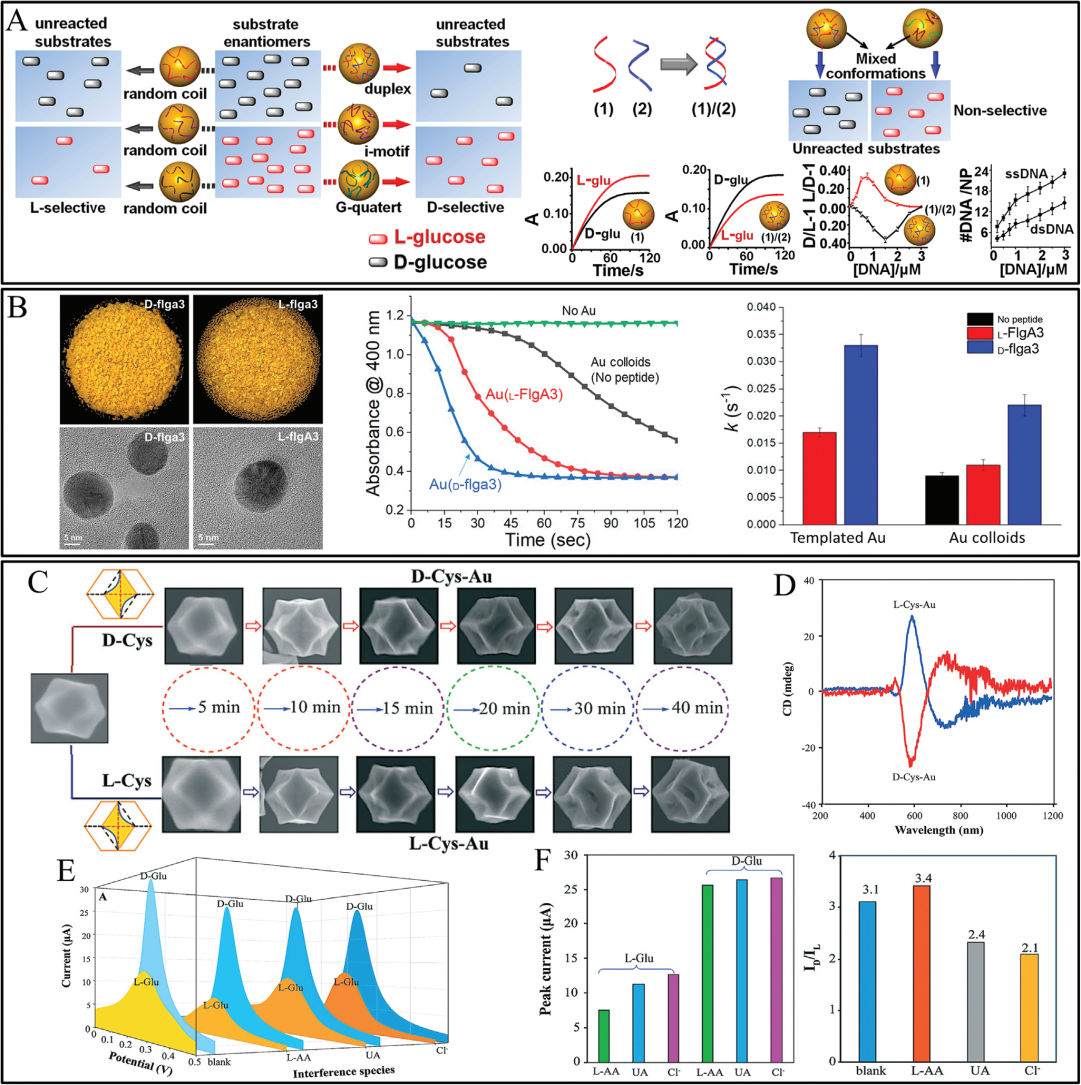
A) Schematic illustration of DNA functionalized Au NPs for the enantioselective oxidation of glucose enantiomers.^[^ ^60^ ^]^ B) Structure of Au NPs synthesized with D‐flga3 and L‐FlgA3, and corresponding Catalytic activity of D‐ and L‐peptide templated Au NPs for reduction of 4‐nitrophenol to 4‐aminophenol with catalysis selectivity.^[^ ^59^ ^]^ C) SEM images of the as‐synthesized D‐Cys‐Au and L‐Cys‐Au. D) The CD spectra. (E,F) The differential pulse voltammogram curves E) and selective catalytic oxidation of D‐Glu and L‐Glu in the presence of L‐ascorbic acid, urea, and chloride ions (F).^[^ ^6^ ^]^

Besides the most reported amino acids, other biomolecules (such as DNA, peptide, and saccharide) were also alternative selectors for the scale‐up nanozymes with high selectivity. DNA served as an environment‐responsive chiral ligand and was functionalized on Au NPs for the selective catalytic glucose enantiomers. The structures of DNA molecules were designed as random‐coiled DNA and structured DNA (duplex, i‐motif, and G‐quadruplex). It was found that L‐glucose was readily catalytic oxidized by random‐coiled DNA‐modified Au NPs, and D‐glucose was easily catalytic oxidized by structured DNA‐modified Au NPs (**Figure** **4**A).^[^ ^60^ ^]^ The folded conformation of DNA was controlled at pH 5.2, and exhibited better catalytic selectivity toward D‐glucose, whereas, unfolded structures of DNA were controlled at pH 7.2, which displayed better catalytic selectivity toward L‐glucose (Figure 4A). The multistranded structures of DNA endowed Au NPs with binding sites for the combination with D‐glucose rather than L‐glucose.^[^ ^60^ ^]^ The work provided a bridge to engineer new selective enantioselective catalysis depending on the interaction between nucleic acids and saccharide, and DNA as chiral selectors could also be modified on other high catalytic active metallic nanostructures.

The use of peptides has been rare for generating and controlling optical chirality in nanomaterials, in which the fine structure is not well understood due to the complexity of peptide−nanoparticle interactions. Based on D‐cys‐D‐his, D‐cy‐L‐his, and L‐cys‐D‐his as the chiral modification agents, Cu NPs showed peroxidase‐mimicking activity in 3,3′,5,5′‐tetramethylbenzidine oxidation and chiral selectivity toward 3,4 dihydroxy‐D,L‐phenylalanine (D, L‐DOPA).^[^ ^58^ ^]^ Importantly, D‐cys‐D‐his functionalized Cu NPs presented higher peroxidase‐mimicking catalytic activity against D‐DOPA than L‐DOPA. which demonstrated the stereoselective recognition Cu NPs play the catalytic center role and chiral dipeptide ligands play the inducer role. It was reported that the complex interactions between D‐peptides and gold nanomaterials led to a chiral restructuring of peptides and proteolytic cleavage of D‐peptides (Figure 4B).^[^ ^59^ ^]^ The Au NPs stabilized by D‐peptide produce a highly ordered atomic surface and restructured peptide bonds for enzyme cleavage. Differences in gold nanoparticle‐catalyzed reduction of 4‐nitrophenol were observed on the basis of the chiral peptide used in nanoparticle synthesis. The catalytic activities of Au NPs stabilized by D‐flgA3 and L‐FlgA3 showed increasing catalytic activity and selectivity for the reduction of 4‐nitrophenol to 4‐aminophenol. The D‐flga3 and L‐FlgA3 templated Au NPs yielded rate constants of 0.033 s^−1^ and 0.017 s^−1^, respectively (Figure 4B). The higher selectivity for D‐flga3 Au NPs was conducted that the higher ordered arrangement of surface gold atoms and/or its higher accessibility to substrate. The proteolytic cleavage of D‐peptides modified NPs provides an opportunity for designing therapeutics to treat peptide venoms.

The catalytic chiral metal nanocrystals showed low enantioselectivity in chiral synthesis and limited their wide applications. It was reported that chiral gold nanocrystals form trioctahedral gold nanocrystals displayed asymmetric structural evolution induced by D/L‐Cys.^[^ ^6^ ^]^ The Au NPs offer unique chiral morphology with exposed high‐index facets (Figure 4C). The formed D‐Cys‐Au and L‐Cys‐Au under optimized conditions exhibited high enantioselectivity and catalytic activity for the catalytic oxidation of glucose enantiomers (Figure 4C–F). The catalytic activity of L‐Cys‐Au for D‐glucose was 3.4‐fold higher than that for L‐glucose. The catalytic performance of Au NPs was highly dependent on the chiral morphology and chiral ligand in which the increase in the coverage of L‐cystine on L‐Cys‐Au resulted in reduced catalytic activity but improved enantioselectivity. It was shown that the L‐ascorbic acid, urea, and chloride ions only resulted in a slight reduction in the differential pulse voltammogram signal toward catalysis of D‐Glu. It was concluded that the D‐Glu molecules adsorbed on the surface of L‐Cys‐Au due to the interaction between L‐Cys‐Au and D‐Glu. The high catalytic activity toward the oxidation of D‐Glu provided routes to the design of chiral nanozymes with geometries.

Additionally, the non‐supported chiral Ir NPs were synthesized for enantioselective hydrogenation of prochiral ketones with ee as 55% and yield of 88%.^[^ ^45^ ^]^ The NPs could also be immobilized on support such as GO for asymmetric catalysis. For example, GO‐supported nickel catalyst was synthesized for asymmetric hydrogenation. The prepared hybrid catalyst exhibited a high turnover frequency (20 160 h^−1^) and enantiomeric excess of 98.6% for the asymmetric hydrogenation of methyl acetoacetate.^[^ ^219^ ^]^ The large specific surface area of GO helped to maintain and stabilize the Ni particles which allowed for high carrier mobility and enhanced reactant adsorption.

**Figure 5:**
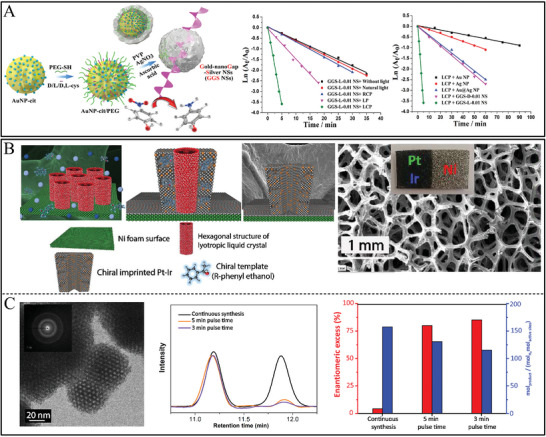
A) Schematic illustration for the synthesis of optically active gold core‐DNA‐silver shell NPs and their selective photocatalysis.^[^ ^76^ ^]^ B) The synthesis of a chiral encoded mesoporous bimetallic Pt‐Ir alloy supported on Ni foam. C) The corresponding asymmetric catalysis with chiral encoded Pt‐Ir alloy.^[^ ^220^ ^].^

Complex or heterogeneous metal NPs usually display multi‐functionality and exhibit high catalysis activity. By using L‐DOPA and D‐DOPA as chiral templates, the chiral bi‐metallic Pt‐Ir NPs electrodes were fabricated through co‐electrodeposition of H_2_PtCl_6_ and H_2_IrCl_6_.^[^ ^84^ ^]^ In comparison to monometallic nanostructures, bi‐metallic Pt‐Ir NPs engineered electrodes showed well‐enantioselective asymmetric catalysis of acetophenone (>95% ee). Bi‐metallic Pt‐Ir NPs engineered electrodes were stable and still reached up to 90% ee within 3 cycles. The good enantioselective recognition and stability ensured highly efficient stereoselective catalysis. Chiral nanomaterials could absorb the circularly polarized light and generate much more hot electrons for the catalytic acceleration of chemical reactions. For example, chiral Au‐gap‐Ag nanostructures were designed by modifying Cys enantiomers in the interior nanobridged gaps, which showed adjustable plasmon chiral signals from 350 nm to 600 nm (**Figure** **5**A).^[^ ^76^ ^]^ The maximum g‐factor of chiral Au‐gap‐Ag nanostructures reached up to 1 x 10^−2^ at 430 nm. Chiral Au‐gap‐Ag nanostructures showed superior catalytic behaviors for the reduction of 4‐nitrophenol under the excitation of circularly polarized light. Chiral Au‐gap‐Ag nanostructures demonstrated elevated catalytic efficiency, which was 73 times and 17 times stronger than Au NPs and Au@Ag core‐shell NPs, respectively (Figure 5A). The chiral complex nanostructures demonstrated enhanced catalytic efficiency under the excitation of right‐circularly polarized light 12 times higher than those of the no light. It was expected to explore chiral nanomaterials with unusual circularly polarized photocatalytic activity for chiral catalysis. The complex metal nanostructures displayed great research value in realizing high catalytic activity, and high selectivity in the aqueous phase.

For the applications of enantioselective catalysis, it was necessary to separate the products from the mixtures and achieve the reuse of chiral nanomaterials in liquid phase. It was essential to fabricate solid‐like chiral nanomaterials for enantioselective catalysis. Recently, based on the lyotropic liquid crystalline phase in the presence of metal salt and chiral molecules on Ni foam, the electrodeposition was performed through the assembled template, and the chiral imprinted mesoporous Pt‐Ir alloy supported on Ni foam was constructed. The fabricated nanostructured chiral Pt‐Ir alloys on nickel foams showed high catalysis activity for the asymmetric hydrogenation of aromatic ketones (Figure 5B,C).^[^ ^220^ ^]^ Optimized experimental conditions achieved high enantioselectivity (>80%) and good stability. The development of heterogeneous solid‐state catalysts for asymmetric synthesis is still challenging for achieving enantiomerically pure products.

### 3.2 Chiral Metal Oxide NPs‐Based Catalysis

The construction of economical and sustainable catalysts was an emerging and important topic for both academic and industrial concerns.^[^ ^91^ ^]^ Although various metal oxide chiral NPs with useful applications have been explored, the main application in the field of catalysis is most appealing, originating from its unique structure and atomic properties.^[^ ^93^ ^]^ Chiral metal oxide NPs have been reported for asymmetric hydrogenation, oxidation, and other related catalytic reactions.^[^ ^91^, ^109^, ^221^ ^]^ Metal oxide chiral NPs generally displayed superior catalytic activity with great potential for industrial applications.^[^ ^221^ ^]^

**Figure 6:**
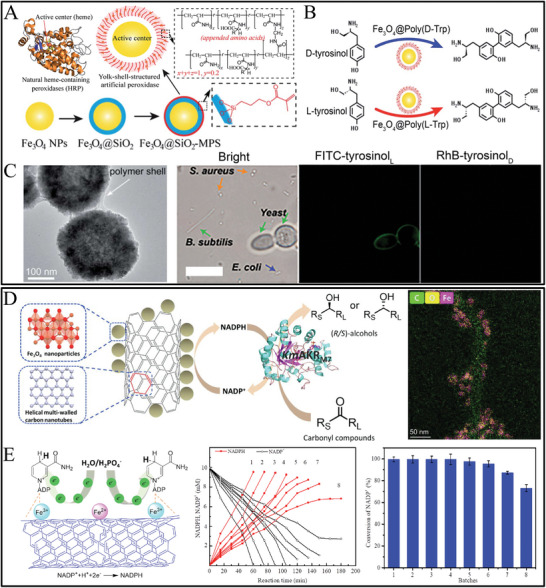
A) Representative structures of natural heme‐containing peroxidase (left) and yolk–shell‐structured artificial peroxidase (right), and the preparation procedures of the yolk–shell‐structured artificial peroxidase. B) The enantioselective oxidation of chiral tyrosinol catalyzed by Fe_3_O_4_@Poly(L‐/D‐Trp). C) TEM images of yolk–shell Fe_3_O_4_@Poly(L‐Trp), and Fe_3_O_4_@Poly(L‐Trp) NPs catalyze FITC‐L‐tyrosinol to label yeast cell (Scale bars: 10 mm).^[^ ^102^ ^]^ (D,E) Asymmetric biocatalysis of chiral alcohols by Fe_3_O_4_@helical multi‐walled carbon nanotubes and corresponding element mapping results (D). schematics of NADP+ hydrogenation by Fe^2+^ and Fe^3+^ on Fe_3_O_4_@ HCNTs. Batch reactions of NADP^+^ hydrogenation catalyzed by Fe_3_O_4_@HCNTs.^[^ ^223^ ^]^

Firstly, the magnetic NPs provided efficient catalytic activity and enabled facile recovery of the catalyst from the reaction medium which was extremely important in modern synthetic chemistry.^[^ ^100^, ^101^ ^]^ The sulfonated magnetic mesoporous nanocomposite, Fe_3_O_4_@MCM‐41@NH‐SO_3_H, showed effective catalytic activity in the formylation of amines and alcohols in the reaction with formic acid, with a yield of 92% and a reaction time of 15 min.^[^ ^222^ ^]^ when the reaction was catalyzed using Fe_3_O_4_ NPs, it was only slightly facilitated, whereas the conversion was significantly more effective when using this nanocomposite. The reason for this difference can be summarized in two points: the high surface area of the mesoporous structure with a large number of hydroxyl groups, and the limited acidity brought to the composite nanomaterial by the sulfonic acid functional group. Chiral Fe_3_O_4_@Poly(amino acids) nanozymes were designed by using Fe_3_O_4_ NPs as the catalytic yolk‐core owing to the intrinsic peroxidase‐like activity and chiral selectivity through amino acids. The different amino acids such as D‐/L‐Phe, D‐/L‐Trp, D‐/L‐His, and D‐/L‐aspartic acid (Asp) exploited different affinity against substrate. It was found that achiral Fe_3_O_4_ NPs did not have stereoselectivity against tyrosinol enantiomers, the His and Phe‐modified Fe_3_O_4_ NPs showed poor enantioselectivity, and the Asp‐modified Fe_3_O_4_ NPs showed moderated enantioselectivity, while the Trp modified Fe_3_O_4_ NPs showed best stereoselectivity (**Figure** **6**A–C).^[^ ^102^ ^]^ It was found that Fe_3_O_4_@Poly(Trp) NPs showed better affinity toward D‐tyrosinol, in which the κ_cat_/K_M_ value was 5.38 times higher than L‐tyrosinol. This endowed Fe_3_O_4_@Poly(Trp) NPs with high enantioselectivity for the catalytic oxidation of tyrosinol. Different from the reported nanozymes, the designed yolk‐shell nanostructures endowed nanozymes with more sites for the modification of chiral selectors, which further improved the enantioselectivity. Alternatively, cofactor nicotinamide adenine dinucleotide phosphate (NADPH) is important, while its regeneration from glucose dehydrogenase generally suffers from massive glucose consumption and low atom efficiency. The Fe_3_O_4_ NPs on helical multi‐walled carbon nanotubes (Fe_3_O_4_@HMWCNTs) showed comparable transfer hydrogenation activity to Exiguobacterium sibiricum glucose dehydrogenase (Figure 6D,E).^[^ ^223^ ^]^ The established chemoenzymatic catalysis combined the aldo–keto reductase KmAKR with Fe_3_O_4_@HMWCNTs scaffold, and asymmetrically synthesized optically pure tert‐butyl 6‐cyano‐(3R,5R)‐dihydroxyhexanoate ((3R,5R)−2, > 99.5%). Compared to the previously established biocatalytic approach for NADPH regeneration, the developed strategy required neither glucose/alcohols as co‐substrate nor a glucose dehydrogenase/alcohol dehydrogenase.

It was demonstrated that enantioselectivity of chiral products can be obtained from achiral reagents by magnetically influencing the preferred direction of electron spin. For example, the hematite (Fe_2_O_3_) was used to catalyze the oxidation of sulfide to sulfoxide and the Diels – Alder cycloaddition reaction.^[^ ^224^ ^]^ Exploiting novel chiral nanozymes for specific catalytic more biomolecules has become the new direction of catalysis. Chiral nanomaterials not only specifically recognized and cleaved proteins but also could induce the catalytic formation of peptide bonds. The presence of Pro and Asp on chiral WO_3‐x_·H_2_O NPs induced the catalytic synthesis of peptide bonds, such as Asp‐Pro and Asp‐Asp dipeptides.^[^ ^108^ ^]^ Chiral nanomaterials possessed enzyme‐mimetic activities and opened the way for the catalytic formation of peptide bonds.

### 3.3 Chiral Semiconductor NPs Based Catalysis

In comparison to the commonly reported NPs, zero‐dimensional QDs had small sizes of less than 10 nm, possessed prominent edge and quantum confinement effects, and would improve the enantioselectivity.^[^ ^29^ ^]^ Chiral nanomaterials possessed unique enzyme‐mimetic activities, but there was a great challenge to enhance the mimicking enzymatic enantioselectivity^[^ ^225^ ^]^. Chirality was an intriguing characteristic of life and had a close relationship with the most important biological processes. Biological macromolecules (such as proteins, peptides, DNA, etc) also showed intrinsic chiral characteristics.^[^ ^28^ ^]^ Chiral semiconductor NPs showed unique recognition toward small molecules and macromolecules and generated highly efficient catalysis activity.

**Figure 7:**
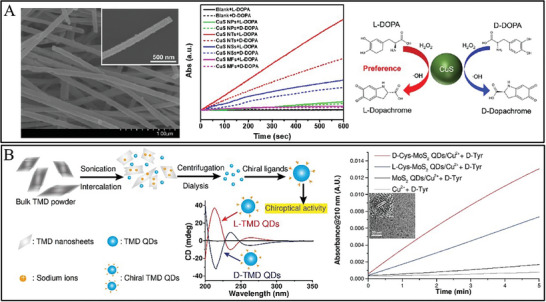
A) Structures of CuS nanotubes and the time‐dependent catalysis of chiral DOPA at 475 nm at 600 s intervals, and the mechanism of enantioselective oxidation of chiral DOPA by CuS.^[^ ^37^ ^]^ B) Schematic illustration for the sonication combined with ion intercalation for preparation of transition metal dichalcogenides chiral QDs, and the corresponding typical CD spectra.^[^ ^48^ ^]^

Based on chiral ligands and metal precursors involved synthesis of chiral nanomaterials strategy, chiral hexagonal CuS structures including CuS NPs, CuS nanotubes, CuS nanosheets, and microflowers with negative CD bands were fabricated (**Figure** **7**A).^[^ ^37^ ^]^ The CuS structures showed structure‐dependent chiroptical activity and demonstrated the negative CD was derived from a common microstructure in CuS, in which the (Cu_3_S_3_)_2_ nanostructure existed in hexagonal CuS. The chiral CuS showed enantioselective catalysis activity for D‐DOPA and L‐DOPA oxidation that CuS nanotubes had the highest catalysis activity due to more active sites and large anisotropy. The CuS displayed peroxidase‐like activity that induced hydroxyl radicals (•OH) in the presence of H_2_O_2_, and catalyzed the DOPA oxidation into dopachrome. Similarly, the amino acids modified on the surface of QDs displayed a catalysis effect for the catalysis oxidation tyrosinol enantiomers. Chiral MoS_2_ and WS_2_ QDs were prepared by using Cys and Phe as ligands, respectively. chiral MoS_2_ and WS_2_ QDs showed well peroxidase‐like activity and possessed excellent enantioselectivity of 6.77 for D‐ and L‐tyrosinol in copper ions solutions (Figure 7B).^[^ ^48^ ^]^ Thanks to high binding for chiral QDs toward tyrosinol enantiomers, the catalysis had high enantioselectivity.

**Figure 8:**
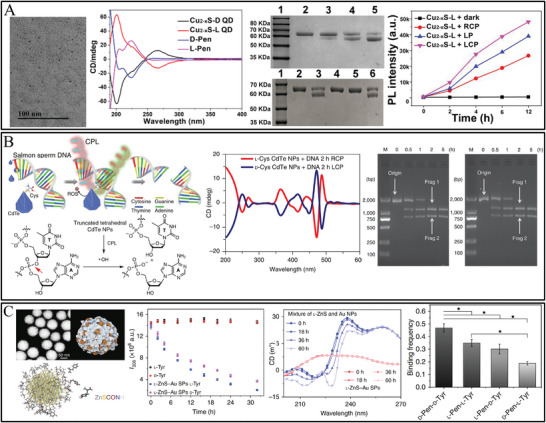
A) The TEM and CD spectra of D‐/L‐penicillamine synthesized chiral copper sulfide QDs and the CPL triggered selective catalysis cleavage of BSA.^[^ ^110^ ^]^ B) Schematic illustration of CPL triggered chiral CdTe‐based specific DNA cleavage and the electrophoresis results of L‐Cys CdTe and D‐Cys CdTe nanoparticles with 1,839 bp DNA illuminated with 405 nm RCP/LCP for 2 h.^[^ ^116^ ^]^ C) Morphology and spectra characterization of chiral ZnS–Au supraparticles, the selective photocatalytic activity with L‐ZnS–Au SPs for different Tyr enantiomers and the corresponding binding frequency decreases in the order from D‐Pen‐D‐Tyr, L‐Pen‐L‐Tyr, L‐Pen‐D‐Tyr, to D‐Pen‐L‐Tyr.^[^ ^25^ ^]^

New insights have been provided for the cutting of protein and DNA by using chiral nanomaterials. The D/L enantiomers had a distinct binding affinity in the interaction with biological macromolecules, and chiral nanomaterials featured with chiral ligands exhibited improving biocompatibility and different biocatalytic behaviors. Chirality was found to influence the protein transport and could recognize protein for specific cutting.^[^ ^110^, ^226^ ^]^ Bovine serum albumin (BSA) was chosen as a protein model to study the biocatalysis performances of chiral nanomaterials. Cys enantiomers were functionalized on the nanopores, in particular, L‐cys‐modified nanoprobes exhibited good selectivity for BSA due to their chiral interaction, dominating the process of protein translocation. This study indicated that chirality was highly associated with the protein translocation process (**Figure** **8**A).^[^ ^110^ ^]^ Chiral Cu_2‐x_S QDs were prepared by the modifying of chiral D‐/L‐Pen. It was found that chiral Cu_2‐x_S QDs showed chiral peaks at 608 and 863 nm, in which the g‐factor of chiral Cu_2‐x_S QDs reached up to 1.0 x 10^−2^. It was shown that L‐Pen modified Cu_2‐x_S QDs exhibited the highest photocatalytic efficiency under left circularly polarized light irradiation, and ≈79% BSA was cleaved by L‐QDs. The isothermal titration calorimetry test displayed that L‐Pen modified Cu_2‐x_S QDs had well association with BSA, and the affinity constant was 3.0 x 10^−5^ M, whereas the binding between D‐Pen modified Cu_2‐x_S QDs and BSA was 2 times weaker. Under left circularly polarized light irradiation, L‐Pen modified Cu_2‐x_S QDs generated •OH to cleave BSA.^[^ ^110^ ^]^ This method provides potential routes for photocatalytic protein cleavage by using chiral semiconductors under the excitation of light. There is a demand to develop chiral nanomaterials with known abiotic function, superior catalytic performances, and good biocompatibility.

For the catalysis of macromolecules, the chiral nanomaterials should possess both catalysis selectivity and high efficiency. Cys enantiomers modified CdTe QDs were reported to possess high selectivity and well‐restriction endonuclease mimicking performances for gene editing. Gene editing was mainly determined by biologically engineered nucleases for gene manipulation at the molecular level. Interestingly, chiral CdTe QDs were found to photoinduce cleave DNA. Chiral CdTe QDs were designed by using Cys enantiomers as ligands and showed the function of mimicking a restriction endonuclease (Figure 8B).^[^ ^116^ ^]^ Chiral CdTe QDs specifically recognized the site of GAT′ATC in dsDNA with a length exceed of 90 bp, attributing to the binding of Cys and the conformed DNA sequences. Under irradiation of circularly polarized light, ROS was generated for the cleavage of phosphodiester (Figure 8B). L‐cys functionalized CdTe QDs possessed better cleavage rate upon right circularly polarized light, and D‐cys modified CdTe QDs showed better cleavage rate under left circularly polarized light.^[^ ^116^ ^]^ The non‐enzymatic sequence‐specific DNA incision of chiral CdTe QDs enabled the exploration of abiotic materials for gene editing and other biological applications. The cytotoxicity effect of chiral CdTe QDs is still a concern for long‐time practical applications.

In comparison to single component dependent NPs, supraparticles by compositing multicomponent of inorganic metal NPs, semiconductors, or ceramic, may exhibit enhanced catalytic activity for the efficient enantioselective catalysis.^[^ ^227^ ^]^ The characteristic supraparticles architectures generally offered collective properties from individual building blocks enabling huge benefits of catalyzing reactions. For example, chiral supraparticles were fabricated by using 3 ± 0.7 nm ZnS NPs and 2 ± 0.3 nm Au NPs by utilizing Pen as chiral ligands, and the average sizes reached up to 70–100 nm (Figure 8C).^[^ ^25^ ^]^ Chiral ZnS supraparticles exhibited excellent photocatalytic ability, which could catalyze Tyr into dityrosine. A decreased fluorescence signal was observed at 306 nm due to the consumption of Tyr, and an enhanced fluorescence signal was observed at 414 nm due to the formation of dityrosine. The best catalytic activity was D‐Pen‐modified ZnS supraparticles for the catalysis of D‐Tyr into dityrosine. Chiral ZnS‐Au supraparticles could further converse Tyr to dityrosine and even DOPA. The chiral ZnS‐Au supraparticles showed enhanced photocatalytic conversion for enantioselective oxidization.^[^ ^25^ ^]^ In another example, Cys enantiomers were applied as chiral ligands for the fabrication of chiral ZnS supraparticles. Chiral ZnS supraparticles featured with CD peaks from 265 nm to 400 nm could absorb the light of 300–450 nm photons and demonstrated well photocatalytic activity for the transformation of Tyr to DOPA and Tyr dimers.^[^ ^49^ ^]^ Chiral ZnS supraparticles possessed a high transformation of 23%−26%, which was 10 times higher than that of metalorganic compounds. More supraparticles need to be further exploited in the presence of different components and chiral ligands, and their mimetic enzyme functions are still unclear, as well as the characteristics of each component are still unknown.

### 3.4 Chiral Carbon Materials Based Catalysis

Besides typical inorganic NPs, metal‐free chiral nanomaterials were also widely reported for catalysis applications, such as chiral CDs, and carbon nanotubes, that act as the antioxidant multienzyme.^[^ ^228^ ^]^ The excellent capacity for enantiomeric recognition provided a large potential for enhanced chiral catalysis.^[^ ^132^ ^]^

CDs usually exhibit peroxidase‐like, catalase‐like, and superoxide dismutase‐like activities, and have the capability to efficiently deplete the excessive ROS such as peroxide (H_2_O_2_), superoxide anion (O_2_^−^) and •OH by surface abundant functional groups.^[^ ^133^ ^]^ It was found that the chiral effects of CDs enhance the glucose oxidase activity to achieve better highly electrochemical performance.^[^ ^140^ ^]^ The glucose oxidase attached to CDs catalyzes the formation of glucose into gluconic acid and a proton, in which the chiral CDs direct electrochemistry of glucose oxidase, and the complex exhibited superior electrocatalytic activity. The citric acid and D‐Pro fabricated CDs exhibited high asymmetric catalytic activity for the direct aldol condensation of P‐nitrobenzaldehyde and cyclohexanone with a yield of up to 98% and an ee% of 471%.^[^ ^46^ ^]^ The CDs fabricated from the electrooxidation polymerization method showed enantioselective catalytic activity for DOPA oxidation with high stability.^[^ ^141^ ^]^ During catalysis, L‐CDs showed a lower Km value (92.92 µM) for L‐DOPA than D‐DOPA (183.64 µM), suggesting that the binding affinity between L‐C‐Dots and L‐DOPA was stronger than that of L‐C‐Dots with D‐DOPA. Based on L‐CDs catalyst, the parameter kcat/Km for L‐DOPA and D‐DOPA are 2.12 × 10^−2^ and 1.09 × 10^−2^ s^−1^ M^−1^, indicating a 1.95‐fold selectivity factor for L‐DOPA against D‐DOPA. The D‐CDs showed a selectivity factor of 2.11 for D‐DOPA against L‐DOPA. The chiral CDs made from glucose exhibited selective biocatalysis, in which L‐CDs are more likely to catalyze L‐Trp than D‐ Trp with a selective factor of 1.60, whereas the D‐CDs indicated a selective factor of 0.63 for D‐ Trp against L‐ Trp.^[^ ^39^ ^]^

**Figure 9:**
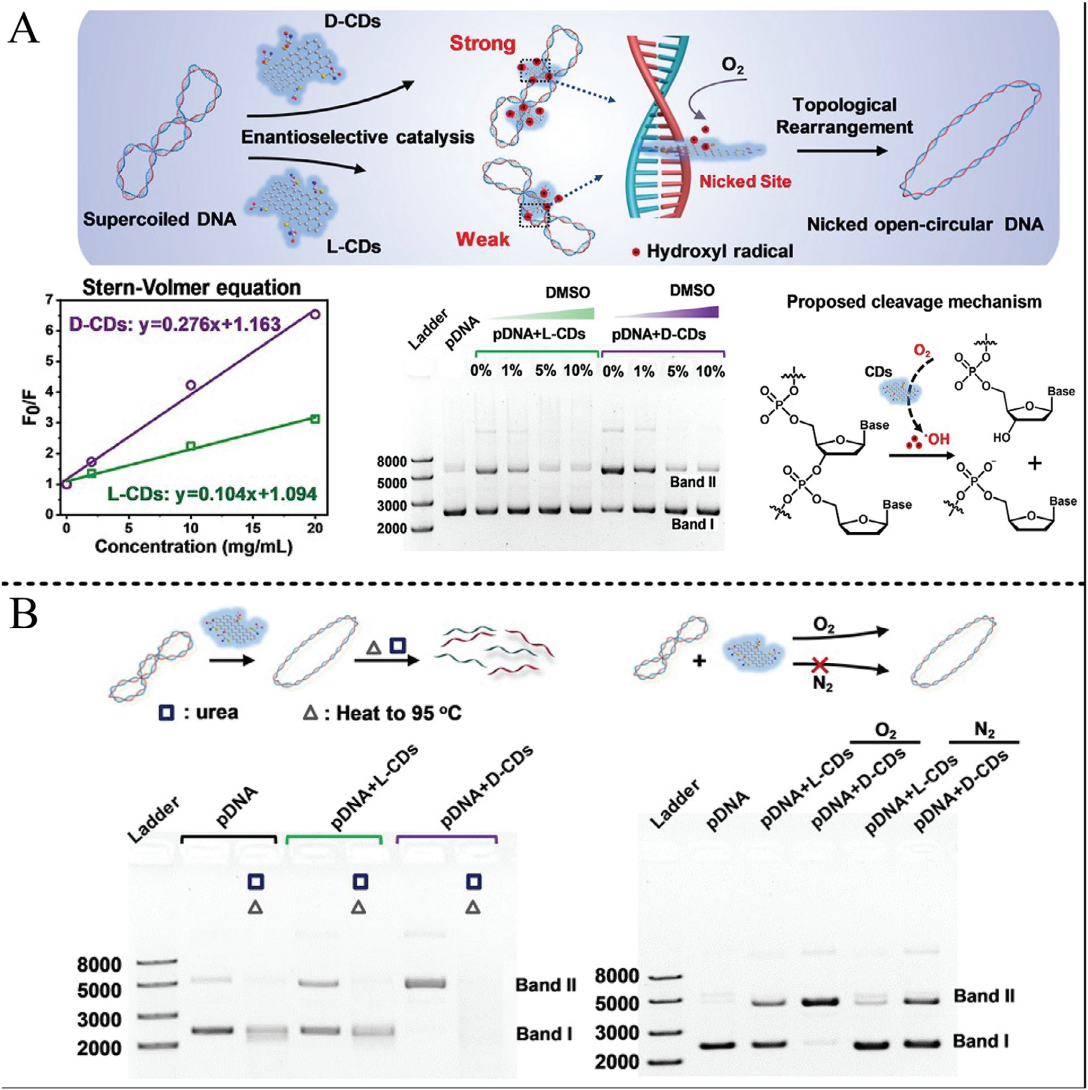
A) The schematic illustration for chiral carbon Dots catalysis of plasmid DNA. B) The corresponding agarose gel electrophoresis for analysis of the selective catalysis of plasmid DNA.^[^ ^229^ ^]^

Chiral ligands enabled CDs showed good enantioselectivity for the application of biochemical catalytic reactions. Chiral CDs were designed by using Cys as chiral ligands and were exploited to exhibit topoisomerase I mimicking behaviors. L‐cys modified chiral CDs and D‐cys modified chiral CDs showed mirror chiral signals. It was found that DNA double helix was easily combined with D‐cys than L‐cys. Therefore, more •OH would be generated for D‐cys modified chiral CDs to destroy the phosphate backbone of DNA (**Figure** **9**A).^[^ ^229^ ^]^ In comparison to L‐cys‐modified chiral CDs, D‐cys‐modified chiral CDs could effectively catalyze the topological transition of plasmid DNA from supercoiled to nicked open‐circular configuration (Figure 9A,B). In comparison to the association between L‐cys and DNA, the higher association between D‐cys and DNA was proved by molecular dynamics simulation, which was due to the formation of hydrogen bonds and hydrophobic interaction, which was the origin of enantioselectivity. The chiral CDs‐dependent topoisomerase I mimicking activity showed potential prospects for the applications of gene editing and protein engineering. Chiral CDs with enantioselectivity were prepared and possessed excellent biocompatibility for biocatalytic applications.

In addition to chiral CDs, chiral Lewis acids integrated with single‐walled carbon nanotubes showed asymmetric catalysis in the enantioselective conjugate addition of benzaldoxime in water.^[^ ^146^ ^]^ The chiral carbon nanotube demonstrated enhanced reactivity, stereoselectivity, and long‐term stability in high yields. The developed method enabled high reactive and stereoselective catalytic systems that not only improved existing synthetic methods but also invented distinct chemical reactions. The designed catalysts in water provided an expedient, highly efficient pathway to obtain optically active chemicals. Furthermore, chiral multi‐walled carbon nanotubes (MWCNTs) were also prepared in the presence of D‐Phe.^[^ ^38^ ^]^ Chiral D‐Phe modified MWCNTs showed well electrocatalytic ability toward 2,2,2‐trifluoroacetophenone, in which 2,2,2‐trifluoroacetophenone was electrocatalytically reduced to α‐(trifluoromethyl) benzyl alcohol with the yield of 65% and the enantiomeric excess of 40%. The nonmetallic chiral nanomaterials demonstrated acceptable asymmetric electroreduction activity, considerable stability, and favorable reusability.

Additionally, graphite oxide (GrO), GO were prepared from graphite and graphene as starting materials.^[^ ^156^, ^230^ ^]^ Pro impregnation allowed the synthesis of Pro‐GrO and Pro‐GO chiral catalysts.^[^ ^230^ ^]^ The developed Pro‐GrO and Pro‐GO catalyzed asymmetric aldol reaction between 2‐nitrobenzaldehyde and acetone with a high selectivity of 90% and enantioselectivity of 77%. The graphene‐fabricated chiral GO usually provides more activity than those prepared from graphite, while the catalyst's lifetime is still limited. Multiple chiral salen Ti^IV^ decorated on GO edge and planes displayed highly efficient asymmetric sulfoxidation with high yields (96%) and excellent enantioselectivities (98%) that were more active than traditional chiral salen Ti^IV^.^[^ ^231^ ^]^ Additionally, it was reported that the GO was functionalized with zinc finger‐protein‐like α‐helical chiral metallo‐supramolecular complex ([Fe_2_L_3_]^4+^) and displayed excellent peroxidase mimic catalysis activity.^[^ ^232^ ^]^ Such GO chiral nanostructure generated remote enzyme cascade biocatalysis reactions by transparent near‐infrared light.

### 3.5 Chiral Framework Materials‐Based Catalysis

**Figure 10:**
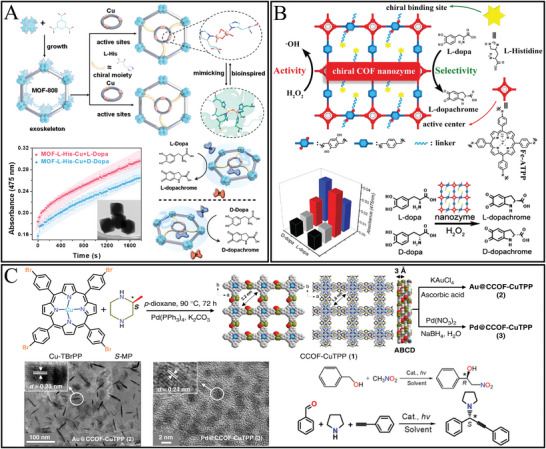
A) Schematic illustration of the preparation process of MOF‐L(D)‐His Cu. The fabricated MOF‐L‐His‐Cu, chiral MOF‐L(D)‐His‐Cu as catechol oxidase mimic for chiral Dopa catalysis.^[^ ^236^ ^]^ B) Schematics of construction of a chiral COF nanozyme, which selectively catalyzed H_2_O_2_‐mediated oxidation of dopa enantiomers to corresponding dopachrome.^[^ ^170^ ^]^ C) Synthesis of Single 2D layer and crystal patterns of porphyrin‐containing COF‐Cu, Au@COF‐Cu, and Pd@COF‐Cu frameworks, and the corresponding TEM images (side and top views). The catalyzed model one‐pot asymmetric Henry reaction and catalyzed asymmetric coupling reactions.^[^ ^245^ ^]^

Fabrication of highly active and selective framework materials for enzyme‐mimicking catalysis remains a challenge for a long time.^[^ ^233^ ^]^ Framework materials featured with metal ions organic linkers showed porous structures and good catalytic activity for asymetric catalytic applications.^[^ ^41^ ^]^ For example, Chiral MOF nanosheets assembled by helical metal–organic chains within microemulsion exhibit higher absorption efficiency toward the Cr(ox)_3_^3−^ complex with the ee value up to 82%.^[^ ^234^ ^]^ Chiral NU‐1000 was prepared through the introduction of chiral carboxylic groups, which were used as asymmetric support in order to fabricate chiral catalysts [C‐NU‐1000‐Mo] with molybdenum catalytic active centers as Lewis acid sites.^[^ ^235^ ^]^ Chiral catalysts [C‐NU‐1000‐Mo] showed enantioselective catalysis activity of alkens to epoxides epoxidation. The chiral catalysts [C‐NU‐1000‐Mo] based catalysis showed good enantiomeric excess, elevated selectivity to an epoxide (up to 100%), and good stability. Additionally, a better understanding of the catalytic mechanism will help to develop efficient and selective enzyme‐mimicking catalytic nanomaterial. It was reported that the chiral His coordinated copper core grafted onto Zr‐based MOF materials showed the bimetal active site of natural catechol oxidase (**Figure** **10**A).^[^ ^236^ ^]^ The biomimetic construction endowed MOF‐His‐Cu with catechol oxidase‐like activity that catalyzes dehydrogenation and oxidation of o‐diphenols. It was found that the precise incorporation of chiral His induced higher catalytic selectivity to chiral catechol substrates even than natural enzymes. The binding energy and potential steric effect in active site interactions resulted in high stereoselectivity. Despite of synthetic modification of MOF, the heterogenous linear chiral proline impregnated in the MOF cavities enabled good enantioselectivities (ee, 96%) and yield (89%) for catalyzing the asymmetric Aldol reaction between p‐nitrobenzaldehyde and cyclohexanone.^[^ ^47^ ^]^ Additionally, the *N*‐(tert‐butyloxycarbonyl)pyrrolidine‐S(R)−2‐(3,5‐bis(4′(pyridin‐4‐yl)‐[1,1′‐biphenyl]−3‐yl)−4H‐1,2,4‐triazol‐4‐yl)carbamoyl was adopted as the ligand to design chiral MOF.^[^ ^237^ ^]^ The chiral MOF can catalyze asymmetric cross‐coupling between carbonyl and aryl radicals with a remarkable conversion ratio (99%) and ee value (92%−93%). By switching on photoredox activation and asymmetric aldol reaction, this MOF works well in catalyzing secondary amine‐mediated asymmetric b‐arylation arylation with conversion ratio (97%−98%) and ee value (85%−87%) and a‐carbonyl with conversion ratio (96%−98%) and ee value (82%−85%) of saturated aldehydes and ketones, respectively. The method provided a good candidate to construct heterogeneous and homogeneous catalysts.

An organic‐inorganic hybrid microreactor can be prepared using permeable microporous silica hollow nanospheres. The micropores provide short mass transfer channels for small molecules alone and are hydrophobic and hydrophilic to facilitate aqueous‐mediated catalysis.^[^ ^238^ ^]^ It has been applied to catalyze aqueous asymmetric transfer hydrogenation ketones. For example, for the small substrate acetophenone, the conversion was 98% and ee 95%, which is comparable to some homogeneous catalysts. The chiral frustrated Lewis pair with MOF showed good catalytic performance for heterogeneous asymmetric hydrogenation of imines with 97% yield and 86% ee value.^[^ ^239^ ^]^ By tailoring the vicinity of chiral Lewis pair with some auxiliary sites, the resultant MOF achieved catalysis enantioselectivity and chemoselectivity, respectively, demonstrating good catalysis activity in heterogeneous hydrogenation of α,β‐unsaturated imines to afford chiral β‐unsaturated amines with high yields (93%, 95%) and ee values (81%, 86%).^[^ ^240^ ^]^ Alternatively, based on chiral MOF derived from valine (Val)‐, Ser‐, and threonine (Thr), the Pt NPs supported by Thr‐MOF showed good enantioselectivity and catalytic recoverability for asymmetric hydrogenation reaction that may be due to synergistic effect.^[^ ^18^, ^169^ ^]^ The MOF complexed Pt NPs not only exhibited the highest experimental conversion (50.6% and 53.5%) but also had high enantioselectivity for ethyl lactate, with ee values of 18.1% and 20.1%, respectively. The tunable chiral MOF with tailored photocatalytic performance synthesized through coordinating an organic ligand with different metal ions exhibits significantly improved conversion and stereoselectivity in asymmetric a‐alkylation of aldehydes under visible light^[^ ^241^ ^]^ Additionally, in the dissociation equilibrium using enzyme‐mediated MOF (represented by UiO‐66), the enzyme can compete with the proto‐ligand for coordination, while the release of the MOF metal clusters creates defects, thus facilitating the gradual transport of the enzyme from the surface to the interior.^[^ ^242^ ^]^ The catalysts prepared in this way showed excellent catalytic activity when applied to the chiral splitting of (R,S)‐phenylethanol, with ee values > 99%, a conversion rate close to 50%, and an activity of up to 80% after 5 cycles.

In comparison to inorganic NPs, chiral COF nanozyme may possess superior enzyme‐like behaviors owing to the presence of well‐dispersed iron porphyrin and superior structures. L‐His was applied as chiral selectors for the selective catalytic oxidation of L‐DOPA, and the selectivity factor was up to 1.86 (Figure 10B).^[^ ^170^ ^]^ Importantly, the incorporation of His concentration could affect the activation energy of the catalytic reactions and the association toward substrate, and therefore could influent the catalytic ability and specificity of the COF nanozyme. The catalytic activity was significantly enhanced 21.7 folds for His modified COF nanozymes than that of natural enzyme (horse radish peroxidase, HRP). Mesoposous chiral nanomaterials had a large surface area for mass transportation. Tailorable nanochannels and chiral cavity would endow mesoporous chiral nanomaterials with enhanced enantioselectivity. The crystallinity and porosity of the chiral COF made by crystallization of tertiary amines with bis‐aldehydes displayed good catalytic activity and selectivity for asymmetric α‐aminooxylation, aldolysis, and Diels – Alder reactions.^[^ ^243^ ^]^ Three binary and four ternary COFs displayed enantioselectivity as high as 92% and yields as high as 95%. The chiral propargylamine‐linked COFs were produced for highly reusable asymmetric Michael addition reactions between cyclohexanone and 1‐((E)−2nitrovinyl)benzene with an ee value of 96% and yield of 97%.^[^ ^244^ ^]^ The concept of chiral molecules integrated strategy endowed high asymmetric catalysis activity.

To obtain high enantiopurity, most reported thermal asymmetric catalysis was usually performed at low temperatures with limited yield. It was reported that two metal NPs loaded, porphyrin‐containing homochiral COF nanomaterial allowed thermally‐driven asymmetric one‐pot Henry and A3‐coupling reactions (Figure 10C).^[^ ^245^ ^]^ The metal NPs‐COF nanomaterials showed excellent stereoselectivity and high yield due to COF confinement effect and metal NPs catalytic activation. Notably, the visible light irradiation enabled photothermal conversion and provided thermal energy for the asymmetric reactions, in which the COF confinement effect was effectively maintained up to 100 °C. Chiral COF NPs had become the promising candidate nanozymes for efficient enantioselective catalysis, the metal‐free COF had been reported for visible light‐mediated enantioselective photooxidation in water.^[^ ^166^ ^]^ The propylamine‐linked and quaternary ammonium bromide‐modified porphyrin‐COF was prepared for photocatalysis of enantioselective photo‐oxidation of sulfides to sulfoxides in water. The synthesized chiral COF demonstrated a high catalytic yield of 92%, and ee 95%, which could be used as a green, sustainable, and stable catalyst.

**Figure 11:**
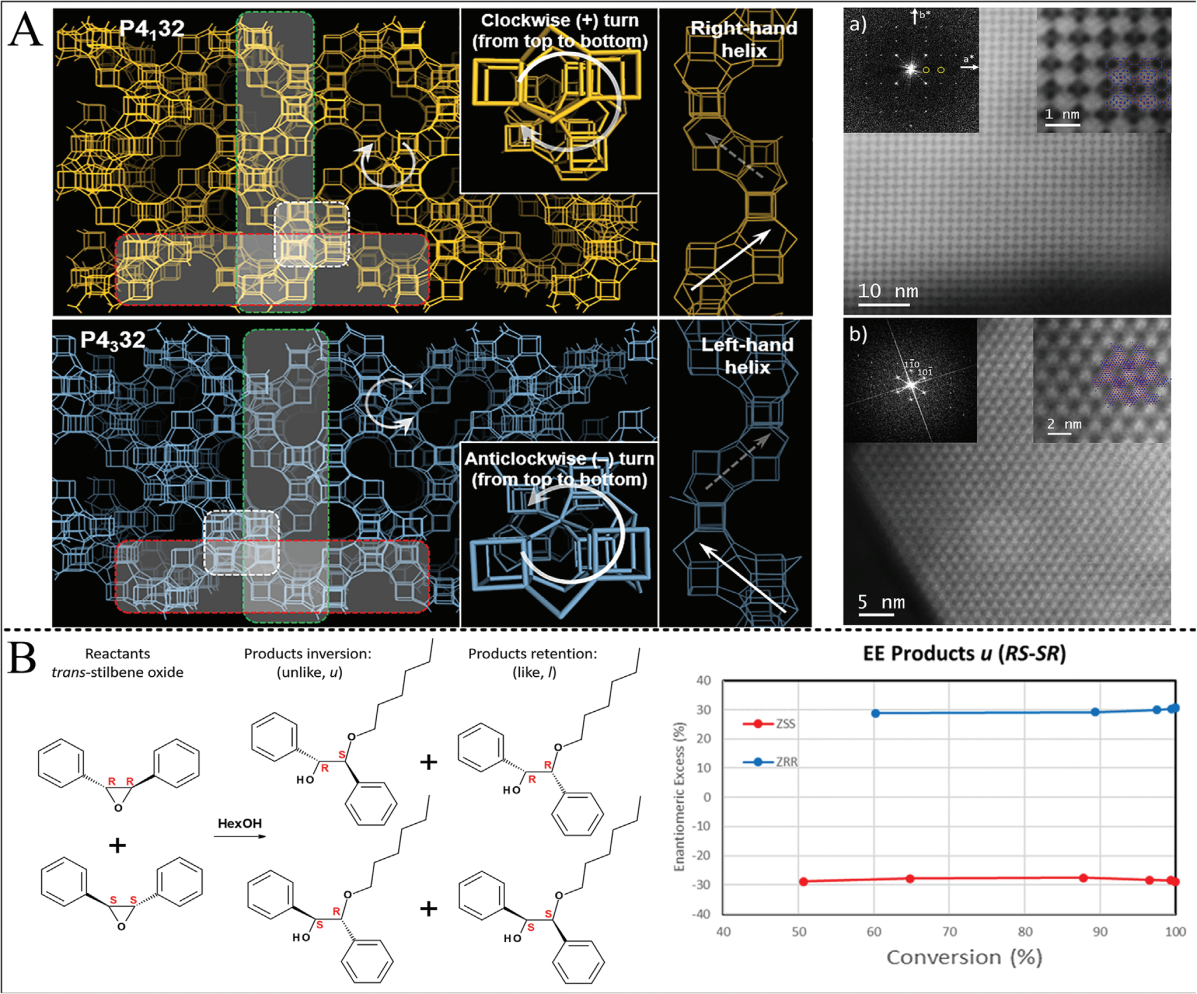
A) Chiral ‐ITV framework with P4_1_32 (top) or P4_3_32 (bottom) enantiomorphic space groups show helicoidal spiral staircase‐like chain units of opposite handedness. The STEM analysis was employed to confirm the framework topology proposed, study the crystallinity of the materials, and provide additional information on the chirality of grupo de tamices moleculares‐3. B) Ring aperture of chiral trans‐stilbene oxide with 1‐hexanol giving inversion products. The enantiomeric excess of reactants increases with conversion, evidencing that one enantiomer reacts faster than the other.^[^ ^247^ ^]^

MOF materials can be further used as templates for other nanomaterials, for example, helical MOF was used as a template to fabricate helical carbon nanorods (HCNRs). HCNRs contained topological pentagonal or heptagonal carbon defects, which had been facilitated for oxygen reduction reactions.^[^ ^177^ ^]^ HCNRs had more positive onset/half‐wave reduction potentials, higher limited current density, and displayed four‐electrons compared to straight carbon nanorods with a two‐electron reduction mechanism. It was revealed that the enhanced oxygen reduction activities were attributed to pentagon/heptagon defects of HCNRs and related carbon materials.^[^ ^246^ ^]^ The work provided a new methodology for the utilization of chiral effects for more effective electrocatalysts. Zeolites as periodic silicate frameworks displayed regular pores and cavities of molecular dimensions that could be used for catalysis. The chiral zeolitic catalysts possessing large pores were obtained using a simple enantiopure organic cation derived from the chiral pool, *N*, *N*‐ethyl‐methylpseudoephedrinium, as the chiral‐inductor agent (**Figure** **11**A).^[^ ^247^ ^]^ It was proven that chiral zeolitic material allowed for enantioselective catalytic reactions with very large substrates, yielding enantiomeric excesses of 30% (Figure 11B). This methodology opens the way for the fabrication of chiral zeolitic materials for catalytic asymmetric synthesis of chiral pharmaceutical compounds.

### 3.6 Chiral Assembled Nanostructures‐Based Catalysis

Bio‐inspired NPs chiral assemblies have received great attention for their potential catalysis applications, including enantioselective catalysis and biological macromolecule catalysis, etc.^[^ ^24^, ^186^ ^]^ In comparison to monodispersed chiral NPs, chiral NP assemblies generally exhibited stronger chiroplasmonic signals and showed potential prospects for in‐site biocatalysis applications.^[^ ^57^, ^227^ ^]^

**Figure 12:**
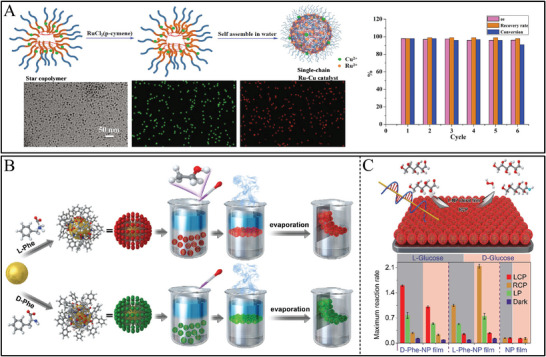
A) Illustration of star‐like Ru‐Cu catalyst and the assembly with corresponding TEM mapping displayed circulation catalytic performance of Cat‐6 in asymmetric transfer hydrogenation of acetophenone.^[^ ^85^ ^]^ B) Schematic illustration of the fabrication of chiral Au NP films by utilizing Phe enantiomers as ligands. C) Photocatalytic activity evaluation of D‐Phe‐NP film or L‐Phe‐NP film for oxidation of glucose enantiomer.^[^ ^188^ ^]^

It was reported that the star‐shaped thermal responsive and catalytical active polymer‐Ru/diamine nanostructures were synthesized by atom transfer radical polymerization (**Figure** **12**A).^[^ ^85^ ^]^ The Ru‐Cu catalyst and Cu^2+^ were polymerized through self‐catalysis of chiral monomers and polymers, in which the fabricated star polymer exhibited a chiral amplification effect. The complex Ru‐Cu nanostructures showed better catalytic activity and selectivity than the single Ru catalyst in aqueous asymmetric catalysis of acetophenone transfer hydrogenation. This work presented a candidate, green, and cost‐effective method for the synthesis of chiral bimetallic nanostructures to imitate high‐efficient enzyme catalysts. Different from chiral assembly in solution, sold‐like chiral NP film state assemblies had emerged and showed intensive and enhanced chiral signals. Phe enantiomers functionalized Au NPs were prepared and assembled into chiral NP films in the interface of hexane and water (Figure 2B).^[^ ^188^ ^]^ The chiral responses of NP films exhibited two CD signals at 520 and 732 nm, in which the intensity of chiral responses could be adjusted by the concentration and stereochemistry of Phe. It was studied that D‐Phe modified chiral NP films showed high catalytic activity toward L‐glucose under left circularly polarized light, and the conversion of L‐glucose reached up to 93% (Figure 12B,C). Meanwhile, L‐Phe modified chiral NP films showed high catalytic activity toward D‐glucose under right circularly polarized light, and the conversion of D‐glucose reached up to 92%. The conversion of glucose enantiomers could be controlled through the handedness of polarized light. This study paved the way for the preparation of solid‐like chiral NP films with unique chiral recognition and switched CD responses in the applications of enantioselective catalysis.

**Figure 13:**
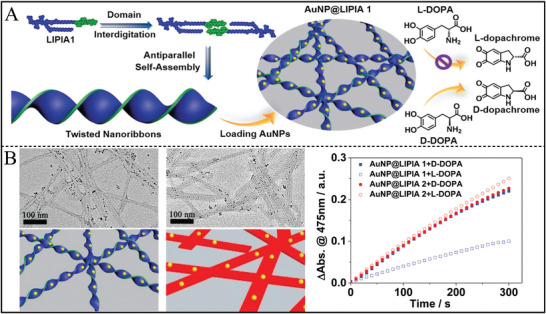
A) Schematic illustration of the design of the chiral scaffolds for gold nanoparticles–lipid‐inspired peptide‐interdigitating amphiphiles by grafting an extended peptide domain to one alkyl tail of lipids either at the N‐ or C‐terminus of the domains. B) TEM images and schematic representation of supramolecular nanozymes. The changes of the UV/vis absorption intensity of the oxidation of L‐ or D‐DOPA as a function of time within the initial period.^[^ ^248^ ^]^

The self‐assembly of biomolecules into highly ordered chiral complex functional structures could provide new insights into the vital role of chirality in catalysis. Based on the peptide‐interdigitating strategy, the controllable assembly of lipid‐inspired amphiphiles into robust twisted nanoribbons was realized. Based on antiparallel or parallel β‐sheet hydrogen bonds interaction, peptide interdigitation induced the amphiphiles' self‐assembly into twisted or flat nanoribbons (**Figure** **13**A,B).^[^ ^248^ ^]^ It was found that the amphiphile assemblies containing N‐terminus‐connected domains allowed for engineering robust chiral nanostructures. The Au NPs were then integrated into the twisted nanoribbons as supramolecular nanozymes exhibiting high asymmetric catalysis activity and enantioselectivity for 3,4‐dihyroxy‐phenylalanine oxidation. The reliable strategies for rational and precise assembly of peptide‐based chiral nanostructures for catalysis are challenging.

### 3.7 Chiral Assembled Supramolecules‐Based Catalysis

Biomolecule chirality was deemed as an essential for life, it was challenging to obtain biomimetic homochirality in supramolecular assembly. The single‐walled supramolecular nanotube catalyst assembled from L‐glutamic acid terminated bolaamphiphile and coordinated metal ions demonstrated significant reactivity and enantioselectivity, in which Bi(III)‐helical nanotube can catalyze the asymmetric Mukaiyama‐Aldol reaction in an aqueous system with enantioselectivity of 97% ee, while Cu(II)‐helical nanotube can catalyze the asymmetric Diels‐Alder reaction with up to 91% ee.^[^ ^196^ ^]^ The supramolecular nanotube‐aligned multi‐catalytic sites and stereochemical selectivity enabled good catalytic performance. The ferrocene‐diphenylalanine with divalent copper ions (Cu^2+^) was assembled through coordination into metal‐peptide hierarchical spiral architectures. The metal‐peptide assembled architecture consisted of helically organized nanofibers that correlated to the logarithmic spirals.^[^ ^43^ ^]^ The porous metal‐peptide assemblies owed abundant ferrocene‐diphenylalanine and Cu^2+^ active sites that exhibited catalytic activity ≈6 times than natural laccase as for decolorization reaction. The assembled peptide enantiomers with higher ee values provided a new strategy for the construction of biomimetic nanozymes. The 3D right‐handed helical assembled nanostructures by polymers, D‐ or L‐ Phe chiral centers and lipase generated enhanced catalytic activity of lipase as high as 10‐fold for catalyzing 4‐nitrophenyl palmitate to nitrophenol and 1.4‐fold for catalyzing lipids to triglycerides in living cells compared to achiral supramolecular.^[^ ^199^ ^]^ The chiral supramolecular may provide a suitable methodology with high catalytic efficiency, storage stability, and efficient recyclability. Alternatively, the self‐assembly of amphiphilic dipeptide with homo centers showed that both the chirality of the supramolecular fiber and the L‐Pro chiral catalytic site was important for asymmetric catalysis.^[^ ^200^ ^]^ The homochiral centers resulted in more efficient catalysis with high yields (up to 97%), anti‐diastereoselectivity (up to 99%), and excellent enantioselectivity (up to >99%). The amino acids or peptide‐interdigitated chiral nanostructures allowed for enhanced catalytic performance and endowed potential strategy for advanced biomimetic catalysis systems.

The supramolecular chiral assemblies were also important as biomimetic nanozymes in which chirality could originate from integrated chiral molecules or assembled chiral geometries.^[^ ^249^, ^250^, ^251^ ^]^ Based on the polymer supramolecular chirality to enhance catalytic efficiency, control over enantioselectivity was gaining increasing interest.^[^ ^252^ ^]^ The assembled supramolecular chiral nanostructures were demonstrated as nanocages, nanotubes, nanorods, micelles, vesicles, and so on with asymmetric catalysis activity.^[^ ^253^ ^]^ For example. the supramolecular strategy encoded chiral macrocyclic multifarane[3,3] displayed asymmetric hydrogenation of 4‐methylumbelliferone in electrochemical reduction and produced L‐7‐hydroxy‐4‐methylchroman2‐one product with a yield of 65% and enantioselectivity up to >99%, which also exhibited high stability and repeatability.^[^ ^250^ ^]^ Based on reversible addition‐fragmentation chain transfer polymerization, the water‐soluble single‐chain polymeric NPs consisting of CO_2_‐switchable copolymers were synthesized by copolymerization of amidine derivatives with hydrophobic chiral salen Fe^III^ monomers that displayed asymmetric sulfa‐Michael addition.^[^ ^254^ ^]^ The stereoselectivity of the chiral supramolecular catalyst is greatly improved when encased in a supramolecular non‐chiral cage during converts styrene derivatives into aldehyde products with much higher chiral induction than the nonencapsulated catalyst with selectivity of 71 ± 3% ee.^[^ ^209^ ^]^ The result demonstrated that structural constraints can impart stereoselectivities.

The catalysis activity of different geometrical supramolecular nanostructures could provide a candidate methodology for the fabrication of biomimetic chiral nanozymes. The coordination‐driven self‐assembled supramolecular chiral metallic triangles through chiral 3,3′‐dipyridyl substituted BINOL donor displayed activity for asymmetric conjugate addition of a series of α,β‐unsaturated ketones with trans‐styrylboronic acids in acceptable yields (40–98%) and enantioselectivities (87–96% ee).^[^ ^211^ ^]^ Importantly, the helical nanoribbon based on benzoic acid appended achiral benzene‐1,3,5‐tricarboxamide helical supramolecular assembly was fabricated with well‐controlled geometry, which displayed the Cu^2+^‐catalyzed Diels–Alder reaction^[^ ^249^ ^]^ The conversion was achieved more than 99% with turnover number as 90, and the enantiomeric excess as 46%. Based on the self‐assembly strategy, the supramolecular architectures have been constructed through multiple non‐covalent interactions for developing biomimetic catalysis alternatives to the natural ones,^[^ ^255^ ^]^ which potentially enabled high catalytic selectivity and efficiency. The overall representative chiral nanomaterials employed for catalysis performance are listed in Table 1.

## 4 Chiral Nanomaterials‐Based Catalysis in Living Systems

Even though a great process had been achieved for different chiral nanomaterials in the application of selective catalysis of biomolecules, a giant challenge still existed for the in‐site biocatalytic activity in vivo. Chiral nanomaterials had the advantages of well selectivity and biocompatibility and had become the preferable biocatalysis for in‐site catalytic applications in living systems.

Currently, the bioapplications of chiral nanomaterials have been extended for in‐site biocatalysis in living systems. In particular, ROS played a pivotal role in adjusting the physiological functions of organisms, and not only became the biomarkers in cancer cells, but also could be used for cancer treatment with a large content. Firstly, chiral nanomaterials demonstrated chiral recognition in living systems enabling great potential for catalysis in vivo. For example, chiral mSiO_2_ nanospheres with molecular‐scale‐like chirality demonstrated good recognition of biomolecule β‐amyloid protein (Aβ42) in living systems that identified the potential for in vivo catalysis.^[^ ^257^ ^]^ Semiconductors were applied as catalysts for the in‐site biocatalysis in living systems. Chiral semiconductors were mainly reported and had the capability of ROS generation for biocatalysis applications of senescent cell elimination, the prevention of β‐amyloidopathy and Hepatitis B virus (HBV) infection, and the treatment of venous thromboembolism (VTE), as well as the amelioration of Parkinson's disease.

Chiral Pen functionalized Cu_x_Co_y_S supraparticles with the diameters of 75.0 ± 8.0 nm was fabricated and possessed wide absorption from 400 nm to 900 nm, which could induce the production of high energy electrons under the excitation of 808 nm.^[^ ^128^ ^]^ In comparison to L‐pen modified Cu_x_Co_y_S supraparticles, D‐pen modified Cu_x_Co_y_S supraparticles had a better association with cell membranes due to the combination between D‐pen and the lipids or proteins. Therefore, D‐pen‐modified Cu_x_Co_y_S supraparticles were used as photocatalysts for the modification of the antibody of beta 2 MG, in order to eliminate the senescent cells.^[^ ^127^ ^]^ Chiral D‐pen‐modified Cu_x_Co_y_S supraparticles displayed 2.5‐fold higher internalized ability than L‐pen‐modified Cu_x_Co_y_S supraparticles. D‐pen‐modified Cu_x_Co_y_S supraparticles induced the generation of caspase‐3, and further induced the apoptosis of senescent cells. The synergistic effect from high ROS levels and magnetic treatment showed better removal efficiency of senescent cells. Multifunctional chiral nanoassemblies were further fabricated for the clearance of senescent cells. DNA‐driven chiral Au NP‐Upconversion nanoparticle (UCNP) nanoassemblies were constructed and demonstrated the ability to recognize senescent cells.^[^ ^258^ ^]^ UCNPs modified by granzyme B and CFDEVDK‐Cy5.5 were obtained and resulted in the apoptosis of senescent cells under the activation of caspases. The spiked chiral Au@AgAu CS NRs with multiple and strong hot spots on the ends of tips were designed for the generation of intensive plasmonic coupling and the generation of much more ROS.^[^ ^259^ ^]^ Au@AgAu core satellite NRs assemblies with triphenylphosphonium were able to combine mitochondria in senescent cells. Upon the illumination by 808 nm light, the chiral Au@AgAu core satellite produced enhanced ROS, which was ≈2.6 folds higher than that of achiral Au NPs. The high ROS concentration induced the destruction of mitochondria and the apoptosis of senescent cells under low NIR light power.

**Figure 14:**
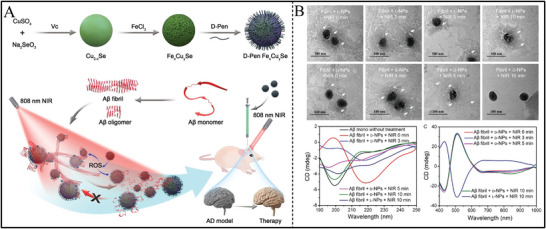
Schematic illustration A) and results of the penicillamine‐modified Fe_x_Cu_y_Se nanoparticles for the inhibition and disassembly of Aβ42 aggregation and mitigation of potential neurotoxicity in an AD mice model (B).^[^ ^123^ ^]^

Chiral nanomaterials generated ROS under light irradiation and had been successfully achieved the elimination of senescent cells. Another exciting application is protocols for the prevention of β‐amyloidopathy in vivo by using chiral nanomaterials depending on the association between chiral ligands and pentapeptide from Aβ42 (**Figure** **14**A). The chiral Fe_x_Cu_y_Se NPs functionalized with D‐ and L‐Pen molecules showed higher association toward the pentapeptide of Aβ42 occurred for D‐Pen functionalized Fe_x_Cu_y_Se NPs than L‐Pen‐modified Fe_x_Cu_y_Se NPs. Therefore, D‐Pen‐modified Fe_x_Cu_y_Se NPs induced the dissociation of Aβ42 fibrils, in which the morphology changed from a dense state to looser monomers (Figure 14B).^[^ ^123^ ^]^ It was due to that D‐Pen‐modified Fe_x_Cu_y_Se NPs resulted in the generation of ^1^O_2_ and ·OH upon the irradiation of 808 nm light, resulting in catalysis disaggregation of Aβ fibrils. In comparison to chiral Cu_2‐x_Se NPs, chiral Fe_x_Cu_y_Se NPs could produce more amounts of ROS upon the excitation of an 808 nm light source, reducing the fibrillation of monomers. Chiral nanomaterials engineered ROS generation was also applied for the disease treatment, for example, venous thromboembolism treatment and the prevention of HBV infection. Antiplatelet therapy and anticoagulant therapy were usually used for traditional medical treatment but had side effects on the body.

Chiral nanomaterials have great potential for in vivo viral reduction control and infection. Based on specific binding between the tripeptide (Pen‐Phe‐Trp) on chiral Cu_2_S NPs surface and the functional domain from phenylalanine_23_ (F_23_) to leucine_30_ (L_30_) of HBcAg primary sequence, chiral L‐Cu_2_S NPs showed better cleavage ability than D‐Cu_2_S NPs.^[^ ^131^ ^]^ By 808 nm laser excitation, chiral L‐Cu_2_S NPs generated more ROS that was 1.5 folds higher than that of D‐Cu_2_S NPs. A large amount of ROS was used to efficiently cut the sites between amino acid residues F_24_ and proline_25_ (P_25_). About 95% HBcAg was successfully reduced in the cell, and ≈93% HBV surface antigen and 86% HBV DNA were reduced. Chiral Cys enantiomers integrated Co_3_O_4_ SPs with an average size of 165 nm were fabricated for thromboembolism treatments.^[^ ^260^ ^]^ Chiral Co_3_O_4_ supraparticles showed mirror and wide chiral signals from 190 nm to 800 nm with maximum g‐factor reached up to 2 × 10^−2^. The intensive CD responses originated from surface lattice distortions. It was found that D‐Co_3_O_4_ supraparticles exhibited better thrombolytic efficiency due to the more amounts of ROS generation, in comparison to L‐Co_3_O_4_ SPs. Additionally, the electromagnetic field promoted ROS production due to the paramagnetic properties of chiral Co_3_O_4_. D‐Co_3_O_4_ SPs exhibited better thromboembolic therapy and enhanced the survival rate up to 70% in mice. Chiral nanomaterials engineered treatments enhanced the therapeutic effects and reduced the use of traditional drugs with side effects.

Amino acids not only endowed nanomaterials with unique chiral signals and recognition ability but also could regulate the morphology of chiral nanomaterials for anti‐oxidation applications. Cu_x_O NPs showed good biocompatibility and multiple mimetic enzyme properties of GSH peroxidase, superoxide dismutase, peroxidase, and catalase, and displayed potential applications in vitro and in vivo. In order to enhance mimicking enzyme behaviors, the structures of Cu_x_O NPs were adjusted by changing different amino acids as chiral ligands, including Tyr, Asp, Glu, and Phe. In particular, ellipsoid Cu_x_O NPs were obtained in the presence of Tyr, Asp, or Glu ligands. The porous Cu_x_O NPs exhibited superoxide dismutase‐like activity as a scavenger of O_2_^·−^, and displayed catalase‐like activity for decomposition of H_2_O_2_ to H_2_O and O_2_, as well as showed GSH peroxidase‐like behaviors for the consume of H_2_O_2_ and the oxidization from GSH to oxidized GSH.^[^ ^107^ ^]^ The chirality and multiple enzyme‐mimicking properties enabled CuxO NPs with enhanced ROS scavenging in tissues, which not only avoided the oxidative stress of Parkinson's disease but also reduced ROS‐mediated cell apoptosis. Recently, GSH‐modified chiral Se NPs were fabricated with intensive CD signals. Owing to the strong binding between L‐GSH and cell membrane, L‐GSH‐modified Se NPs could be easily accumulated in the tissues and D‐GSH functionalized nanomaterials were easily accumulated in living cells. The L‐GSH Se NPs became a promising agent for the antioxidant in tissues, while D‐GSH Se NPs could be used for reducing oxidative stress and accelerating the autophagy process. DNA‐driven UCNP‐centered Yolk‐Shell NP structures were designed, in which FGFE peptide (Cys‐Phe‐Gly‐Phe‐Thr) was functionalized on the surface of the nanoparticle. In order to enhance the autophagy effects, chiral GSH enantiomers were modified on the surface of assembled structures.^[^ ^261^ ^]^ It was found that D‐GSH functionalized structures showed more efficient autophagy activity than L‐GSH functionalized structures. This was due to the higher accumulation of D‐GSH functionalized structures in living cells, which further improved oxidative stress and accelerated the autophagy process. Chiral metamaterials showed immense potential applications for the in‐site monitoring of the autophagy process in vivo.

**Figure 15:**
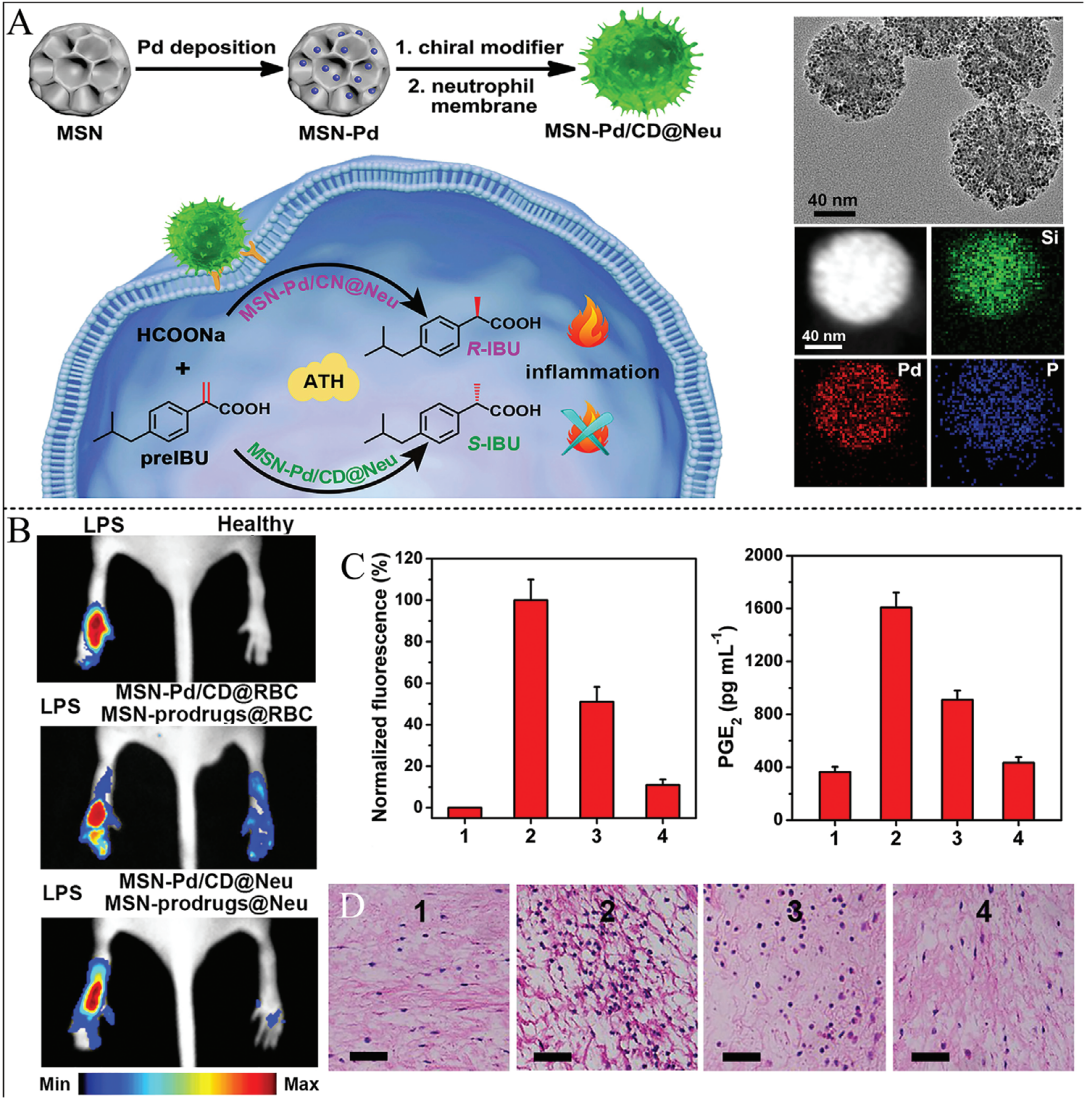
A) The preparation and intracellular catalysis activity of mesoporous silica‐Pd nanoparticles and structure characterizations. B–D) Targeted asymmetric transfer hydrogenation reaction catalyzed by mesoporous silica‐Pd nanoparticles for vivo anti‐inflammation. B) ROS imaging of lipopolysaccharide‐induced inflamed paws. C) Normalization of corresponding fluorescence intensities of ROS and prostaglandin E_2_ level. D) Staining images of inflamed paws.^[^ ^263^ ^]^

Alternatively, chiral transition metal NPs with excellent enantioselectivity showed immense potential prospects for catalysis in living systems. Supramolecular complexes of azobenzene and β‐cyclodextrin modified chiral mesoporous silica‐Pd NPs were also designed and served as light‐controlled bioorthogonal catalysts.^[^ ^262^ ^]^ The catalytic performances were controlled by light‐driven morphology changes by mimicking the allosteric regulation mechanism of bio‐enzymes. Bioorthogonal catalysis was performed for the preparation of chiral drugs in living systems with the aid of chiral nanomaterials, which was of vital importance in the clinic. In comparison to natural enzymes, metal oxide‐based nanozymes possessed superior catalytic performances, which could efficiently catalyze chemical transformations. Chiral Pd catalysts were prepared by the reduction of biocompatible sodium formate and were modified on mesoporous silica NPs (**Figure** **15**).^[^ ^263^ ^]^ The sizes of NPs were ≈98 nm, the pore sizes were ≈10.6 nm, and the sizes of as‐formed Pd NPs were 2.9 nm (Figure 15A). As fabricated mesoporous silica‐Pd NPs were functionalized by cinchonidine, cinchonine, quinidine, and quinine that endowed enantioselectivity. The neutrophil‐membrane functionalized chiral mesoporous silica‐Pd NPs achieved the inflammation site‐targeted chiral drug synthesis in living cells and exhibited inflammation targeted capability and enantioselectivity in the anti‐inflammatory reaction (Figure 15B–D). The chiral ibuprofen was site‐selective synthesized, which could in‐site suppress inflammation in vivo. The purity of the activated neutrophils reached up to 90%. The assembled chiral supramolecule nanostructures could also be applied in living cells, for example, the enantiopure polythiophene NPs displayed enantioselective intracellular antimicrobial activity.^[^ ^264^ ^]^ Asymmetric transfer hydrogenation reactions were also performed in cancer cells for the enantioselective reduction of pyruvate by using chiral half‐sandwich organometallic Os(II) arene sulfonyl diamine complexes.^[^ ^265^ ^]^ Pyruvate as the intermediate in metabolic pathways was successfully reduced to lactate in human cancer cells by using non‐toxic sodium formate as a hydride source. The production of reductive stress in cancer cells enabled transfer‐hydrogenation catalysts to be an alternative approach for the applications of cancer therapy.

## 5 Summary and Outlook

In conclusion, recent advances and future perspectives regarding chiral nanomaterials‐based catalysis were systematically summarized in the review. The fabrication of multiscale chiral nanomaterials and the strategies for the enhancement of chiroptical properties were proposed. The chirality‐enhanced catalysis applications in the fields of enantioselective catalysis, biological macromolecule catalysis, and in‐site biocatalysis in living systems and so on were discussed and compared. Even though great progress has been obtained for the chiral nanomaterials involved in catalysis applications, there are still some challenges in future trends. From the fabrication perspective, enhanced chiroptical properties and high reproducibility are needed for the fabrication of chiral nanomaterials. Future advances in solid‐like chiral nanomaterial preparation are still challenging for practical catalysis applications. This entails the exploration of facile synthesis routes in mild conditions and the use of inexpensive precursors for laboratory‐scale batch production, as well as the reduced usage of heavy metals of Cd, Hg, Pb, and novel metals of Au and Ag. Some less toxic Bi‐, Sb‐, Sn‐QDs, and non‐noble metal Cu‐, Al‐ NPs possess superior energy‐harvesting ability and need to be further exploited. Furthermore, water‐stable CsPbX_3_ perovskite QDs have been emerging in light‐harvesting photocatalysis for chirality‐coupled catalysis applications. From the biocatalytic activity perspective, some chiral nanomaterials still show relatively weaker catalytic behaviors than natural enzymes. Opportunities remain in the design of chiral nanomaterials to extend the light response range from ultraviolet (UV)‐visible to NIR wavelength. The wide chiral response enabled the efficient harvesting of light energy for the enhancement of catalytic activity and allowed the utilization of low‐power NIR laser in living systems. Chiral nanomaterials still display lower selectivity toward substrates than natural enzymes. It is necessary to integrate functional groups with nanoparticles during the synthesis process, which could well mimic and even transcend the activity and selectivity of natural enzymes. The advanced electronic microscopes and spectroscopic techniques will help to deepen the understanding of the structure‐property relationship as well as the catalytic mechanisms in virtue, which will motivate the development of novel chiral nanomaterials as efficient catalysts that can exhibit performance superior to enzymes in selective catalysis. Challenges also remain in the area of the design of long‐term durability of chiral catalysts. Moving forward, a systematic understanding of the connection of electromagnetic field and the separation of charge carriers as well as the biocatalytic performances would benefit the establishment of highly efficient chiral nanomaterials for catalysis. From the bioapplication perspective, low‐toxic and non‐toxic chiral nanomaterials are increasingly important and should be given more attention. The cellular cytotoxicity would be further reduced with the aid of chiral nanomaterials. Chiral nanomaterials widely focused on the applications in redox reactions and hydrolytic reactions, and still show immense potential prospects for the applications of more biochemical reactions.

## Conflict of Interest

The authors declare no conflict of interest.

## References

[1] W. Ma , L. Xu , A. F. de Moura , X. Wu , H. Kuang , C. Xu , N. A. Kotov , Chem. Rev. 2017, 117, 8041. 28426196 10.1021/acs.chemrev.6b00755

[2] L. Xiao , T. An , L. Wang , X. Xu , H. Sun , Nano Today 2020, 30, 100824.

[3] S. W. Im , H. Y. Ahn , R. M. Kim , N. H. Cho , H. Kim , Y. C. Lim , H. E. Lee , K. T. Nam , Adv. Mater. 2020, 32, 1905758.

[4] S. Zhu , M. Sun , Appl. Spectrosc. Rev. 2020, 56, 553.

[5] J. Fan , N. A. Kotov , Adv. Mater. 2020, 32, 1906738. 10.1002/adma.201906738 32500963

[6] L. Ruiyi , W. Xiaobo , P. Yuanfeng , X. Pengwu , Z. Haiyan , L. Zaijun , S. Xiulan , Catal. Sci. Technol. 2022, 12, 2097.

[7] Y. Ma , Z. Cao , J. Hao , J. Zhou , Z. Yang , Y. Yang , J. Wei , J. Phys. Chem. C 2020, 124, 24306.

[8] L. Ma , Y. Liu , C. Han , A. Movsesyan , P. Li , H. Li , P. Tang , Y. Yuan , S. Jiang , W. Ni , H. Yan , A. O. Govorov , Z. M. Wang , X. Lan , Nano Lett. 2022, 22, 4784. 35649094 10.1021/acs.nanolett.2c01047

[9] W. Wu , Y. Battie , V. Lemaire , G. Decher , M. Pauly , Nano Lett. 2021, 21, 8298. 34546067 10.1021/acs.nanolett.1c02812

[10] R. F. Zhang , Y. L. Zhou , X. Y. Yan , K. L. Fan , Microchim. Acta 2019, 186, 782. 10.1007/s00604-019-3922-7 31729634

[11] Y. Xia , Y. Zhou , Z. Tang , Nanoscale 2011, 3, 1374. 21301709 10.1039/c0nr00903b

[12] L. Marchetti , M. Levine , C. Biomimetic , ACS Catal. 2011, 1, 1090.

[13] K. Y. Wang , J. Zhang , Y. C. Hsu , H. Lin , Z. Han , J. Pang , Z. Yang , R. R. Liang , W. Shi , H. C. Zhou , Chem. Rev. 2023, 123, 5347. 37043332 10.1021/acs.chemrev.2c00879 PMC10853941

[14] F. Liu , Z. Shi , W. Su , J. Wu , Biotechnol. Biotec. Eq. 2022, 36, 118.

[15] D. Liang , Y. Wang , K. Qian , Interdiscip. Med. 2023, 1, e20230020.

[16] X. Qiu , Y. Zhang , Y. Zhu , C. Long , L. Su , S. Liu , Z. Tang , Adv. Mater. 2021, 33, 2001731. 10.1002/adma.202001731 32672886

[17] H. Zhang , S. Li , A. Qu , C. Hao , M. Sun , L. Xu , C. Xu , H. Kuang , Chem. Sci. 2020, 11, 12937. 34094483 10.1039/d0sc03245j PMC8163208

[18] Q. Yang , Q. Xu , H. L. Jiang , Chem. Soc. Rev. 2017, 46, 4774. 28621344 10.1039/c6cs00724d

[19] S. Y. Cao , F. Ye , N. N. Zhang , Y. L. Guo , Y. Guo , L. Wang , S. Dai , W. C. Zhan , Rare Met. 2023, 42, 165.

[20] Y. Zhao , Y. Yang , Y. Luo , X. Yang , M. Li , Q. Song , ACS Appl. Mater. Interfaces 2015, 7, 21780. 26381109 10.1021/acsami.5b07804

[21] F. Zheng , W. Ke , L. Shi , H. Liu , Y. Zhao , Anal. Chem. 2019, 91, 11812. 31424931 10.1021/acs.analchem.9b02469

[22] H. Yin , K. Xing , Y. Zhang , D. Dissanayake , Z. Lu , H. Zhao , Z. Zeng , J. H. Yun , D. C. Qi , Z. Yin , Chem. Soc. Rev. 2021, 50, 6423. 34100047 10.1039/d0cs01146k

[23] S. Srivastava , A. Santos , K. Critchley , K. S. Kim , P. Podsiadlo , K. Sun , J. Lee , C. L. Xu , G. D. Lilly , S. C. Glotzer , N. A. Kotov , Science 2010, 327, 1355. 20150443 10.1126/science.1177218

[24] M. Arabi , A. Ostovan , Y. Wang , R. Mei , L. Fu , J. Li , X. Wang , L. Chen , Nat. Commun. 2022, 13, 5757. 36180485 10.1038/s41467-022-33448-w PMC9525700

[25] K. Chen , T. Jiao , J. Li , D. Han , R. Wang , G. Tian , Q. Peng , Langmuir 2019, 35, 3337. 30730141 10.1021/acs.langmuir.9b00014

[26] Y. Li , J. Liu , Mater. Horiz. 2021, 8, 336. 34821258 10.1039/d0mh01393e

[27] X. Chen , Z. Jia , Y. Wen , Y. Huang , X. Yuan , Y. Chen , Y. Liu , J. Liu , Acta Biomater. 2022, 151, 537. 35981687 10.1016/j.actbio.2022.08.020

[28] X. Zhao , S. Q. Zang , X. Chen , Chem. Soc. Rev. 2020, 49, 2481. 32176233 10.1039/d0cs00093k

[29] S. Mondal , F. Dumur , D. Gigmes , M. P. Sibi , M. P. Bertrand , M. Nechab , Chem. Rev. 2022, 122, 5842. 35073048 10.1021/acs.chemrev.1c00582

[30] Z. Pei , H. Lei , L. Cheng , Chem. Soc. Rev. 2023, 52, 2031. 36633202 10.1039/d2cs00352j

[31] X. Du , R. Jin , ACS Nano 2019, 13, 7383. 31246403 10.1021/acsnano.9b04533

[32] L. V. Besteiro , A. Movsesyan , O. Avalos‐Ovando , S. Lee , E. Cortes , M. A. Correa‐Duarte , Z. M. Wang , A. O. Govorov , Nano Lett. 2021, 21, 10315. 34860527 10.1021/acs.nanolett.1c03503 PMC8704195

[33] R. Parella , Naveen , S. A. Babu , Catal. Commun. 2012, 29, 118.

[34] A. N. Murashkevich , O. V. Fedorova , T. F. Kuznetsova , O. A. Alisienok , Y. A. Titova , O. V. Koryakova , G. L. Rusinov , J. Sol‐Gel Sci. Techn. 2023, 108, 298.

[35] M. Kim , J. Kim , H. J. Lee , H. Kim , K. T. Nam , D. H. Kim , ACS Nano 2023, 17, 7611. 37011403 10.1021/acsnano.3c00047

[36] J. Hao , Y. Li , J. Miao , R. Liu , J. Li , H. Liu , Q. Wang , H. Liu , M. H. Delville , T. He , K. Wang , X. Zhu , J. Cheng , ACS Nano 2020, 14, 10346. 32806030 10.1021/acsnano.0c03909

[37] Y. Gu , Y. Jiang , J. Chen , C. Gao , L. Feng , J. Wu , L. Zhao , Opt. Mater. 2022, 132, 112787.

[38] Y. N. Yue , S. Zeng , H. Wang , S. Wang , H. Wang , J. X. Lu , ACS Appl. Mater. Interfaces 2018, 10, 23055. 29916236 10.1021/acsami.8b04589

[39] M. Zhang , X. Fan , X. Du , Y. Ma , X. Wang , H. Huang , Y. Liu , Y. Li , Z. Kang , Nano Res. 2023, 16, 8929.

[40] Y. Tian , Y. Guo , X. Dong , X. Wan , K. H. Cheng , R. Chang , S. Li , X. Cao , Y. T. Chan , A. C. H. Sue , Nat. Synth. 2023, 2, 395.

[41] H. Zhang , L. L. Lou , K. Yu , S. Liu , Small 2021, 17, e2005686. 33734597 10.1002/smll.202005686

[42] P. Liu , Y. Battie , M. Decossas , S. Tan , E. Pouget , Y. Okazaki , T. Sagawa , R. Oda , ACS Nano 2021, 15, 16411. 34617734 10.1021/acsnano.1c05819

[43] J. Liu , J. Zhang , L. Zhang , Y. Wang , H. Wei , Y. Shen , J. Min , X. Rong , W. Qi , R. Su , Z. He , Sci. Chi. Chem. 2022, 66, 228.

[44] X. Gao , X. Chen , Z. H. Li , H. He , ACS Catal. 2019, 9, 6100.

[45] I. Cano , M. J. L. Tschan , L. M. Martínez‐Prieto , K. Philippot , B. Chaudret , P. W. N. M. van Leeuwen , Catal. Sci. Technol. 2016, 6, 3758.

[46] S. Liu , Y. He , Y. Liu , S. Wang , Y. Jian , B. Li , C. Xu , Chem. Commun. 2021, 57, 3680. 10.1039/d1cc00755f 33725076

[47] X. W. Dong , Y. Yang , J. X. Che , J. Zuo , X. H. Li , L. Gao , Y. Z. Hu , X. Y. Liu , Green Chem. 2018, 20, 4085.

[48] H. Zhang , H. He , X. M. Jiang , Z. N. Xia , W. L. Wei , ACS Appl. Mater. Interfaces 2018, 10, 30680. 30113158 10.1021/acsami.8b10594

[49] S. Li , M. Veksler , Z. Xu , L. Xu , C. Xu , N. A. Kotov , ACS Energy Lett. 2021, 6, 1405.

[50] J. Y. Wangwang Zheng , Y. Zhao , Anal. Chem. 2021, 93, 13080. 34523913 10.1021/acs.analchem.1c03182

[51] F. Zheng , W. Ke , L. Shi , H. Liu , Y. Zhao , Anal. Chem. 2019, 91, 11812. 31424931 10.1021/acs.analchem.9b02469

[52] W. Ma , C. Hao , M. Sun , L. Xu , C. Xu , H. Kuang , Mater. Horiz. 2018, 5, 141.

[53] G. Zheng , J. He , V. Kumar , S. Wang , I. Pastoriza‐Santos , J. Perez‐Juste , L. M. Liz‐Marzan , K. Y. Wong , Chem. Soc. Rev. 2021, 50, 3738. 33586721 10.1039/c9cs00765b

[54] H. E. Lee , H. Y. Ahn , J. Mun , Y. Y. Lee , M. Kim , N. H. Cho , K. Chang , W. S. Kim , J. Rho , K. T. Nam , Nature 2018, 556, 360. 29670265 10.1038/s41586-018-0034-1

[55] X. Zhang , Y. Xu , C. Valenzuela , X. Zhang , L. Wang , W. Feng , Q. Li , Light Sci. Appl. 2022, 11, 223. 35835737 10.1038/s41377-022-00913-6 PMC9283403

[56] N. Baig , I. Kammakakam , W. Falath , Mater. Adv. 2021, 2, 1821.

[57] O. S. Ávalos‐Ovando , E. Yazmin , A. K. Movsesyan , X. Tian , P. B. Yu , L. V. Khorashad , L. K. Okamoto , J. M. Hiromi , C. D. Slocik , A. Miguel , M. L. Comesaña‐Hermo , T. Liedl , Z. Wang , G. Markovich , S. Burger , A. O. Govorov , ACS Photonics 2022, 9, 2219.

[58] Q. Ma , C. Cheng , D. Luo , J. Qiao , L. Qi , ACS Appl. Nano Mater 2023, 6, 1676. 10.1021/acsabm.3c00118 37014970

[59] J. M. Slocik , P. B. Dennis , A. O. Govorov , N. M. Bedford , Y. Ren , R. R. Naik , ACS Biomater. Sci. Eng. 2020, 6, 2612. 33463283 10.1021/acsbiomaterials.9b00933

[60] P. Zhan , Z. G. Wang , N. Li , B. Ding , ACS Catal. 2015, 5, 1489.

[61] X. Wu , L. Xu , W. Ma , L. Liu , H. Kuang , W. Yan , L. Wang , C. Xu , Adv. Funct. Mater. 2015, 25, 850.

[62] N. Kowalska , F. Bandalewicz , J. Kowalski , S. Gomez‐Grana , M. Baginski , I. Pastoriza‐Santos , M. Grzelczak , J. Matraszek , J. Perez‐Juste , W. Lewandowski , ACS Appl. Mater. Interfaces 2022, 14, 50013. 36305423 10.1021/acsami.2c11925 PMC9650650

[63] H. Wang , Y. Liu , J. Yu , Y. Luo , L. Wang , T. Yang , B. Raktani , H. Lee , ACS Appl. Mater. Interfaces 2022, 14, 3559. 34982532 10.1021/acsami.1c22191

[64] F. Wu , Y. Tian , X. Luan , X. Lv , F. Li , G. Xu , W. Niu , Nano Lett. 2022, 22, 2915. 35362992 10.1021/acs.nanolett.2c00094

[65] N. H. Cho , Y. B. Kim , Y. Y. Lee , S. W. Im , R. M. Kim , J. W. Kim , S. D. Namgung , H. E. Lee , H. Kim , J. H. Han , H. W. Chung , Y. H. Lee , J. W. Han , K. T. Nam , Nat. Commun. 2022, 13, 3831. 35780141 10.1038/s41467-022-31513-y PMC9250518

[66] X. Q. Liang , Y. Z. Li , Z. Wang , S. S. Zhang , Y. C. Liu , Z. Z. Cao , L. Feng , Z. Y. Gao , Q. W. Xue , C. H. Tung , D. Sun , Nat. Commun. 2021, 12, 4966. 34404784 10.1038/s41467-021-25275-2 PMC8371133

[67] N. A. Kotov , L. M. Liz‐Marzán , Q. Wang , Mater. Adv. 2022, 3, 3677.

[68] W. Wu , M. Pauly , Mater. Adv. 2022, 3, 186.

[69] X. M. Luo , C. H. Gong , F. Pan , Y. Si , J. W. Yuan , M. Asad , X. Y. Dong , S. Q. Zang , T. C. W. Mak , Nat. Commun. 2022, 13, 1177. 35246541 10.1038/s41467-022-28893-6 PMC8897454

[70] X. Zhang , Y. Cao , Z. Huang , S. Zhang , C. Liu , L. Pan , C. Shi , X. Zhang , Y. Zhou , G. Yang , J. Zou , Carbon Energy 2023, 5, e266.

[71] J. Liu , L. Yang , P. Qin , S. Zhang , K. K. L. Yung , Z. Huang , Adv. Mater. 2021, 33, 2005506. 10.1002/adma.202005506 33594700

[72] P. C. Mondal , D. Asthana , R. K. Parashar , S. Jadhav , Mater. Adv. 2021, 2, 7620.

[73] N. Mukherjee , B. Mondal , T. N. Saha , R. Maity , Appl. Organomet. Chem. 2022, 10.1002/aoc.6794.

[74] J. Chen , J. Du , S. M. Li , J. H. Liu , M. Yu , Rare Met. 2023, 42, 3532.

[75] J. Wang , X. Wu , W. Ma , C. Xu , Adv. Func. Mater. 2020, 30, 2000670.

[76] C. Hao , L. Xu , W. Ma , X. Wu , L. Wang , H. Kuang , C. Xu , Adv. Funct. Mater. 2015, 25, 5816.

[77] F. Wu , J. Guo , Y. Huang , K. Liang , L. Jin , J. Li , Deng , R. Jiao , Y. Liu , J. Zhang , W. Zhang , L. Yu , ACS Nano 2021, 15, 2292. 33356158 10.1021/acsnano.0c08274

[78] W. Wang , F. Wu , Y. Zhang , W. Wei , W. Niu , G. Xu , Chem. Commun. 2021, 57, 7390. 10.1039/d1cc01891d 34223840

[79] Y. Wang , W. He , C. H. Li , C. Xia , Y. Yan , C. M. Li , C. Z. Huang , Chem. Commun. 2021, 57, 3211. 10.1039/d0cc07576k 33644788

[80] Y. Xin , K. Yu , L. Zhang , Y. Yang , H. Yuan , H. Li , L. Wang , J. Zeng , Adv. Mater. 2021, 33, 2008145. 10.1002/adma.202008145 34050979

[81] H. E. Lee , R. M. Kim , H. Y. Ahn , Y. Y. Lee , G. H. Byun , S. W. Im , J. Mun , J. Rho , K. T. Nam , Nat. Commun. 2020, 11, 263. 31937767 10.1038/s41467-019-14117-x PMC6959252

[82] L. Xu , X. Wang , W. Wang , M. Sun , W. J. Choi , J. Y. Kim , C. Hao , S. Li , A. Qu , M. Lu , X. Wu , F. M. Colombari , W. R. Gomes , A. L. Blanco , A. F. de Moura , X. Guo , H. Kuang , N. A. Kotov , C. Xu , Nature 2022, 601, 366. 35046606 10.1038/s41586-021-04243-2

[83] X. Wen , S. Wang , R. Liu , R. Duan , S. Hu , T. Jiao , L. Zhang , M. Liu , Small 2022, 18, e2104301. 34825484 10.1002/smll.202104301

[84] S. Butcha , S. Assavapanumat , S. Ittisanronnachai , V. Lapeyre , C. Wattanakit , A. Kuhn , Nat. Commun. 2021, 12, 1314. 33637758 10.1038/s41467-021-21603-8 PMC7910542

[85] X. Li , J. Dong , G. Ma , N. Ma , X. Jia , J. Catal. 2022, 411, 84.

[86] L. Liu , A. Corma , Chem. Rev. 2023, 123, 4855. 36971499 10.1021/acs.chemrev.2c00733 PMC10141355

[87] H. Liu , W. Zheng , Y. Zhao , Y. Zhou , Anal. Chem. 2021, 93, 4944. 33705112 10.1021/acs.analchem.0c05439

[88] Y. Zhang , P. Wang , D. Yu , H. Zhao , X. Lyu , L. Lei , J. Cent. South. Univ. 2022, 29, 2239.

[89] Y. Zhao , W. Ke , J. Shao , F. Zheng , H. Liu , L. Shi , ACS Appl. Mater. Interfaces 2019, 11, 41204. 31588721 10.1021/acsami.9b10398

[90] H. Han , Y. Luo , Y. Jia , N. Hasan , C. Liu , Prog. Nat. Sci‐Mater. 2022, 32, 517.

[91] B. H. Huang , X. Z. Jia , Y. Li , A. Zheng , Y. Sun , Catal. Surv. Asia 2017, 21, 13.

[92] F. Zhang , J. Ai , K. Ding , Y. Duan , L. Han , S. Che , Chem. Commun. 2020, 56, 4848. 10.1039/d0cc00669f 32236248

[93] C. Xu , X. Qu , NPG Asia Mater 2014, 6, e90.

[94] R. Dalpozzo , Green Chem. 2015, 17, 3671.

[95] P. Rai , D. Gupta , Synth. Commun. 2021, 51, 3059.

[96] H. Zhong , B. Zhao , J. Deng , Nanoscale 2021, 13, 11765. 34231630 10.1039/d1nr01939b

[97] L. Gao , K. Fan , X. Yan , Theranostics 2017, 7, 3207. 28900505 10.7150/thno.19738 PMC5595127

[98] Q. Zhang , X. Yang , J. Guan , ACS Appl. Nano Mater 2019, 2, 4681.

[99] O. Gleeson , G. L. Davies , A. Peschiulli , R. Tekoriute , Y. K. Gun'ko , S. J. Connon , Org. Biomol. Chem. 2011, 9, 7929. 21989817 10.1039/c1ob06110k

[100] A. G. Hu , G. T. Yee , W. B. Lin , J. Am. Chem. Soc. 2005, 127, 12486. 16144385 10.1021/ja053881o

[101] E. J. Bealer , K. Kavetsky , S. Dutko , S. Lofland , X. Hu , Int. J. Mol. Sci. 2019, 21, 186. 31888066 10.3390/ijms21010186 PMC6981412

[102] Y. Zhou , W. Wei , F. Cui , Z. Yan , Y. Sun , J. Ren , X. Qu , Chem. Sci. 2020, 11, 11344. 34094377 10.1039/d0sc03082a PMC8162767

[103] K. Mori , Y. Kondo , H. Yamashita , Phys. Chem. Chem. Phys. 2009, 11, 8949. 20449041 10.1039/b910069e

[104] J. Yeom , U. S. Santos , M. Chekini , M. Cha , A. F. de Moura , N. A. Kotov , Science 2018, 359, 309. 29348234 10.1126/science.aao7172

[105] B. Han , X. Gao , J. Lv , Z. Tang , Adv. Mater. 2020, 32, 1801491. 10.1002/adma.201801491 30345582

[106] T. T. Zhuang , Y. Li , X. Gao , M. Wei , F. P. García de Arquer , P. Todorović , J. Tian , G. Li , C. Zhang , X. Li , L. Dong , Y. Song , Y. Lu , X. Yang , L. Zhang , F. Fan , S. O. Kelley , S. H. Yu , Z. Tang , E. H. Sargent , Nat. Nanotech. 2020, 15, 192. 10.1038/s41565-019-0606-8 31959929

[107] C. Hao , A. Qu , L. Xu , M. Sun , H. Zhang , C. Xu , H. Kuang , J. Am. Chem. Soc. 2019, 141, 1091. 30540450 10.1021/jacs.8b11856

[108] S. Jiang , M. Chekini , Z. B. Qu , Y. Wang , A. Yeltik , Y. Liu , A. Kotlyar , T. Zhang , B. Li , H. V. Demir , N. A. Kotov , J. Am. Chem. Soc. 2017, 139, 13701. 28803469 10.1021/jacs.7b01445

[109] B. M. Choudary , K. Jyothi , M. Roy , M. L. Kantam , B. Sreedhar , Adv. Synth. Catal. 2004, 346, 1471.

[110] C. Hao , R. Gao , Y. Li , L. Xu , M. Sun , C. Xu , H. Kuang , Angew. Chem., Int. Ed. 2019, 58, 7371. 10.1002/anie.201902673 30950141

[111] G. Basaran Dindas , D. Y. Koseoglu‐Imer , H. C. Yatmaz , Prog. Nat. Sci‐Mater. 2022, 32, 273.

[112] G. Li , X. Fei , H. Liu , J. Gao , J. Nie , Y. Wang , Z. Tian , C. He , J. L. Wang , C. Ji , D. Oron , G. Yang , ACS Nano 2020, 14, 4196. 32298573 10.1021/acsnano.9b09101 PMC7467813

[113] Z. Cao , J. He , Z. Liu , H. Zhang , B. Chen , ACS Nano 2021, 15, 16255. 34553906 10.1021/acsnano.1c05243

[114] F. P. Garcia de Arquer , D. V. Talapin , V. I. Klimov , Y. Arakawa , M. Bayer , E. H. Sargent , Science 2021, 373, eaaz8541. 34353926 10.1126/science.aaz8541

[115] Y. Liu , H. Li , S. Li , X. Zhang , J. Xiong , F. Jiang , Y. Liu , P. Jiang , ACS Appl. Mater. Interfaces 2021, 13, 60933. 34923825 10.1021/acsami.1c20486

[116] M. Sun , L. Xu , A. Qu , P. Zhao , T. Hao , W. Ma , C. Hao , X. Wen , F. M. Colombari , A. F. de Moura , N. A. Kotov , C. Xu , H. Kuang , Nat. Chem. 2018, 10, 821. 30030537 10.1038/s41557-018-0083-y

[117] S. Jiang , N. A. Kotov , Adv. Mater. 2022, 2108431. 10.1002/adma.202108431 35023219

[118] L. Chen , C. Hao , J. Cai , C. Chen , W. Ma , C. Xu , L. Xu , H. Kuang , Angew. Chem., Int. Ed. 2021, 60, 26276. 10.1002/anie.202112582 34608731

[119] J. Kwon , W. J. Choi , U. Jeong , W. Jung , I. Hwang , K. H. Park , S. G. Ko , S. M. Park , N. A. Kotov , J. Yeom , Nano Converg 2022, 9, 32. 35851425 10.1186/s40580-022-00322-w PMC9294134

[120] M. P. Moloney , Y. K. Gun'ko , J. M. Kelly , Chem. Commun. 2007, 38, 3900. 10.1039/b704636g 17896026

[121] A. Ben‐Moshe , A. O. Govorov , G. Markovich , Angew. Chem., Int. Ed. 2013, 52, 1275. 10.1002/anie.201207489 23233421

[122] Y. Zhou , M. Yang , K. Sun , Z. Tang , N. A. Kotov , J. Am. Chem. Soc. 2010, 132, 6006. 20384329 10.1021/ja906894r

[123] H. Zhang , C. Hao , A. Qu , M. Sun , L. Xu , C. Xu , H. Kuang , Angew. Chem., Int. Ed. 2020, 59, 7131. 10.1002/anie.202002028 32067302

[124] W. Ma , J. Mao , C. Hao , L. Xu , C. Xu , H. Kuang , Appl. Catal., B: Environ. 2019, 245, 691.

[125] M. V. Mukhina , V. G. Maslov , A. V. Baranov , A. V. Fedorov , A. O. Orlova , F. Purcell‐Milton , J. Govan , Y. K. Gun'ko , Nano Lett. 2015, 15, 2844. 25908405 10.1021/nl504439w

[126] K. Varga , S. Tannir , B. E. Haynie , B. M. Leonard , S. V. Dzyuba , J. Kubelka , M. Balaz , ACS Nano 2017, 11, 9846. 28956912 10.1021/acsnano.7b03555

[127] S. Li , M. Sun , C. Hao , A. Qu , X. Wu , L. Xu , C. Xu , H. Kuang , Angew. Chem., Int. Ed. 2020, 59, 13915. 10.1002/anie.202004575 32400008

[128] S. Li , L. Xu , C. Hao , M. Sun , X. Wu , H. Kuang , C. Xu , Angew. Chem., Int. Ed. 2019, 58, 19067. 10.1002/anie.201911770 31612590

[129] X. Guo , N. Xue , M. Zhang , R. Ettelaie , H. Yang , Nat. Commun. 2022, 13, 5935. 36209156 10.1038/s41467-022-33756-1 PMC9547976

[130] S. Li , J. Liu , N. S. Ramesar , H. Heinz , L. Xu , C. Xu , N. A. Kotov , Nat. Commun. 2019, 10, 4826. 31645546 10.1038/s41467-019-12134-4 PMC6811642

[131] X. Guo , M. Sun , R. Gao , A. Qu , C. Chen , C. Xu , H. Kuang , L. Xu , Angew. Chem., Int. Ed. 2021, 60, 13073. 10.1002/anie.202103717 33837622

[132] Y. Ru , L. Ai , T. Jia , X. Liu , S. Lu , Z. Tang , B. Yang , Nano Today 2020, 34, 100953.

[133] B. Kong , T. Yang , F. Cheng , Y. Qian , C. Li , L. Zhan , Y. Li , H. Zou , C. Huang , J. Colloid Interface Sci. 2022, 611, 545. 34971965 10.1016/j.jcis.2021.12.107

[134] G. Han , W. Zhang , L. Li , J. Du , Y. Yan , L. Geng , Y. Tong , C. Du , Energy Mater. 2023, 3, 300013.

[135] K. Jayaramulu , S. Mukherjee , D. M. Morales , D. P. Dubal , A. K. Nanjundan , A. Schneemann , J. Masa , S. Kment , W. Schuhmann , M. Otyepka , R. Zboril , R. A. Fischer , Chem. Rev. 2022, 122, 17241. 36318747 10.1021/acs.chemrev.2c00270 PMC9801388

[136] X. Mao , H. Li , J. Mater. Chem. B 2013, 1, 4267. 32261022 10.1039/c3tb20729c

[137] A. Doring , E. Ushakova , A. L. Rogach , Light Sci. Appl. 2022, 11, 75. 35351850 10.1038/s41377-022-00764-1 PMC8964749

[138] Suzuki , Y. Wang , P. Elvati , Z. B. Qu , K. Kim , S. Jiang , E. Baumeister , J. Lee , B. Yeom , J. H. Bahng , J. Lee , A. Violi , N. A. Kotov , ACS Nano 2016, 10, 1744. 26743467 10.1021/acsnano.5b06369

[139] E. E. Maroto , M. Izquierdo , S. Reboredo , J. Marco‐Martinez , S. Filippone , N. Martin , Acc. Chem. Res. 2014, 47, 2660. 25080165 10.1021/ar500201b

[140] M. Zhang , Y. Ma , H. Wang , B. Wang , Y. Zhou , Y. Liu , M. Shao , H. Huang , F. Lu , Z. Kang , ACS Appl. Mater. Interfaces 2021, 13, 5877. 33482691 10.1021/acsami.0c21949

[141] M. Zhang , W. Zhang , X. Fan , Y. Ma , H. Huang , X. Wang , Y. Liu , H. Lin , Y. Li , H. Tian , M. Shao , Z. Kang , Nano Lett. 2022, 22, 7203. 36000894 10.1021/acs.nanolett.2c02674

[142] S. S. Aloni , M. Perovic , M. Weitman , R. Cohen , M. Oschatz , Y. Mastai , Nanoscale Adv 2019, 1, 4981. 36133123 10.1039/c9na00520j PMC9419064

[143] H. Liu , D. Nishide , T. Tanaka , H. Kataura , Nat. Commun. 2011, 2, 309. 21556063 10.1038/ncomms1313 PMC3113293

[144] S. Ghosh , S. M. Bachilo , R. B. Weisman , Nat. Nano. 2010, 5, 443. 10.1038/nnano.2010.68 20453856

[145] K. Wang , G. J. Xia , T. Liu , Y. Yun , W. Wang , K. Cao , F. Yao , X. Zhao , B. Yu , Y. G. Wang , C. Jin , J. He , Y. Li , F. Yang , J. Am. Chem. Soc. 2023, 145, 12760. 37154477 10.1021/jacs.3c03128

[146] T. Kitanosono , P. Xu , S. Kobayashi , Science 2018, 362, 311. 30337405 10.1126/science.aap7883

[147] Y. Teng , J. Li , J. Yao , L. Kang , Q. Li , Microstructures 2023, 3, 2023019.

[148] Y. Wang , X. Cheng , K. Liu , X. Dai , J. Qi , Z. Ma , Y. Qiu , S. Liu , ACS Appl. Mater. Interfaces 2022, 14, 35809. 35912639 10.1021/acsami.2c09622

[149] X. Shan , K. Song , F. Shi , D. Zhao , Energ. Fuel. 2022, 36, 13382.

[150] K. Yuan , Y. Zeng , J. Gan , Z. Zhong , W. Xing , Ind. Eng. Chem. Res. 2022, 62, 247.

[151] A. Sanchez‐Castillo , C. Noguez , J. Phy. Chem. C 2010, 114, 9640.

[152] X. Tian , F. Meng , F. Meng , X. Chen , Y. Guo , Y. Wang , W. Zhu , Z. Zhou , ACS Appl. Mater. Interfaces 2017, 9, 15711. 28417637 10.1021/acsami.7b02607

[153] P. Han , K. Akagi , F. Federici Canova , H. Mutoh , S. Shiraki , K. Iwaya , P. S. Weiss , N. Asao , T. Hitosugi , ACS Nano 2014, 8, 9181. 25162921 10.1021/nn5028642

[154] X. Y. Wang , J. I. Urgel , G. B. Barin , K. Eimre , M. Di Giovannantonio , A. Milani , M. Tommasini , C. A. Pignedoli , P. Ruffieux , X. Feng , R. Fasel , K. Mullen , A. Narita , J. Am. Chem. Soc. 2018, 140, 9104. 29990420 10.1021/jacs.8b06210

[155] S. Ma , J. Gu , C. Lin , Z. Luo , Y. Zhu , J. Wang , J. Am. Chem. Soc. 2020, 142, 16887. 32900184 10.1021/jacs.0c08555

[156] J. Liu , W. Yuan , C. Li , M. Cheng , Y. Su , L. Xu , T. Chu , S. Hou , ACS Appl. Mater. Interfaces 2021, 13, 49215. 34628847 10.1021/acsami.1c14900

[157] Y. Zhang , G. Kim , Y. Zhu , C. Wang , R. Zhu , X. Lu , H. C. Chang , Y. Wang , ACS Nano 2023, 17, 10191. 37127891 10.1021/acsnano.3c00305

[158] X. Kang , E. R. Stephens , B. M. Spector‐Watts , Z. Li , Y. Liu , L. Liu , Y. Cui , Chem. Sci. 2022, 13, 9811. 36199638 10.1039/d2sc02436e PMC9431510

[159] L. Jiao , Y. Wang , H. L. Jiang , Q. Xu , Adv. Mater. 2018, 30, 1703663. 10.1002/adma.201703663 29178384

[160] Z. Sharifzadeh , K. Berijani , A. Morsali , Coord. Chem. Rev. 2021, 445, 214083.

[161] Y. Li , Y. Wu , T. Li , M. Lu , Yi Chen , Y. Cui , J. Gao , G. Qian , Carbon Energy 2023, 5, e265.

[162] Y. Sun , W. Wu , L. Yu , S. Xu , Y. Zhang , L. Yu , B. Xia , S. Ding , M. Li , LiLi Jiang , J. Duan , J. Zhu , S. Chen , Carbon Energy 2023, 5, e263.

[163] W. Gong , Z. Chen , J. Dong , Y. Liu , Y. Cui , Chem. Rev. 2022, 122, 9078. 35344663 10.1021/acs.chemrev.1c00740

[164] M. Ma , J. Chen , H. Liu , Z. Huang , F. Huang , Q. Li , Y. Xu , Nanoscale 2022, 14, 13405. 36070182 10.1039/d2nr01772e

[165] L. Zheng , F. Wang , C. Jiang , S. Ye , J. Tong , P. Dramou , H. He , Coord. Chem. Rev. 2022, 471, 214760.

[166] X. Kan , J. C. Wang , Z. Chen , J. Q. Du , J. L. Kan , W. Y. Li , Y. B. Dong , J. Am. Chem. Soc. 2022, 144, 6681. 35394764 10.1021/jacs.2c01186

[167] S. Zhuo , X. Wang , L. Li , S. Yang , Y. Ji , ACS Appl. Mater. Interfaces 2021, 13, 31059. 34169712 10.1021/acsami.1c09238

[168] C. Yuan , X. Wu , R. Gao , X. Han , Y. Liu , Y. Long , Y. Cui , J. Am. Chem. Soc. 2019, 141, 20187. 31789030 10.1021/jacs.9b10007

[169] J. Guo , Y. Duan , Y. Liu , H. Li , Y. Zhang , C. Long , Z. Wang , Y. Yang , S. Zhao , J. Mater. Chem. A 2022, 10, 6463.

[170] Y. Zhou , Y. Wei , J. Ren , X. Qu , Mater. Horiz 2020, 7, 3291.

[171] L. Cheng , Q. Guo , K. Zhao , Y. M. Li , H. Ren , C. Y. Ji , W. Li , Catal. Lett. 2022, 153, 1024.

[172] Y. M. Li , L. Cao , H. Ren , C. Y. Ji , W. Li , L. Cheng , Catal. Lett. 2022, 153, 1193.

[173] Y. Li , J. M. Wang , J. L. Kan , F. Li , Y. Dong , Y. B. Dong , Inorg. Chem. 2022, 61, 2455. 35061389 10.1021/acs.inorgchem.1c03268

[174] F. Li , J. L. Kan , B. J. Yao , Y. B. Dong , Angew. Chem., Int. Ed. 2022, 61, e202115044. 10.1002/anie.202115044 35357070

[175] S. Shi , Y. Zhong , Z. Hu , L. Wang , M. Yuan , S. Ding , S. Wang , C. Chen , Inorg. Chem. 2021, 60, 12714. 34424688 10.1021/acs.inorgchem.1c01831

[176] M. Kazem‐Rostami , A. Orte , A. M. Ortuno , A. H. G. David , I. Roy , D. Miguel , A. Garci , C. M. Cruz , C. L. Stern , J. M. Cuerva , J. F. Stoddart , J. Am. Chem. Soc. 2022, 144, 9380. 35595282 10.1021/jacs.2c01554

[177] P. C. Shi , D. H. Si , M. S. Yao , T. T. Liu , Y. B. Huang , T. Zhang , R. Cao , Sci. Chi. Mater. 2022, 65, 1531.

[178] Y. H. Xiao , Y. X. Zhang , R. Zhai , Z. G. Gu , J. Zhang , Sci. Chi. Mater. 2021, 65, 1269.

[179] R. Zhai , Y. Xiao , Z. Gu , J. Zhang , Nano Res. 2021, 15, 1102.

[180] H. Yang , J. Xu , H. Cao , J. Wu , D. Zhao , Nat. Commun. 2023, 14, 2726. 37169759 10.1038/s41467-023-38424-6 PMC10175538

[181] Y. Zhao , Y. Yang , J. Zhao , P. Weng , Q. Pang , Q. Song , Adv. Mater. 2016, 28, 4877. 27115447 10.1002/adma.201600369

[182] X. Wu , L. Xu , L. Liu , W. Ma , H. Yin , H. Kuang , L. Wang , C. Xu , N. A. Kotov , J. Am. Chem. Soc. 2013, 135, 18629. 24246036 10.1021/ja4095445

[183] Y. Zhao , L. Xu , L. M. Liz‐Marzán , H. Kuang , W. Ma , A. Asenjo‐García , F. J. García de Abajo , N. A. Kotov , L. Wang , C. Xu , J. Phy. Chem. Let. 2013, 4, 641. 10.1021/jz400045s 26281880

[184] Y. Zhao , M. Sun , W. Ma , H. Kuang , C. Xu , J. Phys. Chem. Lett. 2017, 8, 5633. 29094951 10.1021/acs.jpclett.7b01781

[185] Y. Zhao , L. Xu , W. Ma , L. Wang , H. Kuang , C. Xu , N. A. Kotov , Nano Lett. 2014, 14, 3908. 24857406 10.1021/nl501166m

[186] F. Gao , M. Sun , W. Ma , X. Wu , L. Liu , H. Kuang , C. Xu , Adv. Mater. 2017, 29, 1606864. 10.1002/adma.201606864 28230915

[187] D. Liang , Y. Xu , F. Peng , W. Ma , Y. Zhao , Chem. Eng. J. 2023, 474, 145933.

[188] X. Gao , X. Zhang , L. Zhao , P. Huang , B. Han , J. Lv , X. Qiu , S. H. Wei , Z. Tang , Nano Lett. 2018, 18, 6665. 30350652 10.1021/acs.nanolett.8b01001

[189] C. Zhou , X. Sun , J. Han , Mater. Chem. Front. 2020, 4, 2499.

[190] F. V. de Graaf , S. A. H. Jansen , T. Schnitzer , E. W. Meijer , G. Vantomme , J. Am. Chem. Soc. 2023, 145, 14379. 37342902 10.1021/jacs.3c03411 PMC10326880

[191] N. Liu , R. T. Gao , Z. Q. Wu , Acc. Chem. Res. 2023, 56, 2954. 37852202 10.1021/acs.accounts.3c00425

[192] B. Mahlmeister , T. Schembri , V. Stepanenko , K. Shoyama , M. Stolte , F. Würthner , J. Am. Chem. Soc. 2023, 145, 13302. 37285519 10.1021/jacs.3c03367

[193] P. Xing , Y. Zhao , Acc. Chem. Res. 2018, 51, 2324. 30179457 10.1021/acs.accounts.8b00312

[194] N. Micali , H. Engelkamp , P. G. van Rhee , P. C. M. Christianen , L. M. Scolaro , J. C. Maan , Nat. Chem. 2012, 4, 201. 22354434 10.1038/nchem.1264

[195] Z. Wang , A. Hao , P. Xing , Angew. Chem., Int. Ed. 2020, 59, 11556. 10.1002/anie.202003351 32270895

[196] J. Jiang , Y. Meng , L. Zhang , M. Liu , J. Am. Chem. Soc. 2016, 138, 15629. 27934018 10.1021/jacs.6b08808

[197] Z. Wang , Y. Li , A. Hao , P. Xing , Angew. Chem., Int. Ed. 2020, 60, 3138. 10.1002/anie.202011907 33151024

[198] Z. Wang , H. Zhang , A. Hao , Y. Zhao , P. Xing , Small 2020, 16, 2002036. 10.1002/smll.202002036 32578382

[199] M. Sun , S. Peng , L. Nie , Y. Zou , L. Yang , L. Gao , X. Dou , C. Zhao , C. Feng , ACS Nano 2021, 15, 14972. 34491712 10.1021/acsnano.1c05212

[200] S. Bhowmick , L. Zhang , G. H. Ouyang , M. H. Liu , ACS Omega 2018, 3, 8329. 31458965 10.1021/acsomega.8b00852 PMC6644911

[201] N. S. S. Nizar , M. Sujith , K. Swathi , C. Sissa , A. Painelli , K. G. Thomas , Chem. Soc. Rev. 2021, 50, 11208. 34522920 10.1039/d0cs01583k

[202] H. J. Cho , D. Y. Jeong , H. Moon , T. Kim , Y. K. Chung , Y. Lee , Z. Lee , J. Huh , Y. You , C. Song , Aggregate 2022, 3, e168.

[203] K. Velmurugan , A. Murtaza , A. Saeed , J. Li , K. Wang , M. Zuo , Q. Liu , X. Y. Hu , CCS Chemistry 2022, 4, 3426.

[204] S. Horiuchi , T. Yamaguchi , J. Tessarolo , H. Tanaka , E. Sakuda , Y. Arikawa , E. Meggers , G. H. Clever , K. Umakoshi , Nat. Commun. 2023, 14, 155. 36631447 10.1038/s41467-023-35850-4 PMC9834293

[205] L. Xu , C. Wang , Y. X. Li , X. H. Xu , L. Zhou , N. Liu , Z. Q. Wu , Angew. Chem., Int. Ed. 2020, 59, 16675. 10.1002/anie.202006561 32543000

[206] L. Yao , K. Fu , X. Wang , M. He , W. Zhang , P. Y. Liu , Y. P. He , G. Liu , ACS Nano 2023, 17, 2159. 36648130 10.1021/acsnano.2c08315

[207] J. Dong , Y. Liu , Y. Cui , Acc. Chem. Res. 2020, 54, 194. 33337867 10.1021/acs.accounts.0c00604

[208] P. W. N. M. Leeuwen , M. Raynal , Supramolecular catalysis: new directions and developments, Wiley, Hoboken 2021.

[209] C. García‐Simón , R. Gramage‐Doria , S. Raoufmoghaddam , T. Parella , M. Costas , X. Ribas , J. N. H. Reek , J. Am. Chem. Soc. 2015, 137, 2680. 25632976 10.1021/ja512637k

[210] G. Liu , J. Sheng , W. L. Teo , G. Yang , H. Wu , Y. Li , Y. Zhao , J. Am. Chem. Soc. 2018, 140, 16275. 30403348 10.1021/jacs.8b10024

[211] T. Hong , Z. Zhang , Y. Sun , J. J. Tao , J. D. Tang , C. Xie , M. Wang , F. Chen , S. S. Xie , S. Li , P. J. Stang , J. Am. Chem. Soc. 2020, 142, 10244. 32433874 10.1021/jacs.0c01563

[212] F. Nie , K. Z. Wang , D. Yan , Nat. Commun. 2023, 14, 1654. 36964159 10.1038/s41467-023-37331-0 PMC10039082

[213] J. Sun , Y. Li , F. Yan , C. Liu , Y. Sang , F. Tian , Q. Feng , P. Duan , L. Zhang , X. Shi , B. Ding , M. Liu , Nat. Commun. 2018, 9. 10.1038/s41467-018-05017-7 PMC6030102 29968753

[214] L. Primitivo , M. De Angelis , A. Necci , Di Pietro , A. Ricelli , D. Caschera , L. Pilloni , L. Suber , G. Righi , Nanoscale Adv. 2023, 5, 627. 36756516 10.1039/d2na00692h PMC9890582

[215] S. Ince , O. Oner , M. K. Yilmaz , M. Keles , B. Guzel , Inorg. Chem. 2023, 62, 4637. 36877595 10.1021/acs.inorgchem.3c00079 PMC10031557

[216] Y. Sun , C. Zhao , N. Gao , J. Ren , X. Qu , Chemistry 2017, 23, 18146. 29131418 10.1002/chem.201704579

[217] Y. Zhou , H. Sun , H. Xu , S. Matysiak , J. Ren , X. Qu , Angew. Chem., Int. Ed. 2018, 57, 16791. 10.1002/anie.201811118 30371985

[218] Y. Zhou , Y. Zou , J. Jiang , Mater. Lett. 2023, 331, 133432.

[219] C. Ding , W. L. Wei , H. J. Sun , J. H. Ding , J. S. Ren , X. G. Qu , Carbon 2014, 79, 615.

[220] S. Assavapanumat , S. Butcha , S. Ittisanronnachai , A. Kuhn , C. Wattanakit , Chem. Asian. J. 2021, 16, 3345. 34416087 10.1002/asia.202100966

[221] O. V. Fedorova , Y. A. Titova , A. Y. Vigorov , M. S. Toporova , O. A. Alisienok , A. N. Murashkevich , V. P. Krasnov , G. L. Rusinov , V. N. Charushin , Catal. Lett. 2016, 146, 493.

[222] R. Pourhasan‐Kisomi , F. Shirini , M. Golshekan , Silicon 2021, 14, 2583.

[223] S. Qiu , S. Y. Xu , Y. J. Wang , Y. G. Zheng , Chem. Eng. Sci. 2022, 261, 117935.

[224] T. S. Metzger , R. Siam , Y. Kolodny , N. Goren , N. Sukenik , S. Yochelis , R. Abu‐Reziq , D. Avnir , Y. Paltiel , J. Phys. Chem. Lett. 2021, 12, 5469. 34085834 10.1021/acs.jpclett.1c01518

[225] H. Sun , W. Ou , L. Sun , B. Wang , C. Su , Nano Res. 2022, 15, 10292.

[226] F. Zhang , Y. Sun , D. Tian , H. Li , Angew. Chem., Int. Ed. 2017, 56, 7186. 10.1002/anie.201701255 28481008

[227] K. Hou , J. Han , Z. Tang , ACS Mater. Lett. 2019, 2, 95.

[228] X. Chu , M. Wang , S. Shi , B. Sun , Q. Song , W. Xu , J. Shen , N. Zhou , J. Mater. Sci. 2022, 57, 12752.

[229] F. Li , S. Li , X. Guo , Y. Dong , C. Yao , Y. Liu , Y. Song , X. Tan , L. Gao , D. Yang , Angew. Chem., Int. Ed. 2020, 59, 11087. 10.1002/anie.202002904 32212366

[230] K. Szőri , B. Réti , G. Szőllősi , K. Hernádi , M. Bartók , Top. Catal. 2016, 59, 1227.

[231] C. Xing , J. Deng , R. Tan , M. Q. Gao , P. B. Hao , D. H. Yin , D. L. Yin , Catal. Sci. Technol. 2017, 7, 5944.

[232] C. Xu , C. Zhao , M. Li , L. Wu , J. Ren , X. Qu , Small 2014, 10, 1841. 24523073 10.1002/smll.201302750

[233] Y. Zhang , Q. Li , G. Zhang , T. Lv , P. Geng , Y. Chen , Energy Mater 2023, 3, 300022.

[234] J. Guo , Y. Zhang , Y. Zhu , C. Long , M. Zhao , M. He , X. Zhang , J. Lv , B. Han , Z. Tang , Angew. Chem., Int. Ed. 2018, 57, 6873. 10.1002/anie.201803125 29664164

[235] K. Berijani , A. Morsali , J. T. Hupp , Catal. Sci. Technol. 2019, 9, 3388.

[236] M. Sha , L. Rao , W. Xu , Y. Qin , R. Su , Y. Wu , Q. Fang , H. Wang , X. Cui , L. Zheng , W. Gu , C. Zhu , Nano Lett. 2023, 23, 701. 36598260 10.1021/acs.nanolett.2c04697

[237] Y. Zhang , J. Guo , J. Zhang , X. Qiu , X. Zhang , J. Han , B. Zhang , C. Long , Y. Shi , Z. Yang , W. Zhao , Z. Tang , Chem 2022, 8, 1688.

[238] X. Zhang , L. Jing , L. Wei , F. Zhang , H. Yang , ACS Catal. 2017, 7, 6711.

[239] Y. Zhang , S. Chen , A. M. Al‐Enizi , A. Nafady , Z. Tang , S. Ma , Angew. Chem., Int. Ed. 2023, 62, e202213399. 10.1002/anie.202213399 36347776

[240] Y. Zhang , Y. Jiang , A. Nafady , Z. Tang , A. M. Al‐Enizi , K. Tan , S. Ma , ACS Cent. Sci. 2023, 9, 1692. 37637733 10.1021/acscentsci.3c00637 PMC10451035

[241] Y. Zhang , J. Guo , L. Shi , Y. Zhu , K. Hou , Y. Zheng , Z. Tang , Sci. Adv. 2017, 3, e1701162. 28835929 10.1126/sciadv.1701162 PMC5562422

[242] Y. Feng , R. Shi , M. Yang , Y. Zheng , Z. Zhang , Y. Chen , Angew. Chem., Int. Ed. 2023, 62, e202302436. 10.1002/anie.202302436 36916443

[243] H. Liao , Y. J. Chou , Y. Wang , H. Zhang , T. Y. Cheng , G. H. Liu , ChemCatChem 2017, 9, 3197.

[244] J. C. Wang , X. Kan , J. Y. Shang , H. Qiao , Y. B. Dong , J. Am. Chem. Soc. 2020, 142, 16915. 32941016 10.1021/jacs.0c07461

[245] H. C. Ma , C. C. Zhao , G. J. Chen , Y. B. Dong , Nat. Commun. 2019, 10, 3368. 31358761 10.1038/s41467-019-11355-x PMC6662712

[246] J. Xu , Y. Zhang , X. Zhu , T. Long , He Xu , X. Lou , Z. Xu , Hu Fu , W. Xiang , M. Xie , C. Jia , J. Cent. South Univ. 2022, 29, 2956.

[247] R. de la Serna , D. Nieto , R. Sainz , B. Bernardo‐Maestro , A. Mayoral , C. Marquez‐Alvarez , J. Perez‐Pariente , L. Gomez‐Hortiguela , J. Am. Chem. Soc. 2022, 144, 8249. 35502872 10.1021/jacs.2c01874 PMC9100664

[248] S. Song , J. Wang , N. Song , H. Di , D. Liu , Z. Yu , Nanoscale 2020, 12, 2422. 31916547 10.1039/c9nr09492j

[249] Z. Shen , Y. Sang , T. Wang , J. Jiang , Y. Meng , Y. Jiang , K. Okuro , T. Aida , M. Liu , Nat. Commun. 2019, 10, 3976. 31484928 10.1038/s41467-019-11840-3 PMC6726595

[250] Y. F. Xue , J. Liu , Q. Ge , N. Jiang , W. F. Zhao , M. Liu , H. Cong , J. L. Zhao , Org. Lett. 2023, 25, 2632. 37036807 10.1021/acs.orglett.3c00653

[251] Z. He , T. Miao , X. Cheng , H. Ma , Y. Ma , W. Zhang , X. Zhu , Polym. Chem. 2022, 13, 1953.

[252] B. Altava , M. I. Burguete , E. Garcia‐Verdugo , S. V. Luis , Chem. Soc. Rev. 2018, 47, 2722. 29577129 10.1039/c7cs00734e

[253] J. Jiang , G. H. Ouyang , L. Zhang , M. H. Liu , Chem. ‐ Eur. J. 2017, 23, 9439. 28342230 10.1002/chem.201700727

[254] S. Liu , M. Tang , J. Pang , J. Hu , W. Chen , J. Cheng , Z. Liu , H. Zhao , R. Tan , ACS Sustainable Chem. Eng. 2022, 10, 11760.

[255] J. Han , H. Gong , X. Ren , X. Yan , Nano Today 2021, 41, 101295.

[256] S. A. A. Razavi , K. Berijani , A. Morsali , Catal. Sci. Technol. 2020, 10, 8240.

[257] L. Xu , M. Guo , C. T. Hung , X. L. Shi , Y. Yuan , X. Zhang , R. H. Jin , W. Li , Q. Dong , D. Zhao , J. Am. Chem. Soc. 2023, 145, 7810. 37002870 10.1021/jacs.2c12214

[258] A. Qu , X. Wu , S. Li , M. Sun , L. Xu , H. Kuang , C. Xu , Adv. Mater. 2020, 32, 2000184. 10.1002/adma.202000184 32100405

[259] M. Lu , A. Qu , S. Li , M. Sun , L. Xu , H. Kuang , C. Xu , Angew. Chem., Int. Ed. 2020, 59, 8698. 10.1002/anie.202002576 32119165

[260] S. Li , L. Xu , M. Lu , M. Sun , L. Xu , C. Hao , X. Wu , C. Xu , H. Kuang , Nano Res. 2021, 14, 2451.

[261] M. Sun , T. Hao , X. Li , A. Qu , L. Xu , C. Hao , C. Xu , H. Kuang , Nat. Commun. 2018, 9, 4494. 30374052 10.1038/s41467-018-06946-z PMC6206072

[262] F. Wang , Y. Zhang , Z. Du , J. Ren , X. Qu , Nat. Commun. 2018, 9, 1209. 29572444 10.1038/s41467-018-03617-x PMC5865172

[263] Z. Du , C. Liu , H. Song , P. Scott , Z. Liu , J. Ren , X. Qu , Chem 2020, 6, 2060.

[264] I. E. Palama , F. Di Maria , M. Zangoli , S. D'Amone , G. Manfredi , J. Barsotti , G. Lanzani , L. Ortolani , E. Salatelli , G. Gigli , G. Barbarella , RSC Adv. 2019, 9, 23036. 35514476 10.1039/c9ra04782d PMC9067287

[265] J. P. C. Coverdale , I. Romero‐Canelon , C. Sanchez‐Cano , G. J. Clarkson , A. Habtemariam , M. Wills , P. J. Sadler , Nat. Chem. 2018, 10, 347. 29461524 10.1038/nchem.2918

